# GPS data from sea ice trackers deployed in Fram Strait in 2016

**DOI:** 10.1016/j.dib.2018.04.109

**Published:** 2018-05-04

**Authors:** Stefan Muckenhuber, Hanne Sagen

**Affiliations:** Nansen Environmental and Remote Sensing Center (NERSC), Thormøhlensgate 47, 5006 Bergen, Norway

## Abstract

Three GPS trackers have been deployed on sea ice in Fram Strait collecting GPS positions between 7th July 2016 until 10th September 2016 with an interval between 5 and 30 min. For an easy understanding and usage, corresponding satellite images and python scripts are added to the GPS data.

**Specifications Table**

**Table:** 

Subject area	Physics
More specific subject area	Sea ice
Type of data	Text/excel files, python scripts, plots, satellite images
How data was acquired	ROVER Surface Iridium Satellite Beacon with GPS Location from Xeos Technology
Data format	Raw, filtered and analyzed
Experimental factors	GPS positions recorded before deployment were removed
Experimental features	GPS tracker were placed on wooden construction on sea ice and GPS position data was delivered via Iridium satellites
Data source location	Fram Strait
Data accessibility	With this article
Related research article	None

**Value of the data**

● The data can be used to gain a better understanding of short-term sea ice drift variability and to investigate ice drift on a high temporal resolution.
● The data allows to validate sea ice drift algorithms in Fram Strait and can potentially show how displacements from subsequent satellite images relate to instantaneous sea ice velocity.
● The attached python scripts can be used to work with other GPS buoy data.

## 1 Data

Fram Strait represents a major gateway between the Arctic Ocean and the Atlantic Ocean, both in terms of oceanic heat transport into the Arctic and sea ice export into lower latitudes. About 10% of the Arctic sea ice cover is exported through Fram Strait every year [1].

GPS trackers have been deployed on wooden constructions on loose ice floes and pack-ice in Western Fram Strait during the UNDER-ICE cruise of KV Svalbard in July 2016.

A total of three GPS trackers (NERSC 1, NERSC 2 and NERSC 3) have been deployed on sea ice between 7th and 8th July 2016, collecting GPS data until 10th September 2016 with an interval between 5 and 30 min. For further technical specifications about the trackers see the user manual from Xeos Technologies [2].

**Table of GPS trackers**

**Table:** 

GPS tracker	Serial number	Deployment (UTC)	Last data collection (UTC)
NERSC 1	SN0269	08.07.2016, 11:29	17.07.2016, 08:20
NERSC 2	SN0268	07.07.2016, 15:52	10.09.2016, 09:32
NERSC 3	SN0267	07.07.2016, 18:55	18.08.2016, 20:32

## 2 Experimental design, materials, and methods

**Fig. 1:**
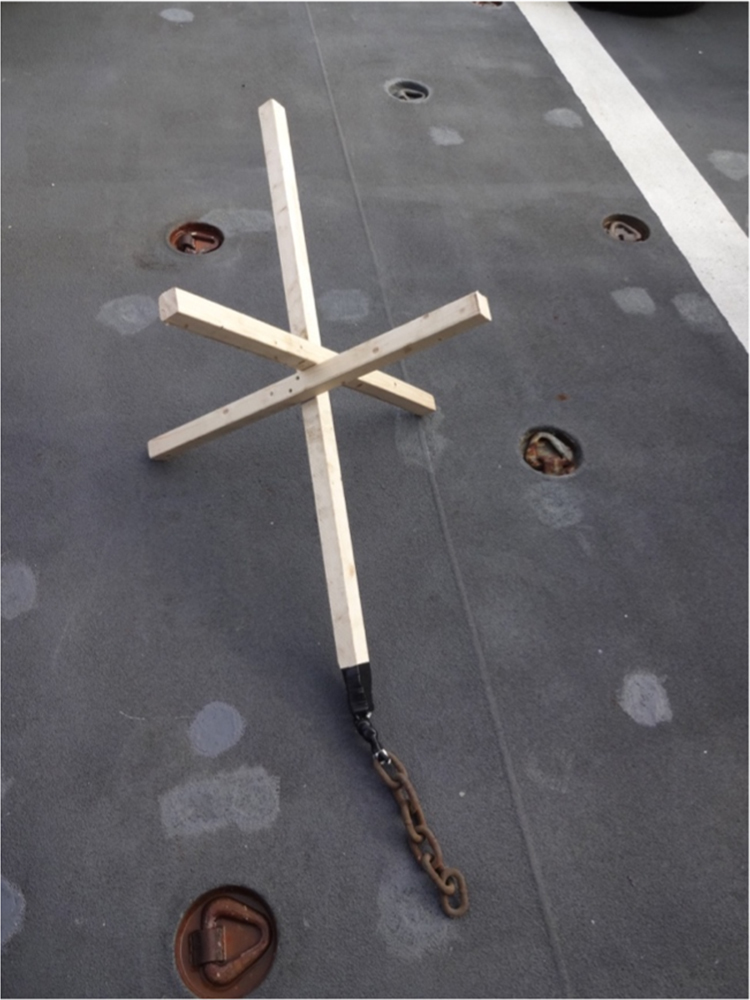
Wooden construction including heavy metal chain before deployment.

**Fig. 2:**
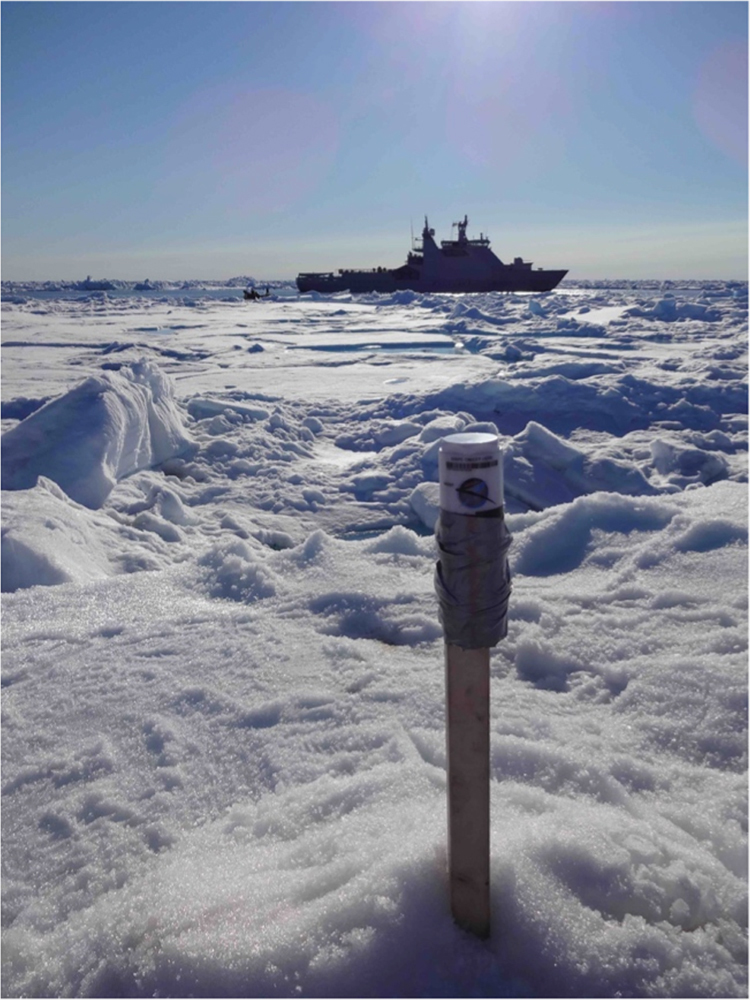
GPS tracker mounted on wooden construction after deployment using tape and metal clips. KV Svalbard in the background.

Three wooden constructions were built including a heavy metal chain on the lower end to prevent tipping over (Fig. 1). The wooden constructions were buried into sea ice and the GPS trackers were mounted on top of the constructions using tape and metal clips (Fig. 2). The GPS tracker were then left alone and sent their positions in intervals between 5 and 30 min via iridium satellites.

To provide a good overview of the sea ice situation around the GPS trackers, optical satellite images from MODIS, Landsat-8 and Sentinel-2 were collected. The positions of the three trackers are indicated on the attached satellite images using the same color coding as in the plots.

To compare the collected high-resolution sea ice drift data from Fram Strait with the situation in the central Arctic, we got access to the hovercraft GPS data from the FRAM 2014/15 expedition (http://www.geonova.no/diaries/sabvabaa/). From August 2014, Yngve Kristoffersen and Audun Tholfsen were drifting on an ice station including the hovercraft 'Sabvabaa' for 353 days over a distance of 2.200 km along the submarine Lomonosov Ridge. The hovercraft GPS data used in this document was collected between 31st August 2014 and 6th July 2015 with a temporal resolution of 10 s.

**Fig. 3:**
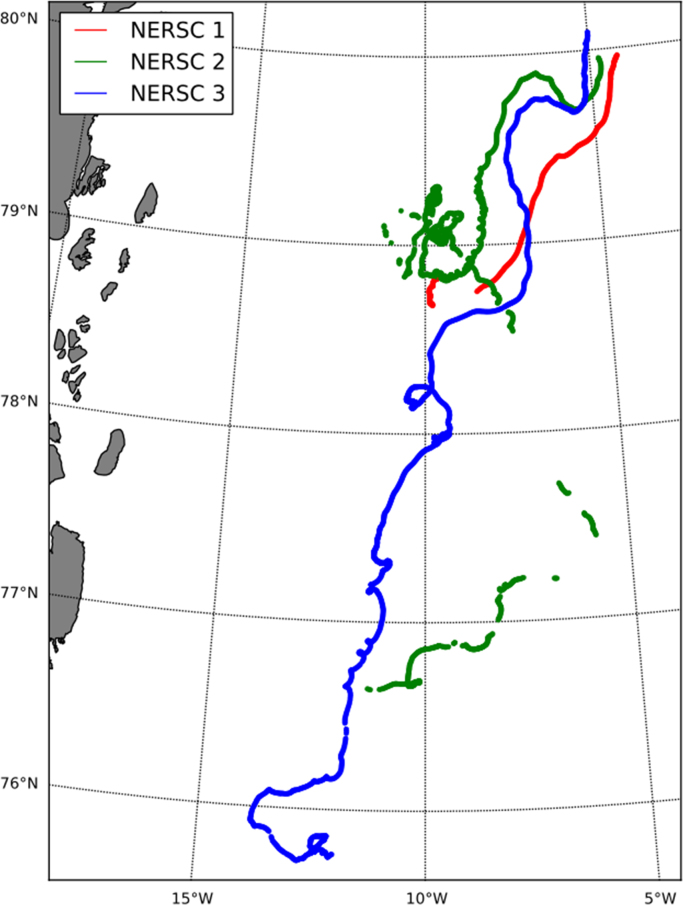
Drift of GPS sea ice trackers NERSC 1, NERSC 2 and NERSC 3.

**Fig. 4:**
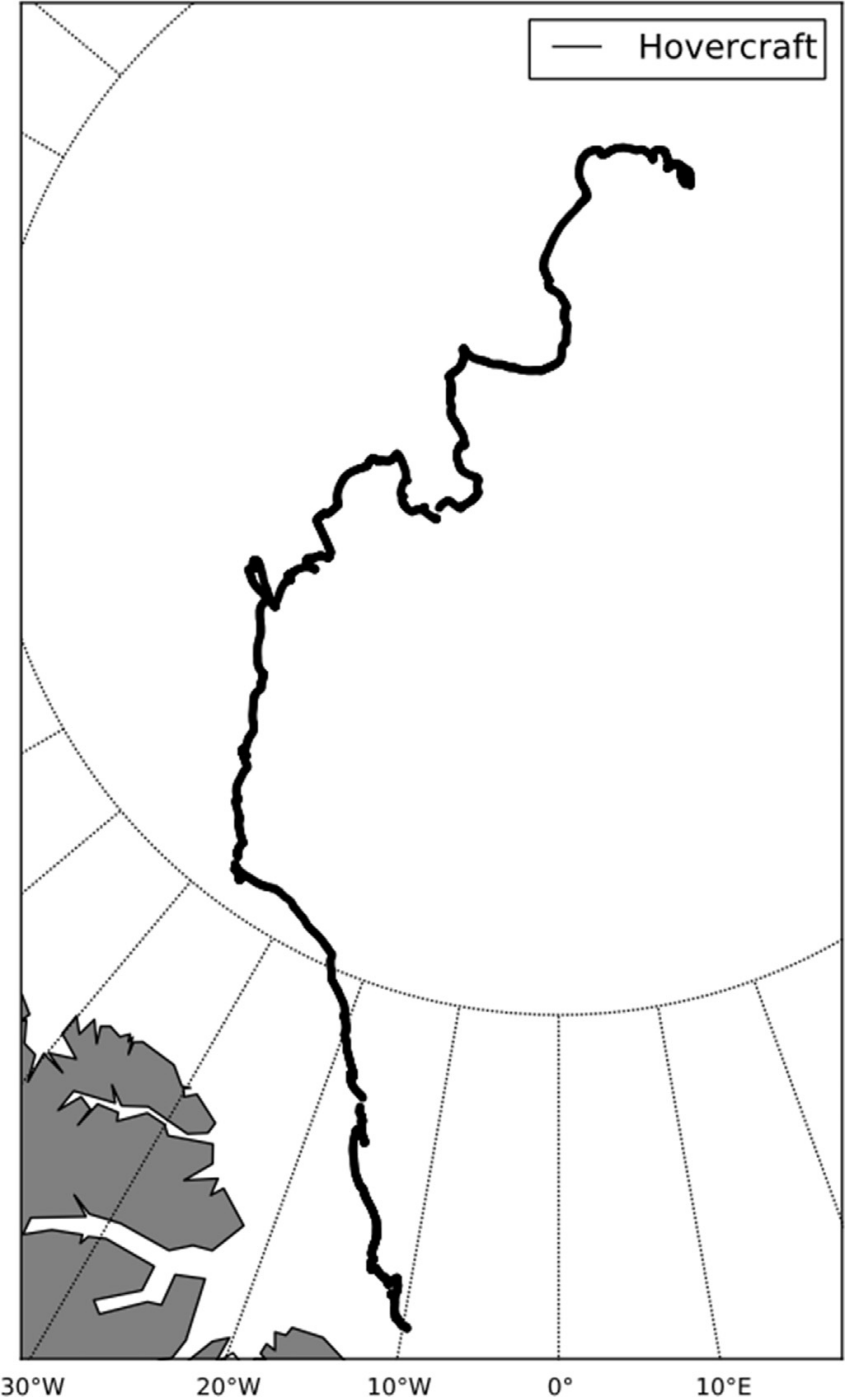
Drift of hovercraft during FRAM 2014/15 expedition.

**Fig. 5:**
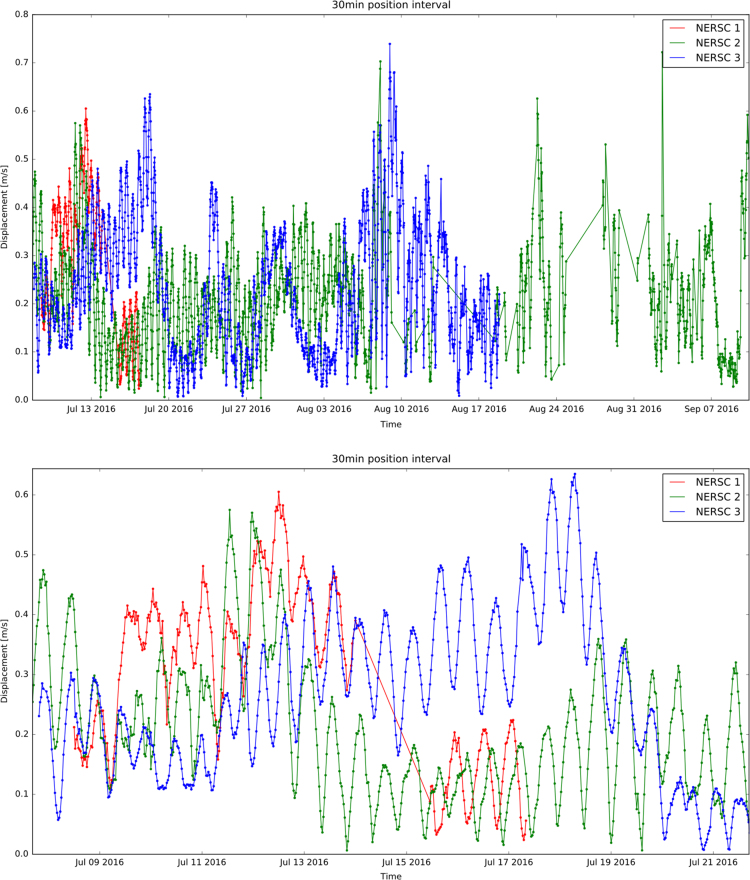
Displacement velocity versus time of GPS sea ice trackers NERSC 1, NERSC 2 and NERSC 3 considering a 30 min time interval between position acquisitions.

**Fig. 6:**
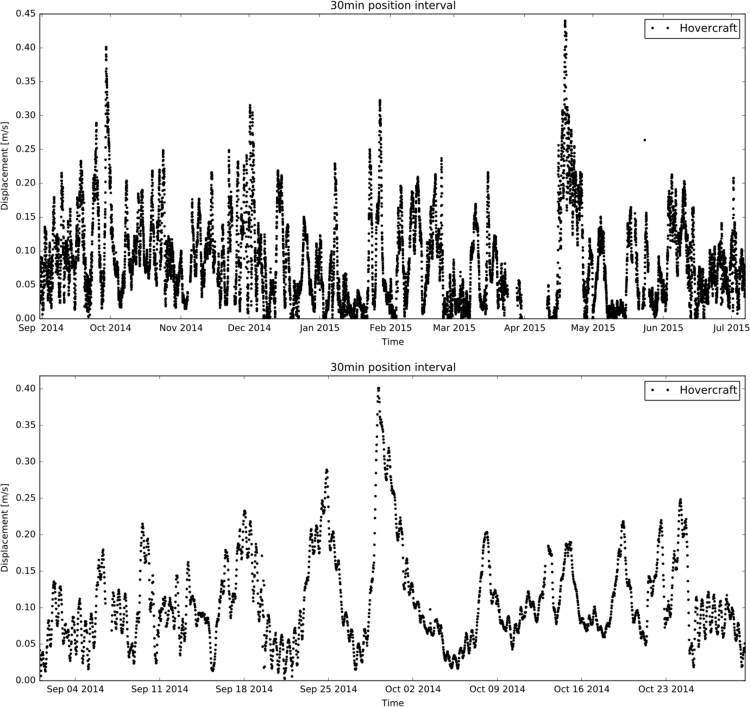
Displacement velocity versus time of hovercraft considering a 30 min time interval between position acquisitions.

**Fig. 7:**
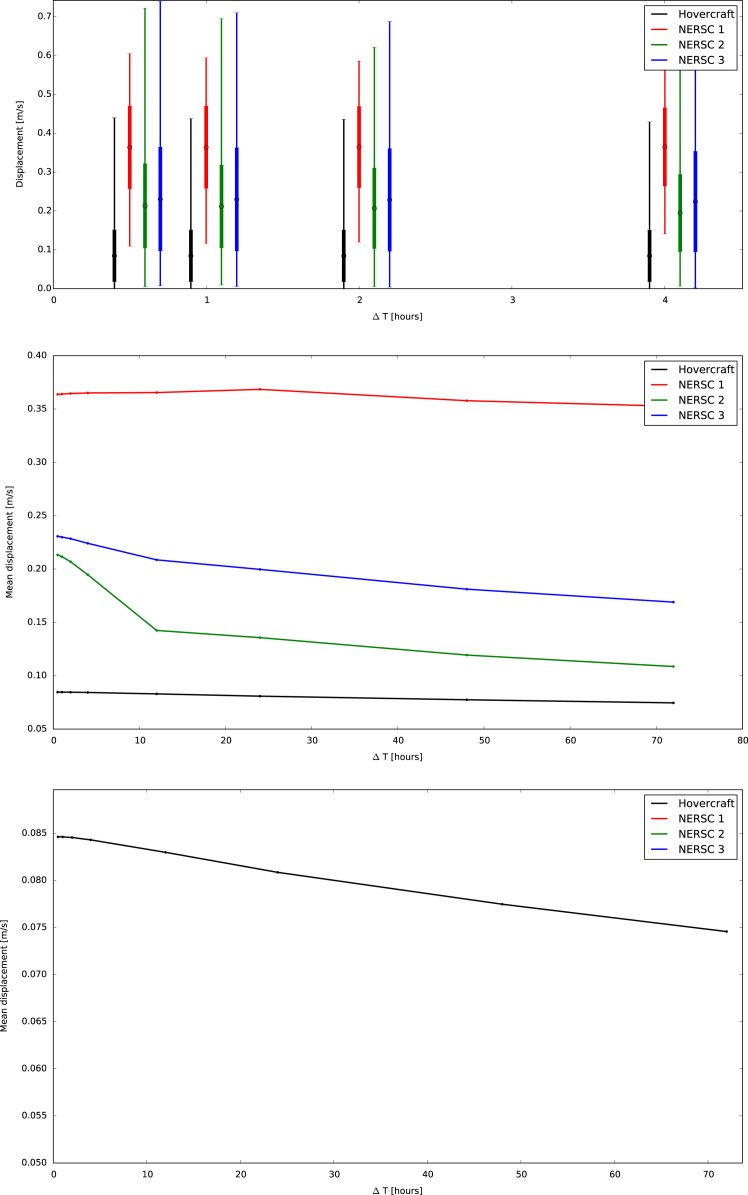
Displacement velocity distribution with mean, standard deviation and range (uppermost panel) and average displacement velocity (middle and lower panel) versus time difference Δ*t* [h] of GPS ice trackers and hovercraft.

**Fig. 8:**
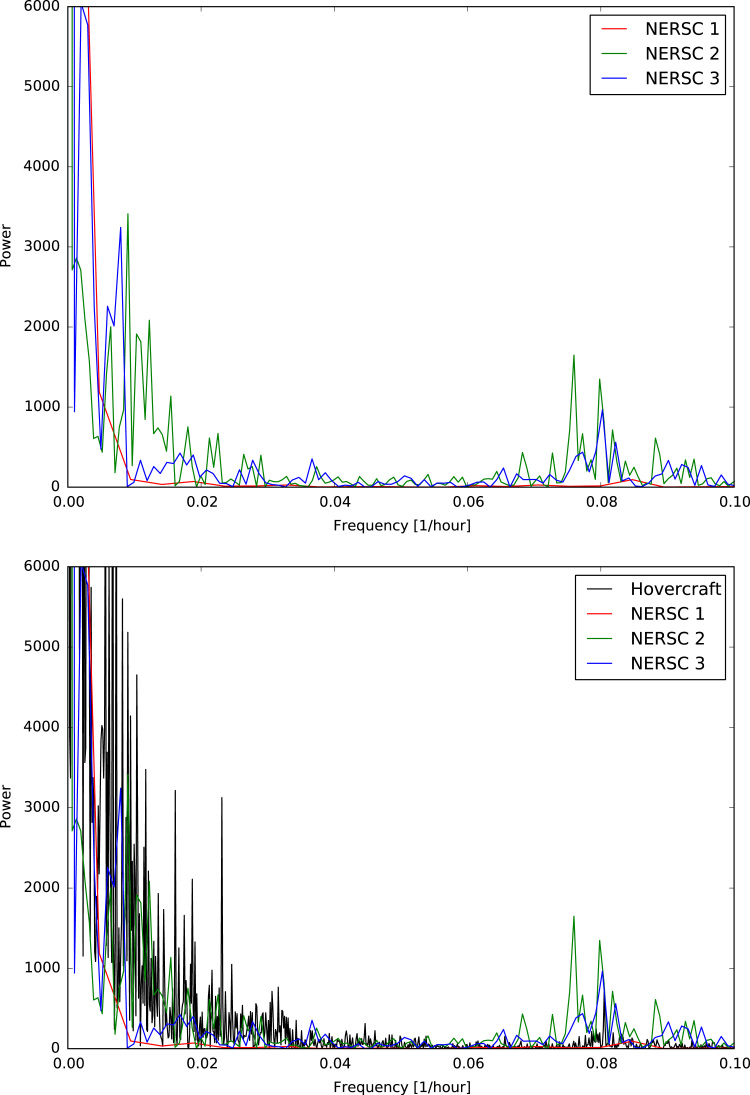
Power spectral density distribution of GPS ice trackers (upper panel) and incl. hovercraft (lower panel).

Fig. 3, Fig. 4 show the trajectories of the three GPS trackers NERSC 1, NERSC 2, NERSC 3 and the drift of the hovercraft. Fig. 5, Fig. 6 depict the temporal evolution of the displacement velocity in m/s of the three GPS trackers and the hovercraft considering a 30 min time interval between position acquisitions. Fig. 7 shows the displacement velocity in meters per second of GPS ice trackers and hovercraft versus the chosen time difference Δ*t*, ranging from 5 min to 72 h. Fig. 8 depicts the power spectral density distribution of the GPS trackers and the hovercraft.
