# Supplementary figures and images for: GPS data from sea ice trackers deployed in Fram Strait in 2016
Source: Data Brief. 2018 May 4;18:2000–9. doi: 10.1016/j.dib.2018.04.109 (PMC5999012; doi:10.1016/j.dib.2018.04.109)

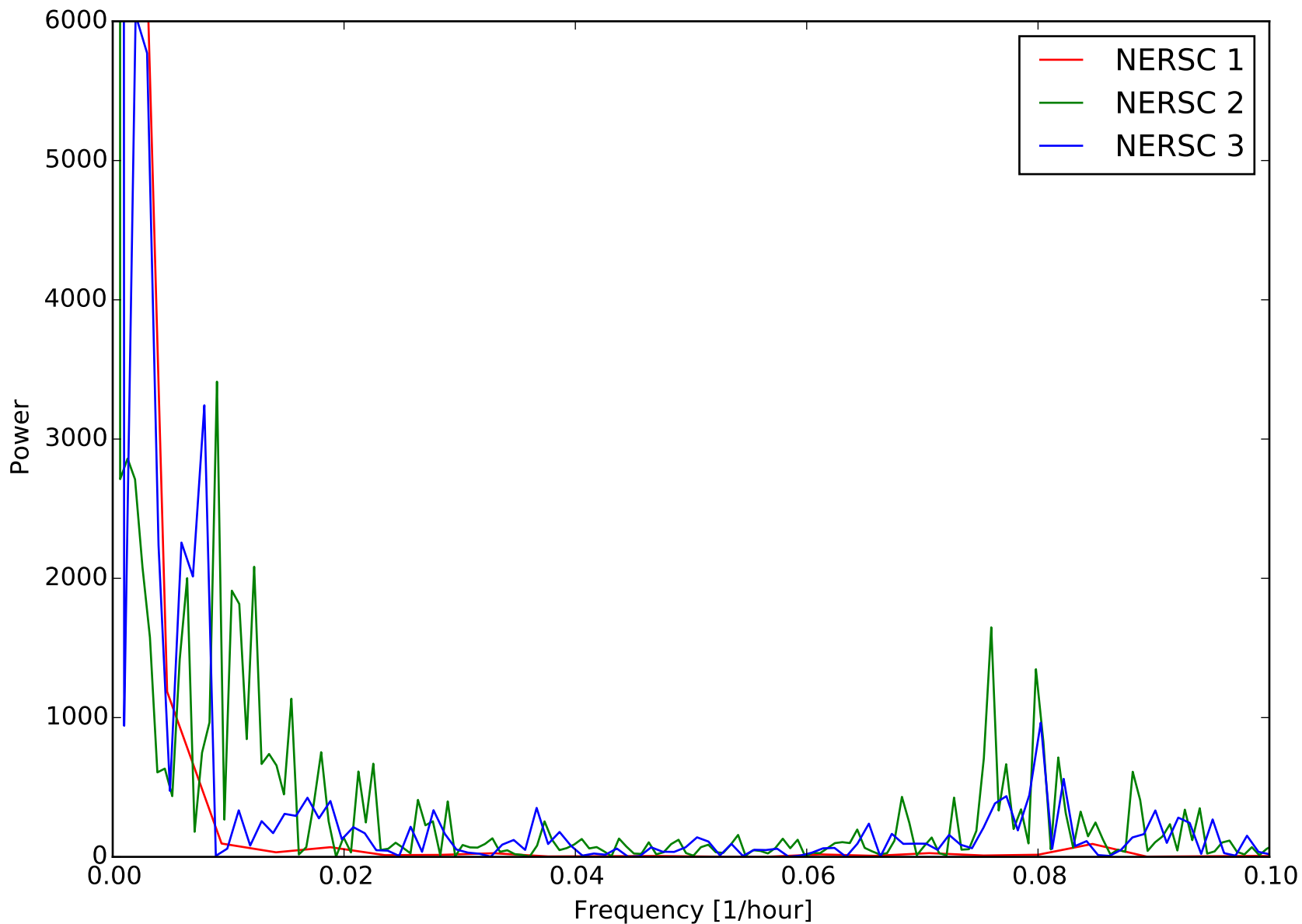

Supplement: Supplementary file 2 — Supplementary material [file mmc2.zip › GPS_tracker_data_python_plots_satellite/GPS_tracker_plots/PSD_01.pdf]

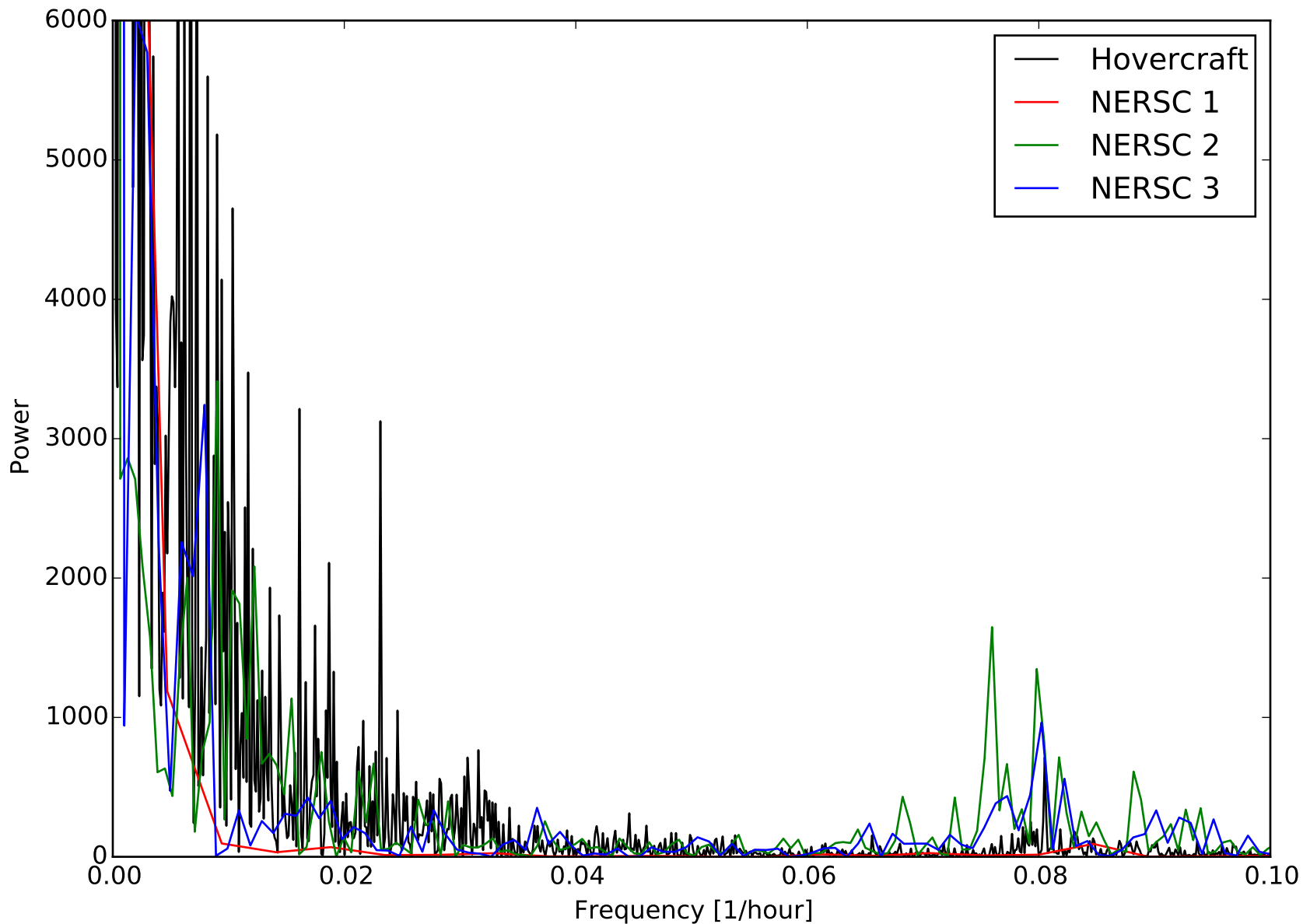

Supplement: Supplementary file 2 — Supplementary material [file mmc2.zip › GPS_tracker_data_python_plots_satellite/GPS_tracker_plots/PSD_02.pdf]

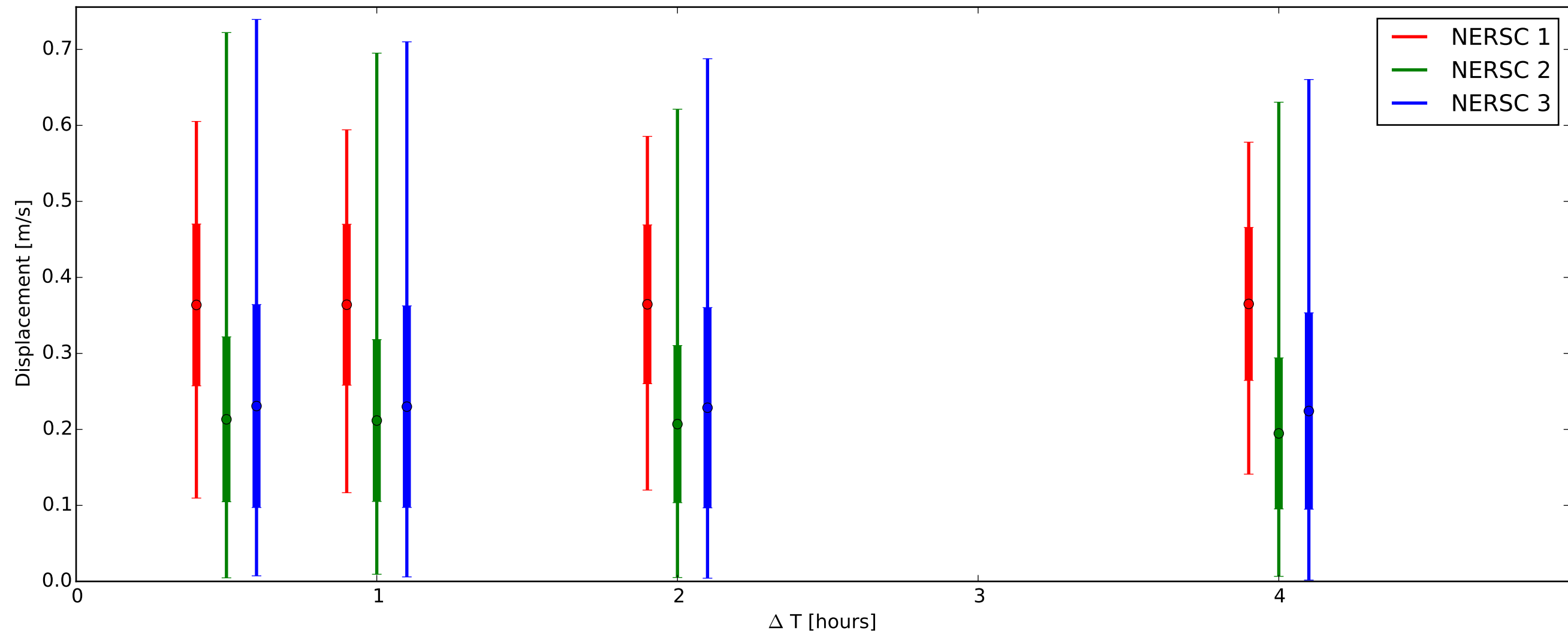

Supplement: Supplementary file 2 — Supplementary material [file mmc2.zip › GPS_tracker_data_python_plots_satellite/GPS_tracker_plots/spd_dt_01.pdf]

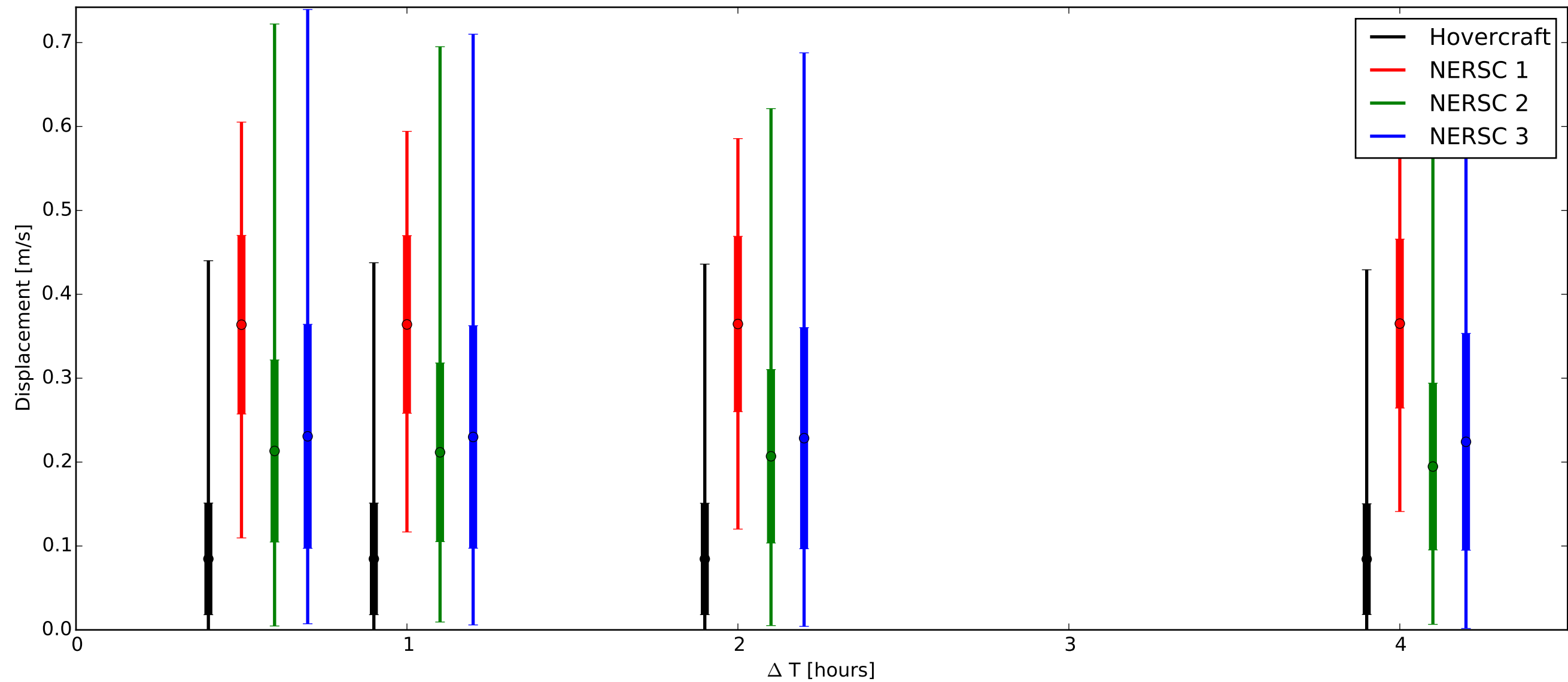

Supplement: Supplementary file 2 — Supplementary material [file mmc2.zip › GPS_tracker_data_python_plots_satellite/GPS_tracker_plots/spd_dt_02.pdf]

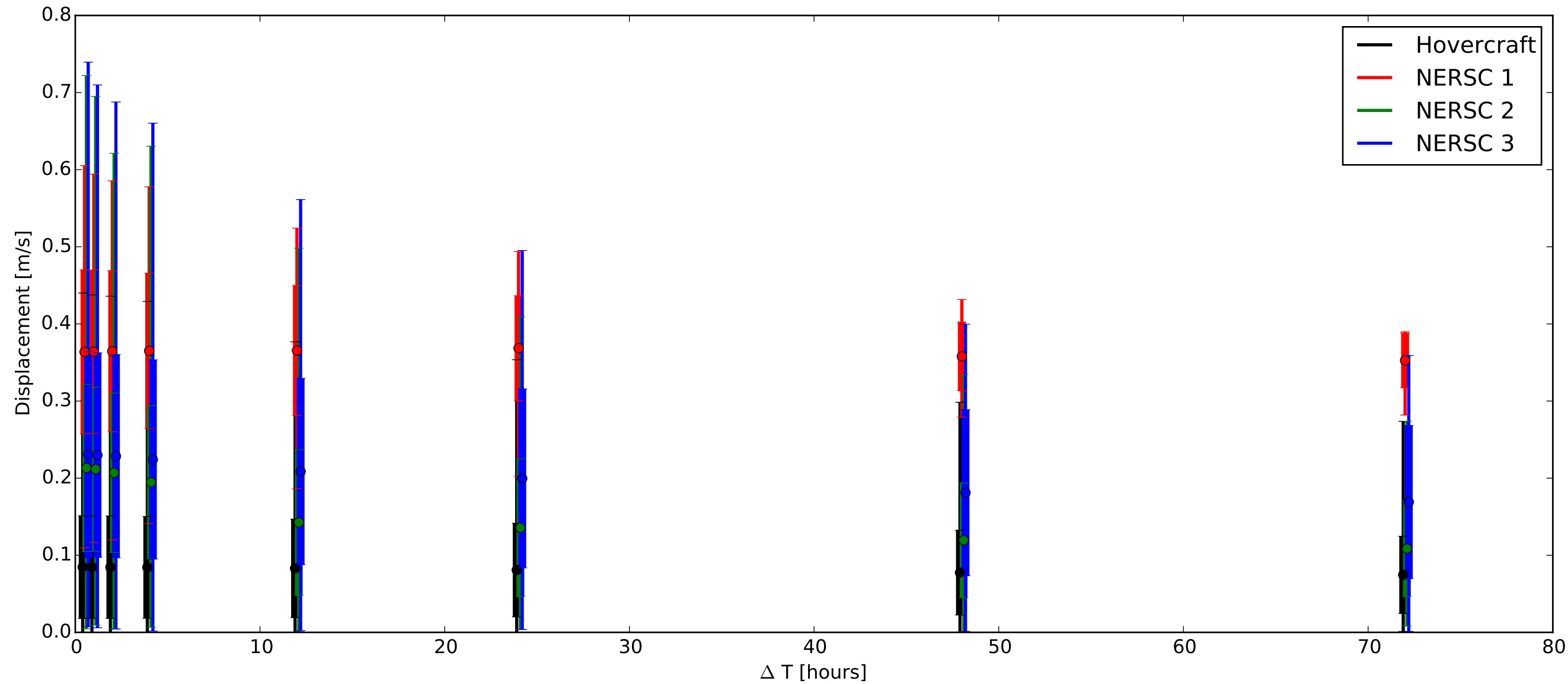

Supplement: Supplementary file 2 — Supplementary material [file mmc2.zip › GPS_tracker_data_python_plots_satellite/GPS_tracker_plots/spd_dt_03.pdf]

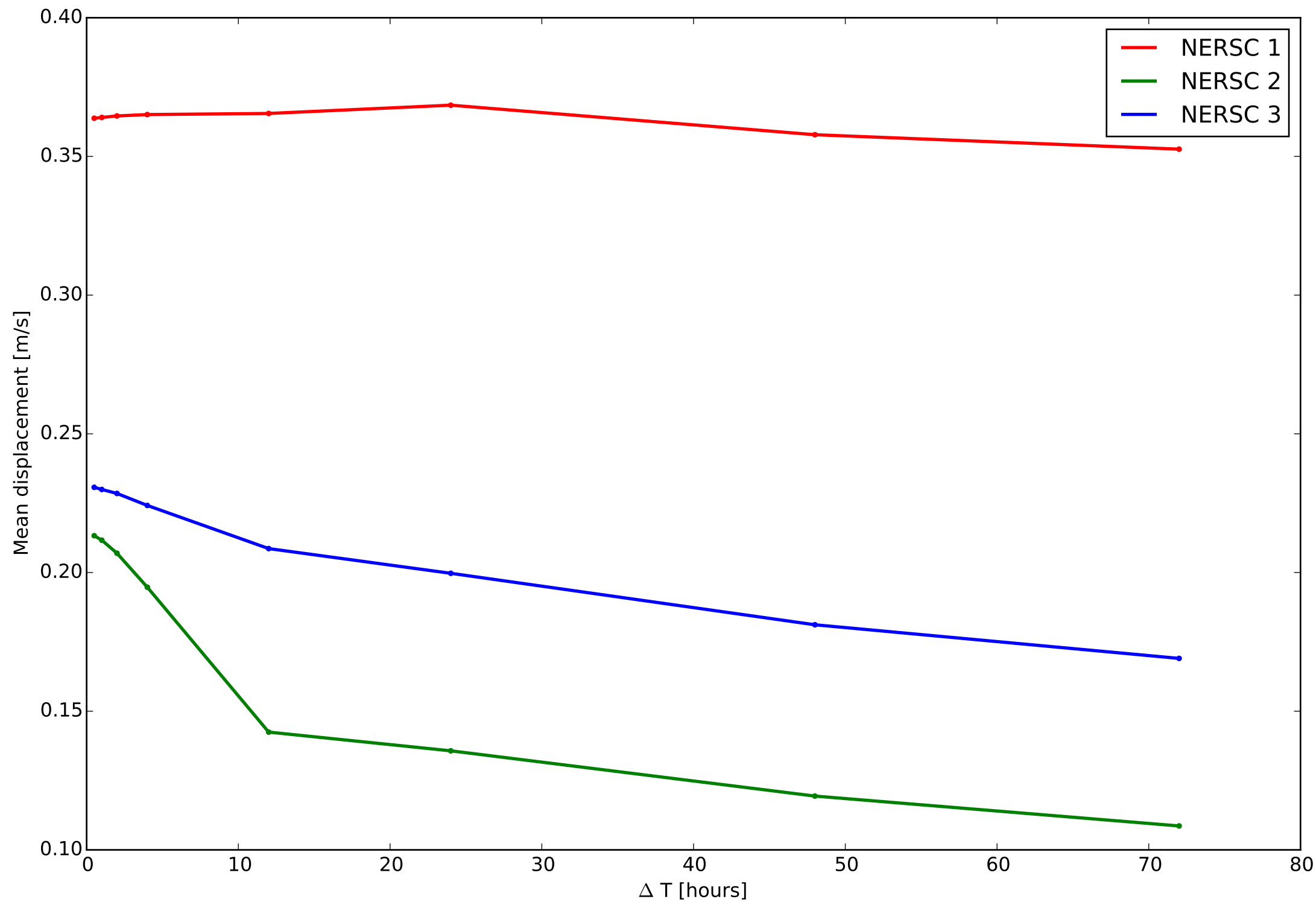

Supplement: Supplementary file 2 — Supplementary material [file mmc2.zip › GPS_tracker_data_python_plots_satellite/GPS_tracker_plots/spd_dt_mean_01.pdf]

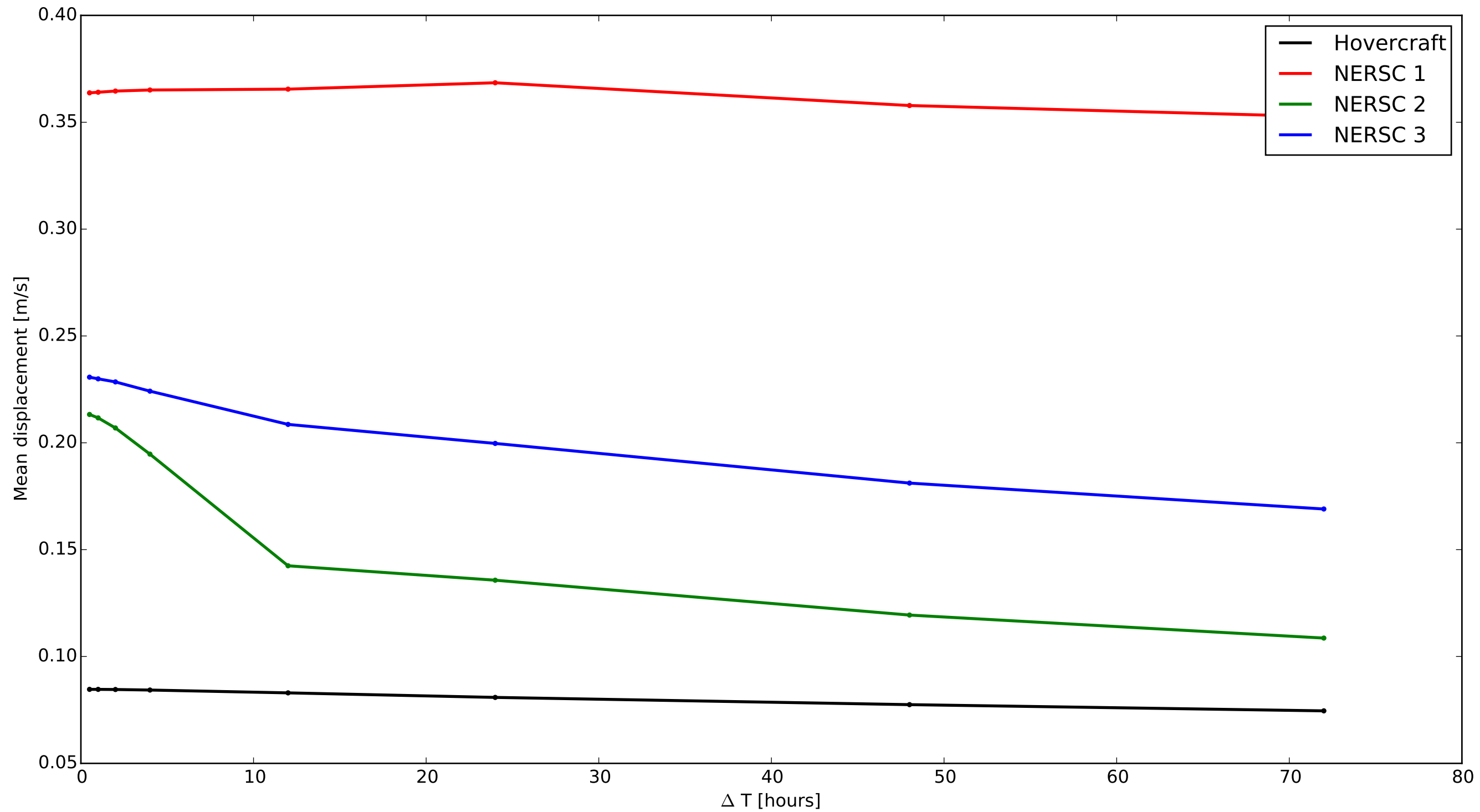

Supplement: Supplementary file 2 — Supplementary material [file mmc2.zip › GPS_tracker_data_python_plots_satellite/GPS_tracker_plots/spd_dt_mean_02.pdf]

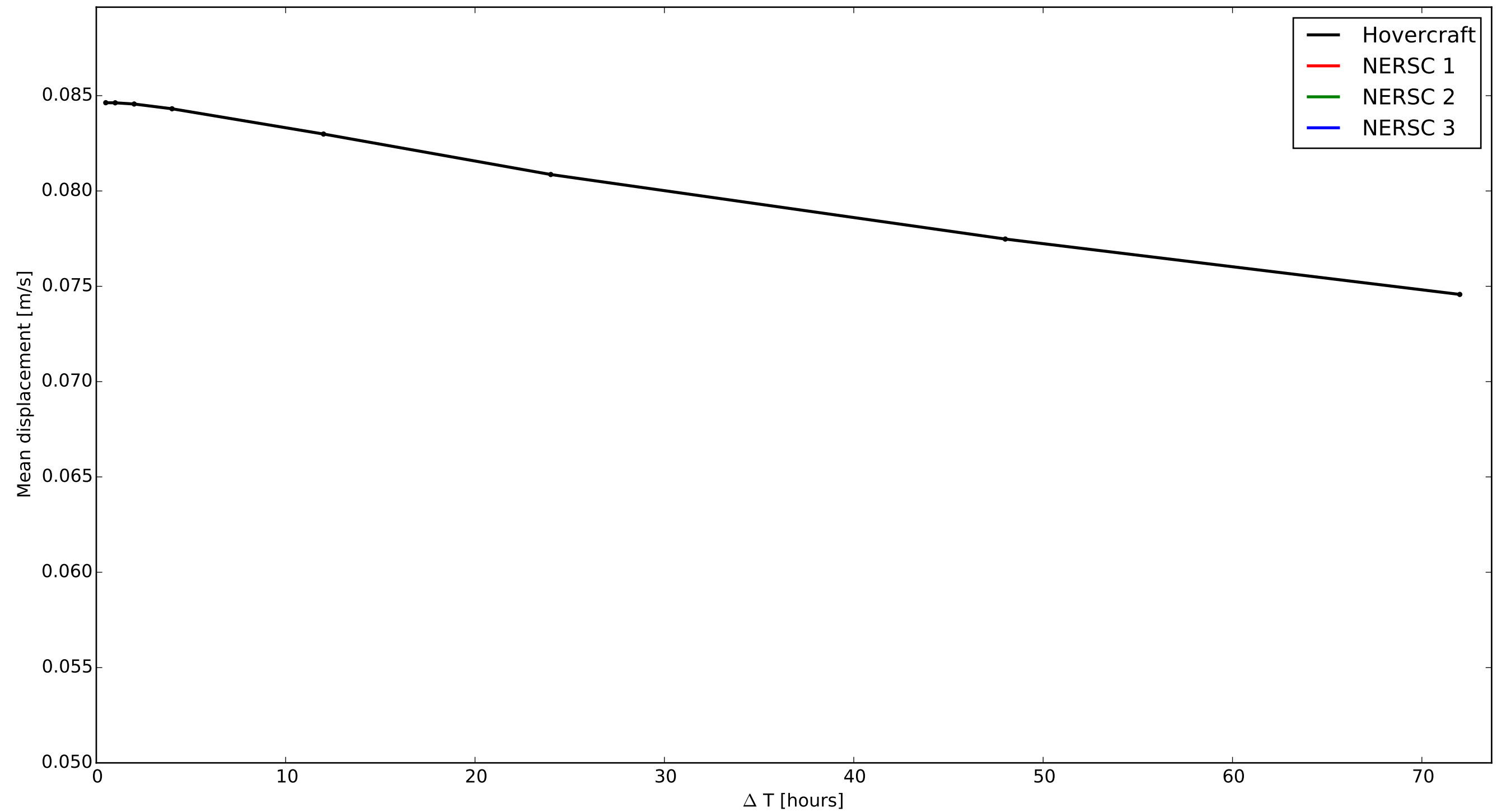

Supplement: Supplementary file 2 — Supplementary material [file mmc2.zip › GPS_tracker_data_python_plots_satellite/GPS_tracker_plots/spd_dt_mean_03.pdf]

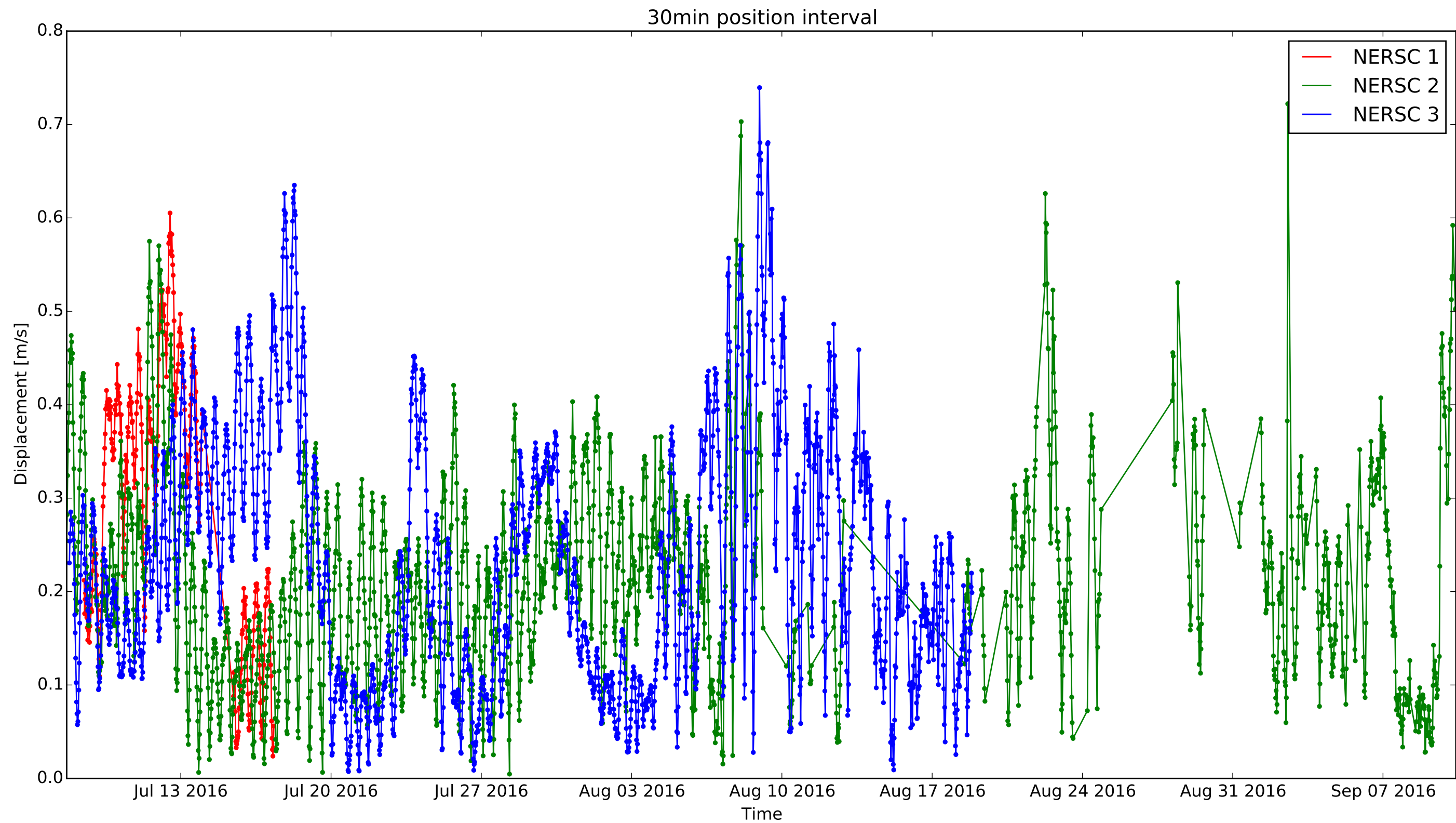

Supplement: Supplementary file 2 — Supplementary material [file mmc2.zip › GPS_tracker_data_python_plots_satellite/GPS_tracker_plots/timeseries_30min_01.pdf]

30min position interval

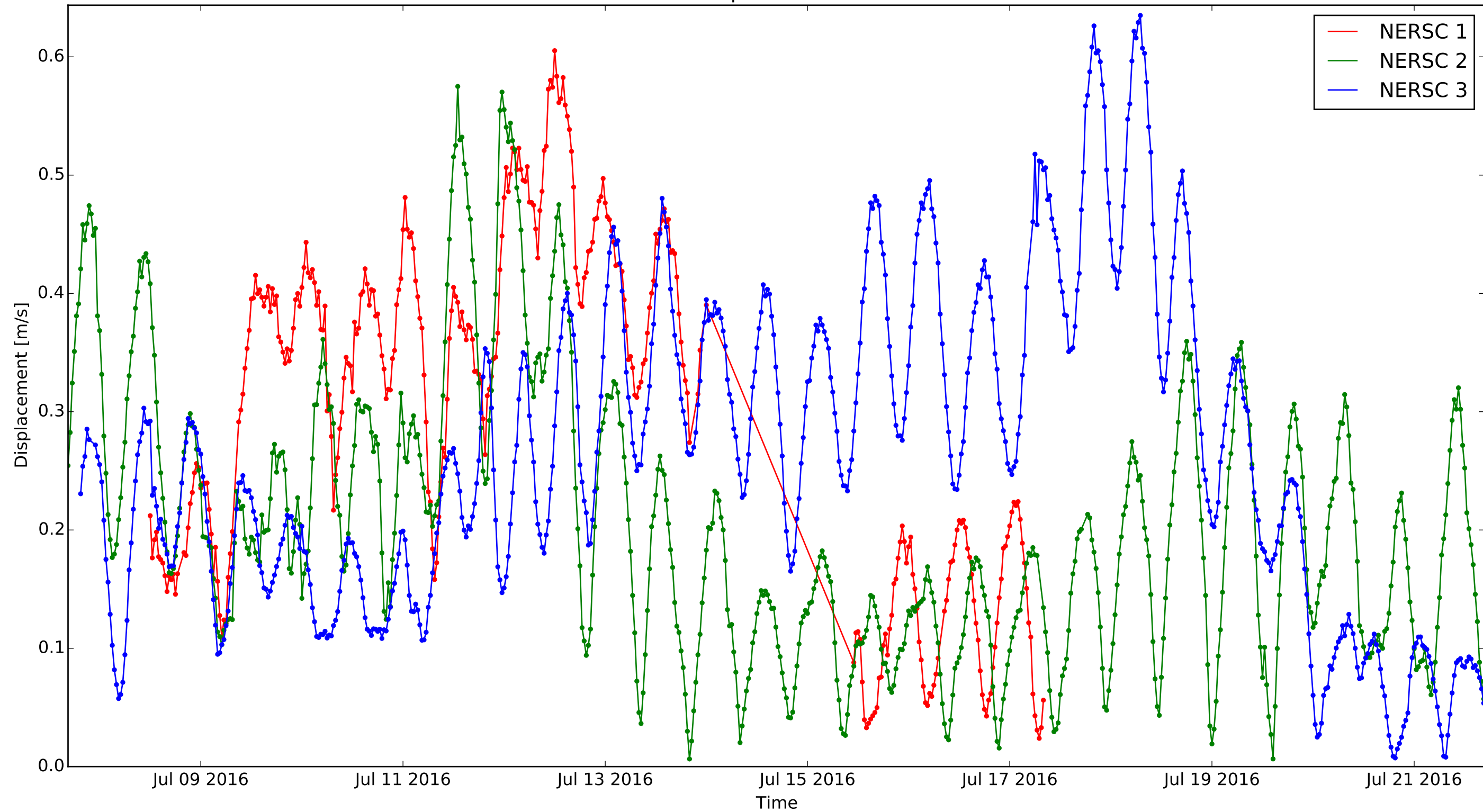

Supplement: Supplementary file 2 — Supplementary material [file mmc2.zip › GPS_tracker_data_python_plots_satellite/GPS_tracker_plots/timeseries_30min_01_zoom01.pdf]

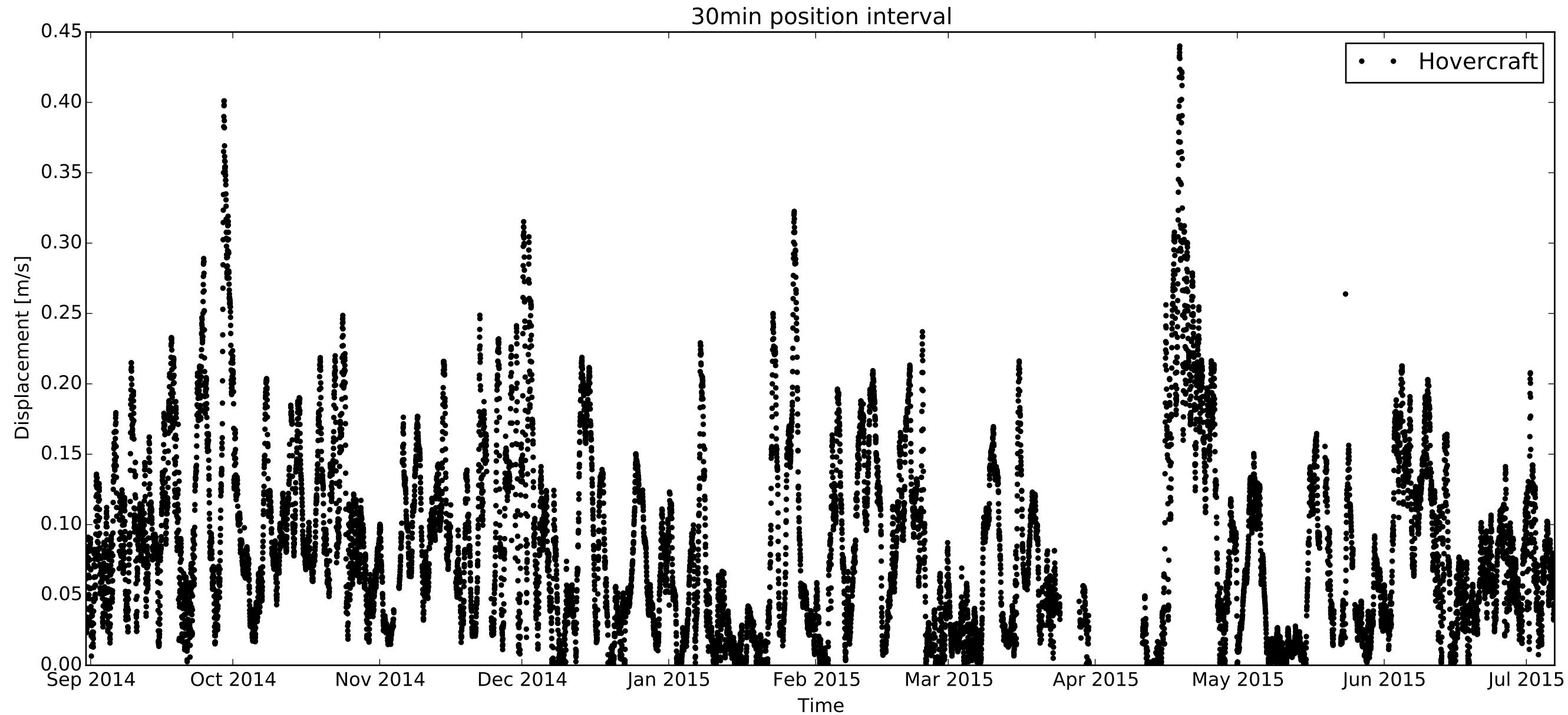

Supplement: Supplementary file 2 — Supplementary material [file mmc2.zip › GPS_tracker_data_python_plots_satellite/GPS_tracker_plots/timeseries_30min_h.pdf]

30min position interval

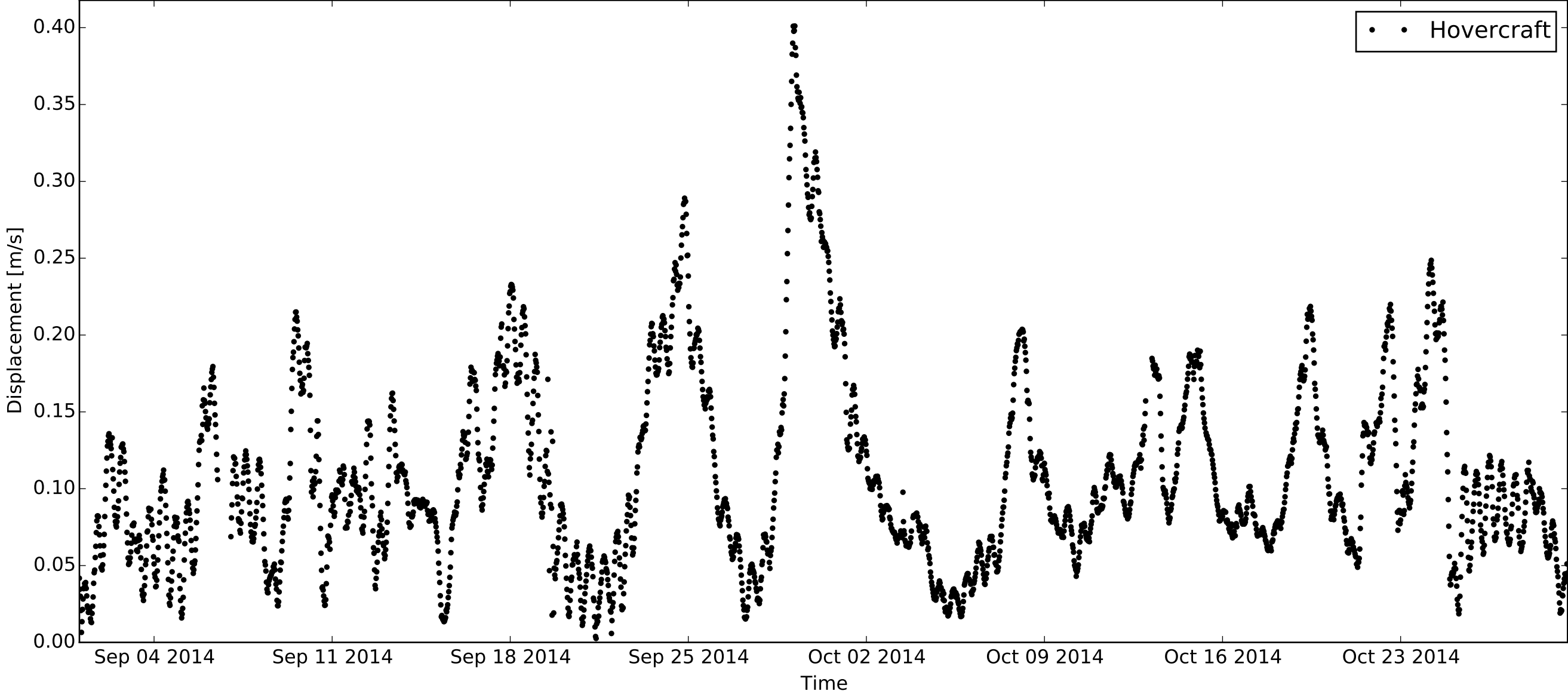

Supplement: Supplementary file 2 — Supplementary material [file mmc2.zip › GPS_tracker_data_python_plots_satellite/GPS_tracker_plots/timeseries_30min_h_zoom01.pdf]

30min position interval

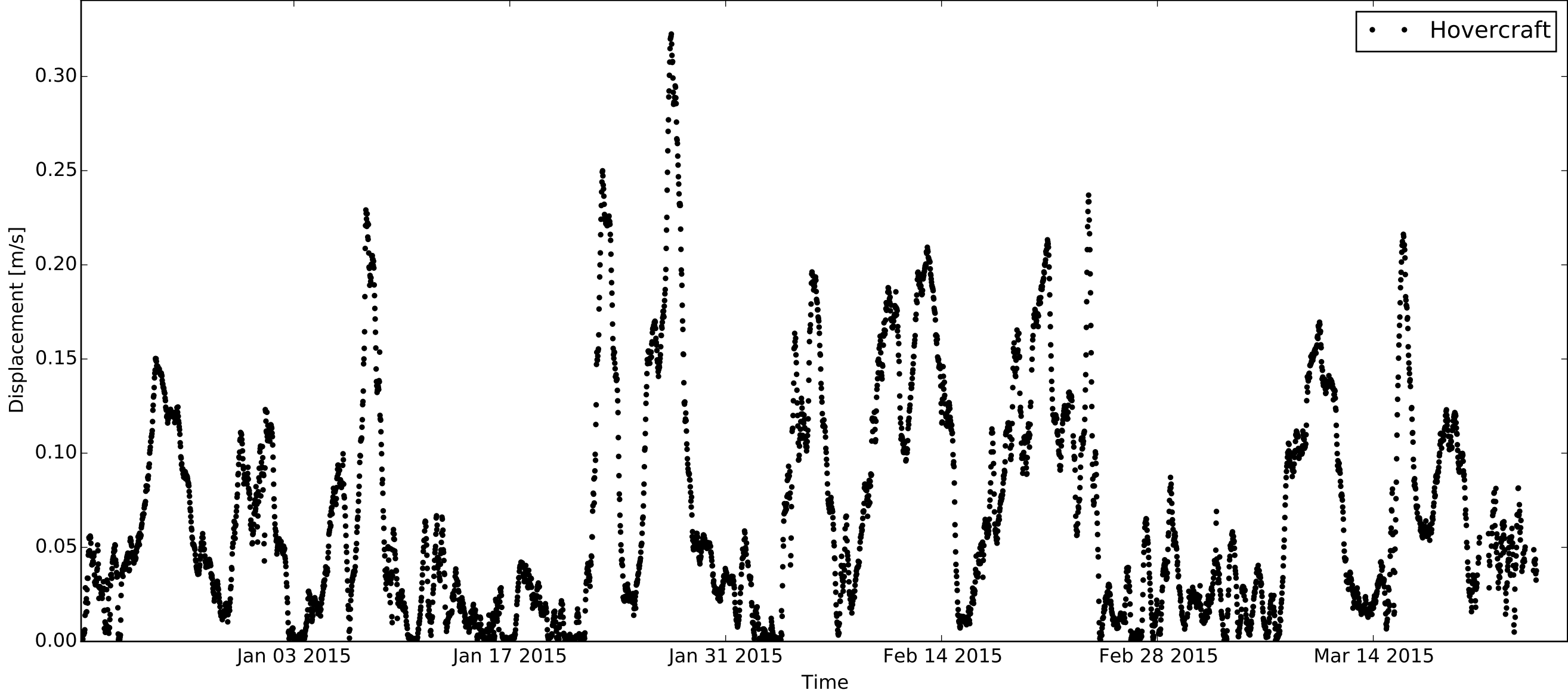

Supplement: Supplementary file 2 — Supplementary material [file mmc2.zip › GPS_tracker_data_python_plots_satellite/GPS_tracker_plots/timeseries_30min_h_zoom02.pdf]

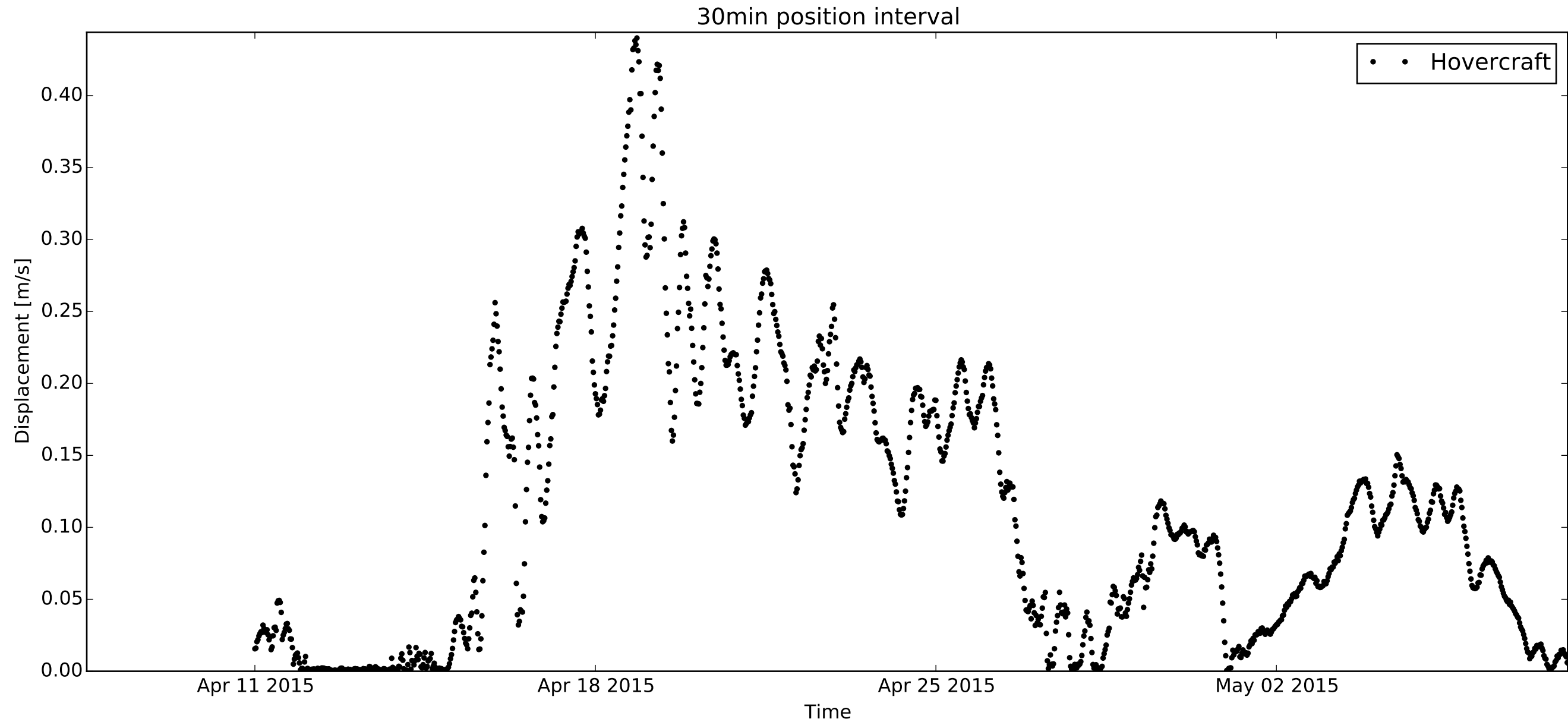

Supplement: Supplementary file 2 — Supplementary material [file mmc2.zip › GPS_tracker_data_python_plots_satellite/GPS_tracker_plots/timeseries_30min_h_zoom03.pdf]

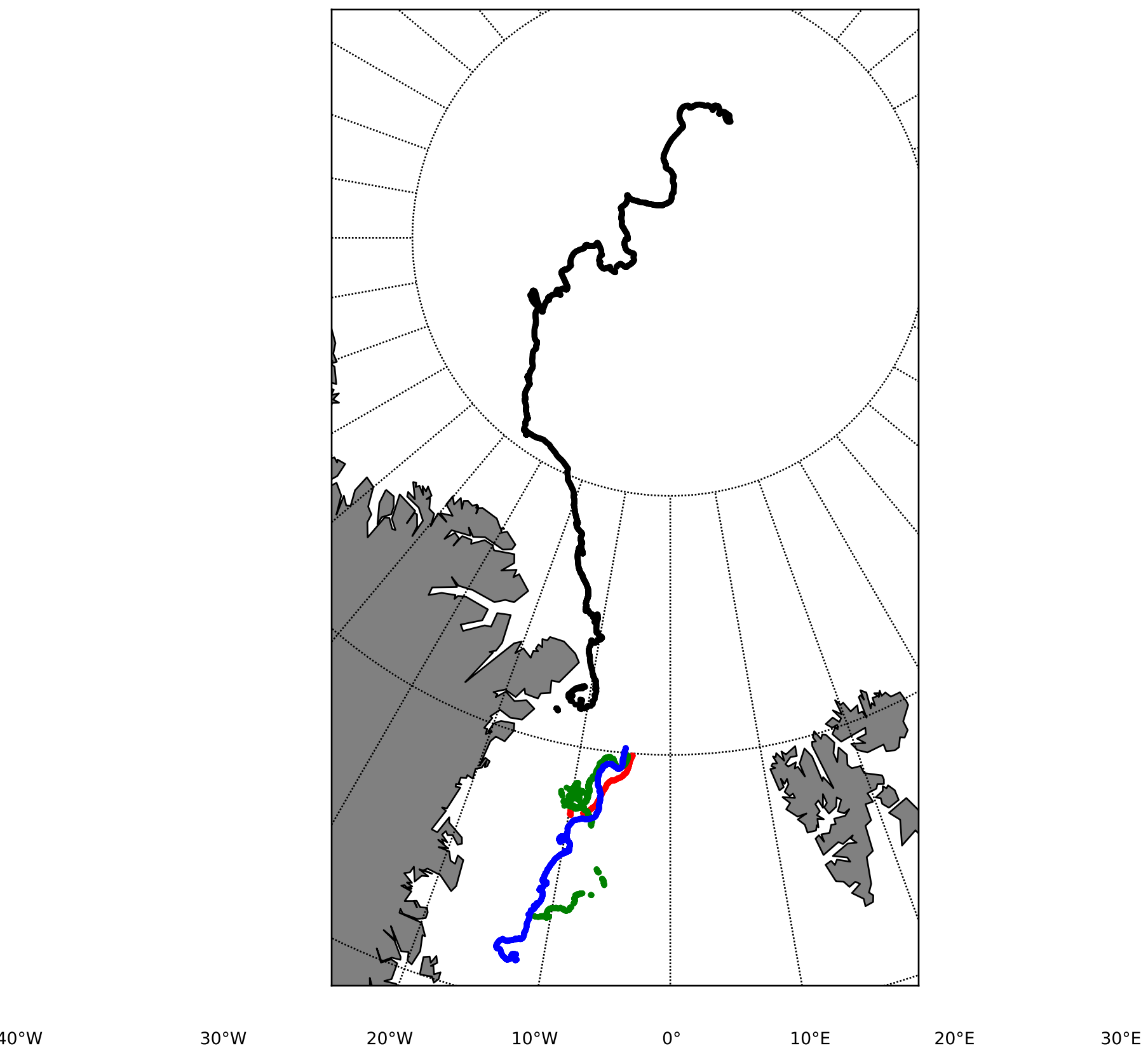

Supplement: Supplementary file 2 — Supplementary material [file mmc2.zip › GPS_tracker_data_python_plots_satellite/GPS_tracker_plots/trajectory_01.pdf]

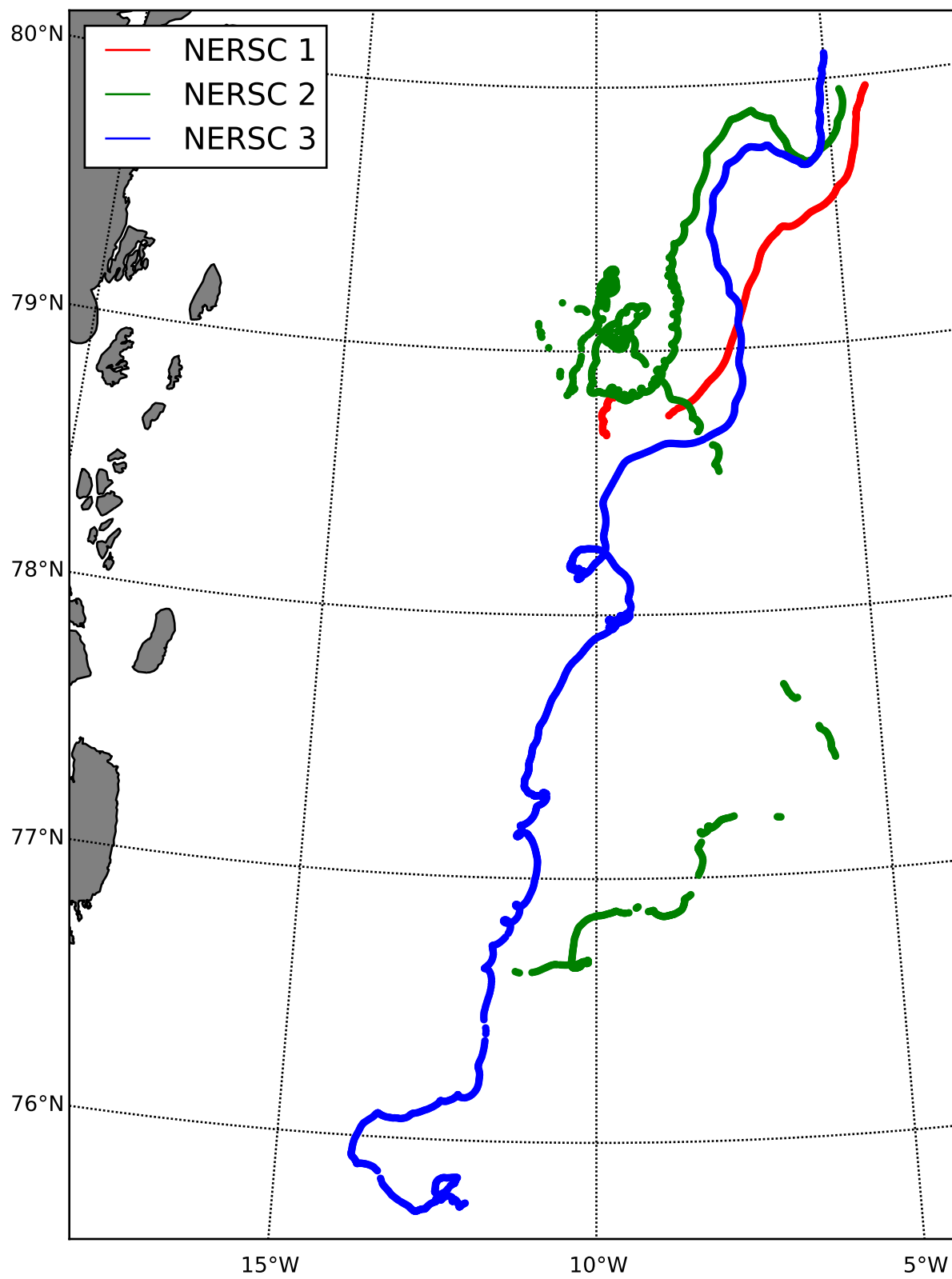

Supplement: Supplementary file 2 — Supplementary material [file mmc2.zip › GPS_tracker_data_python_plots_satellite/GPS_tracker_plots/trajectory_02.pdf]

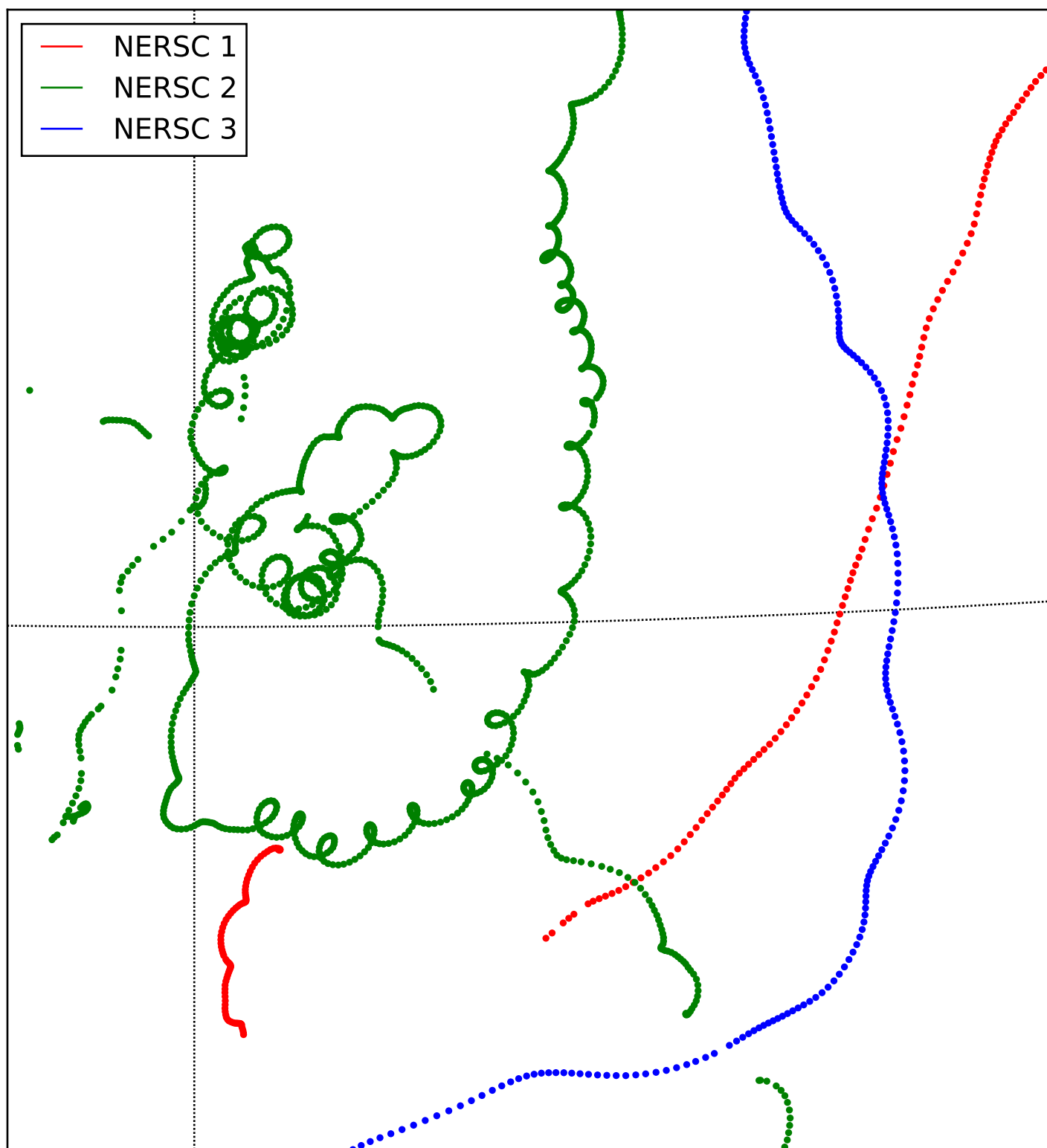

Supplement: Supplementary file 2 — Supplementary material [file mmc2.zip › GPS_tracker_data_python_plots_satellite/GPS_tracker_plots/trajectory_02_zoom01.pdf]

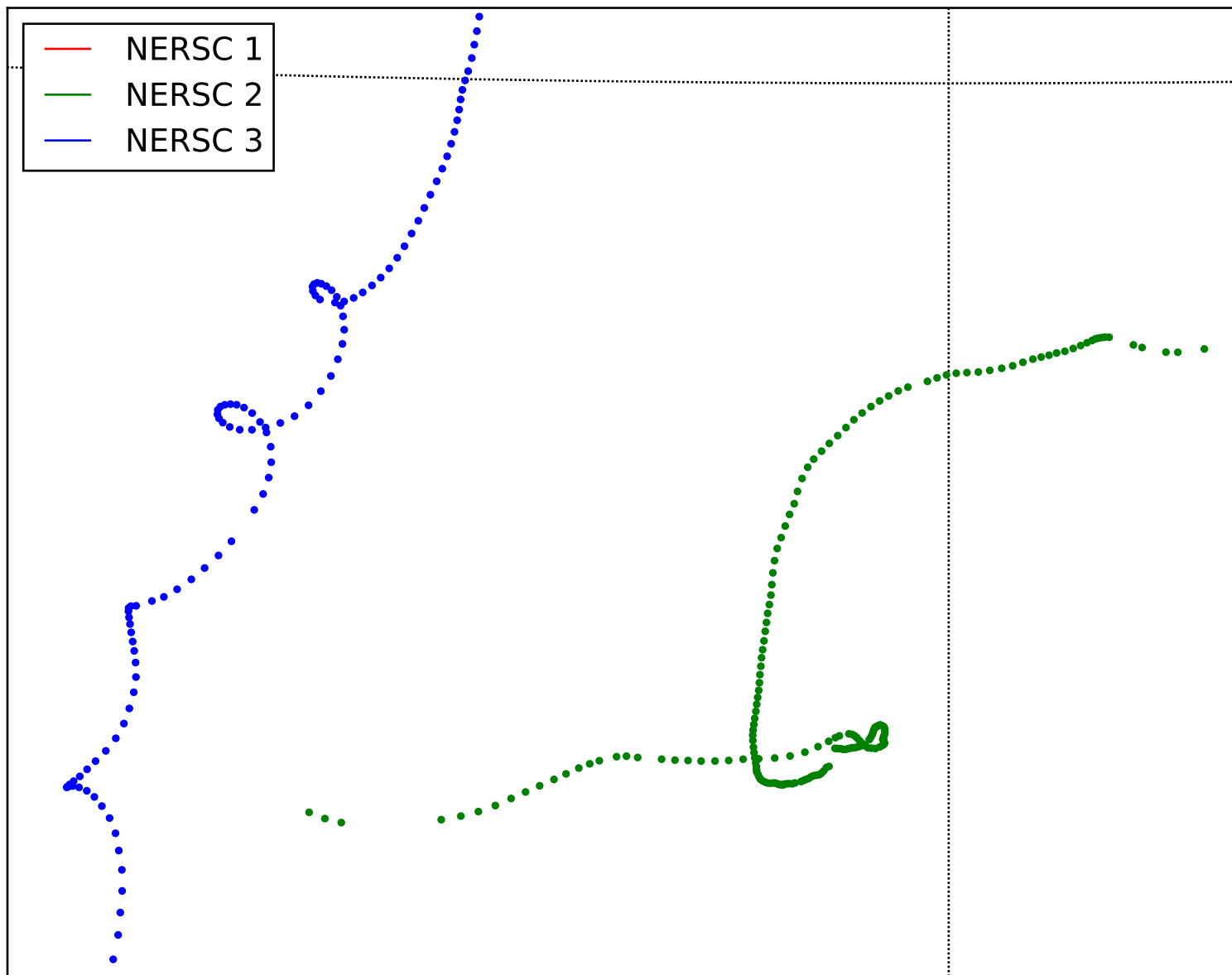

Supplement: Supplementary file 2 — Supplementary material [file mmc2.zip › GPS_tracker_data_python_plots_satellite/GPS_tracker_plots/trajectory_02_zoom02.pdf]

— Hovercraft

80°N

80°N

40°W

30°W

20°W

10°W

0°

10°E

20°E

30°E

40°E

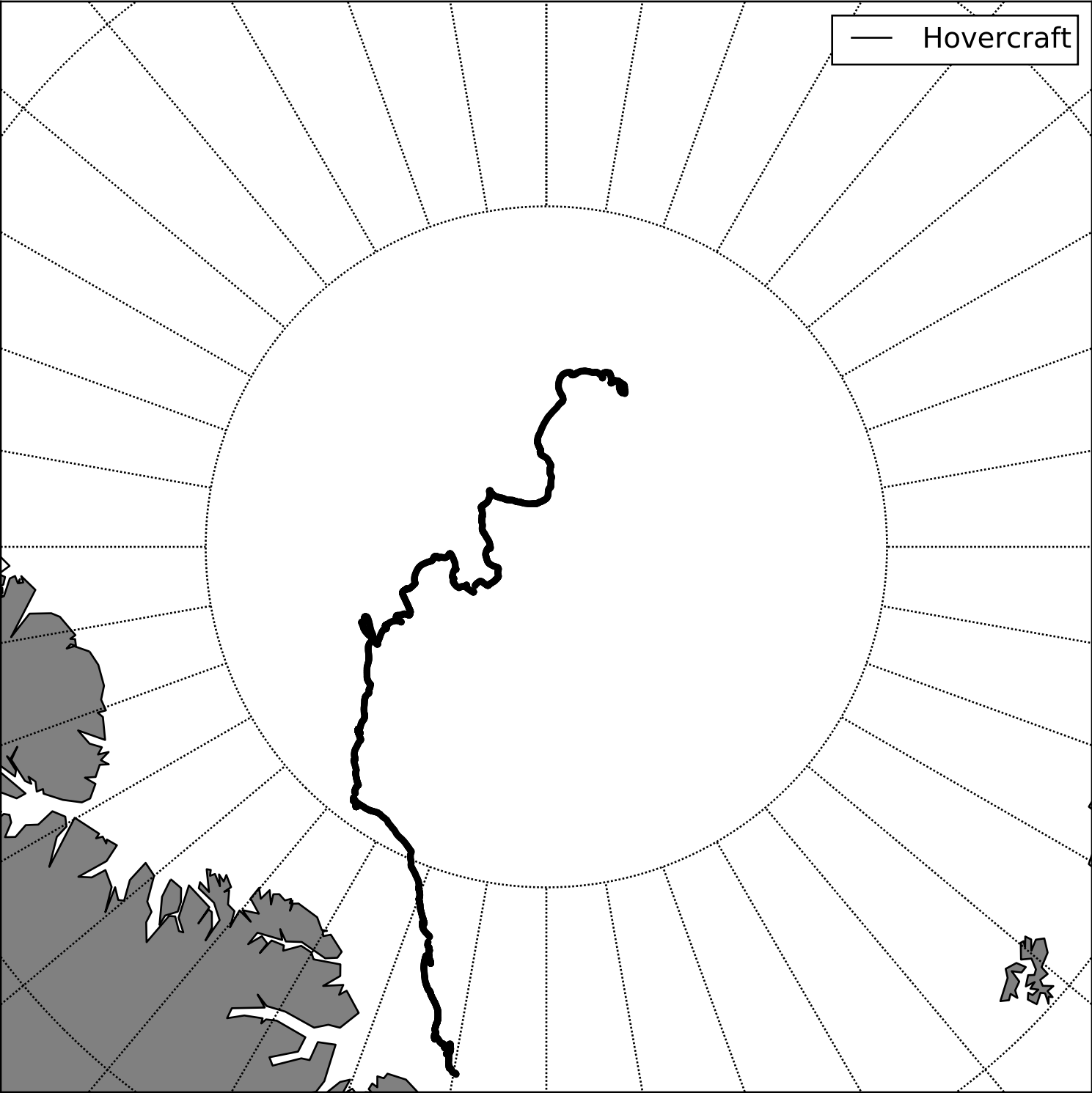

Supplement: Supplementary file 2 — Supplementary material [file mmc2.zip › GPS_tracker_data_python_plots_satellite/GPS_tracker_plots/trajectory_03.pdf]

80°N

40°W

30°W

20°W

10°W

0°

10°E

20°E

30°E

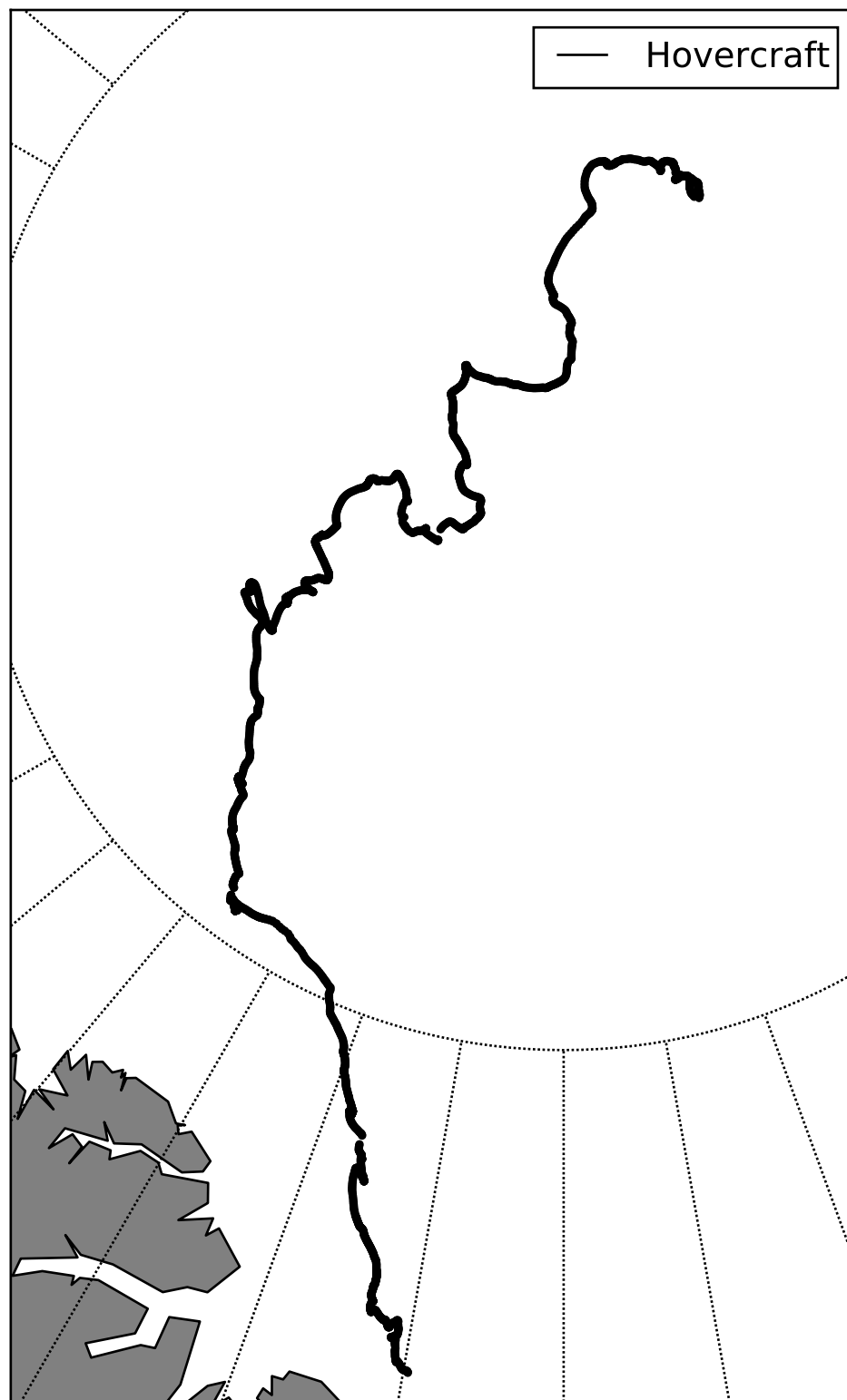

Supplement: Supplementary file 2 — Supplementary material [file mmc2.zip › GPS_tracker_data_python_plots_satellite/GPS_tracker_plots/trajectory_03_zoom01.pdf]

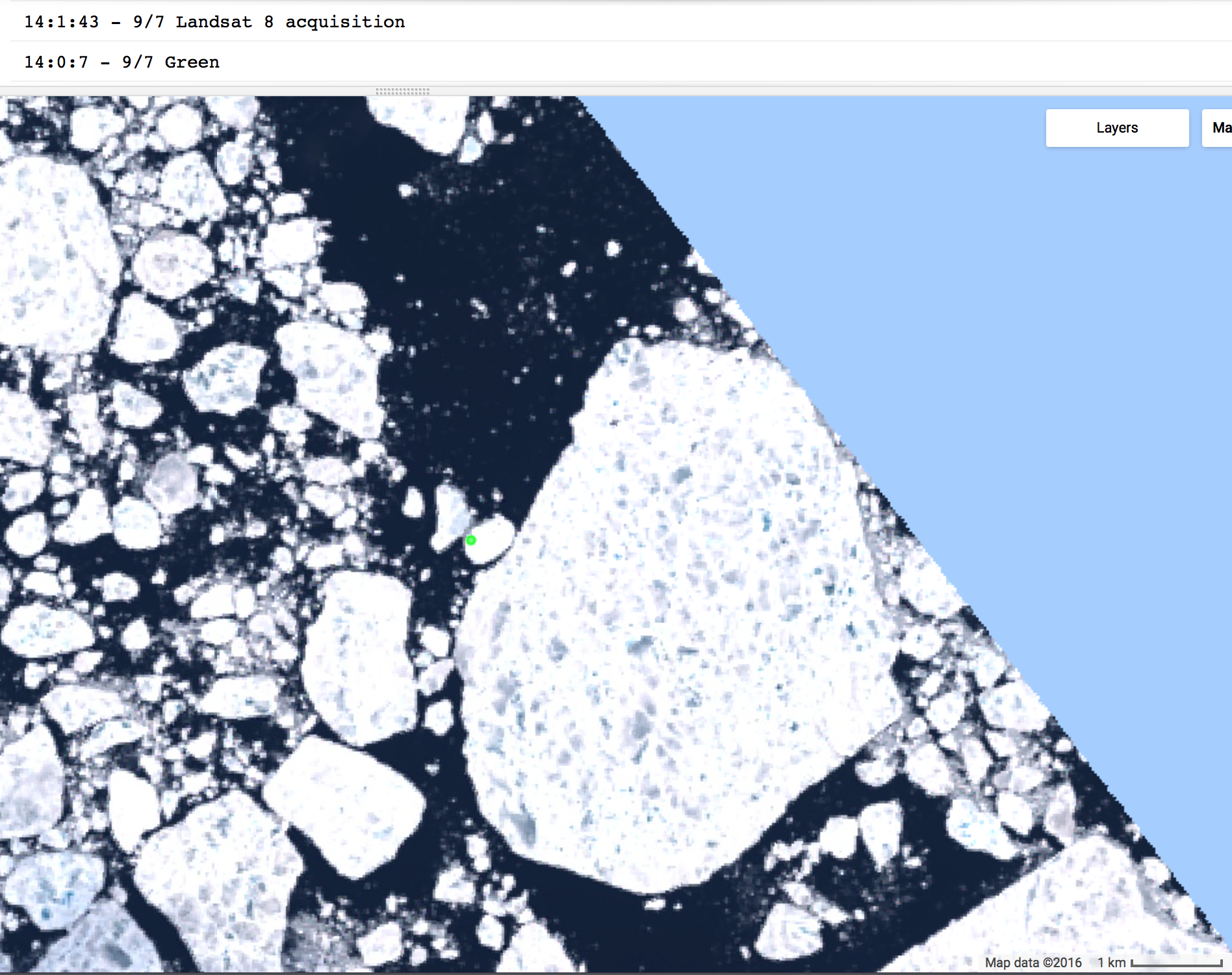

Supplement: Supplementary file 2 — Supplementary material [file mmc2.zip › GPS_tracker_data_python_plots_satellite/GPS_tracker_sat_data/landsat8/L8_20160709_g.png]

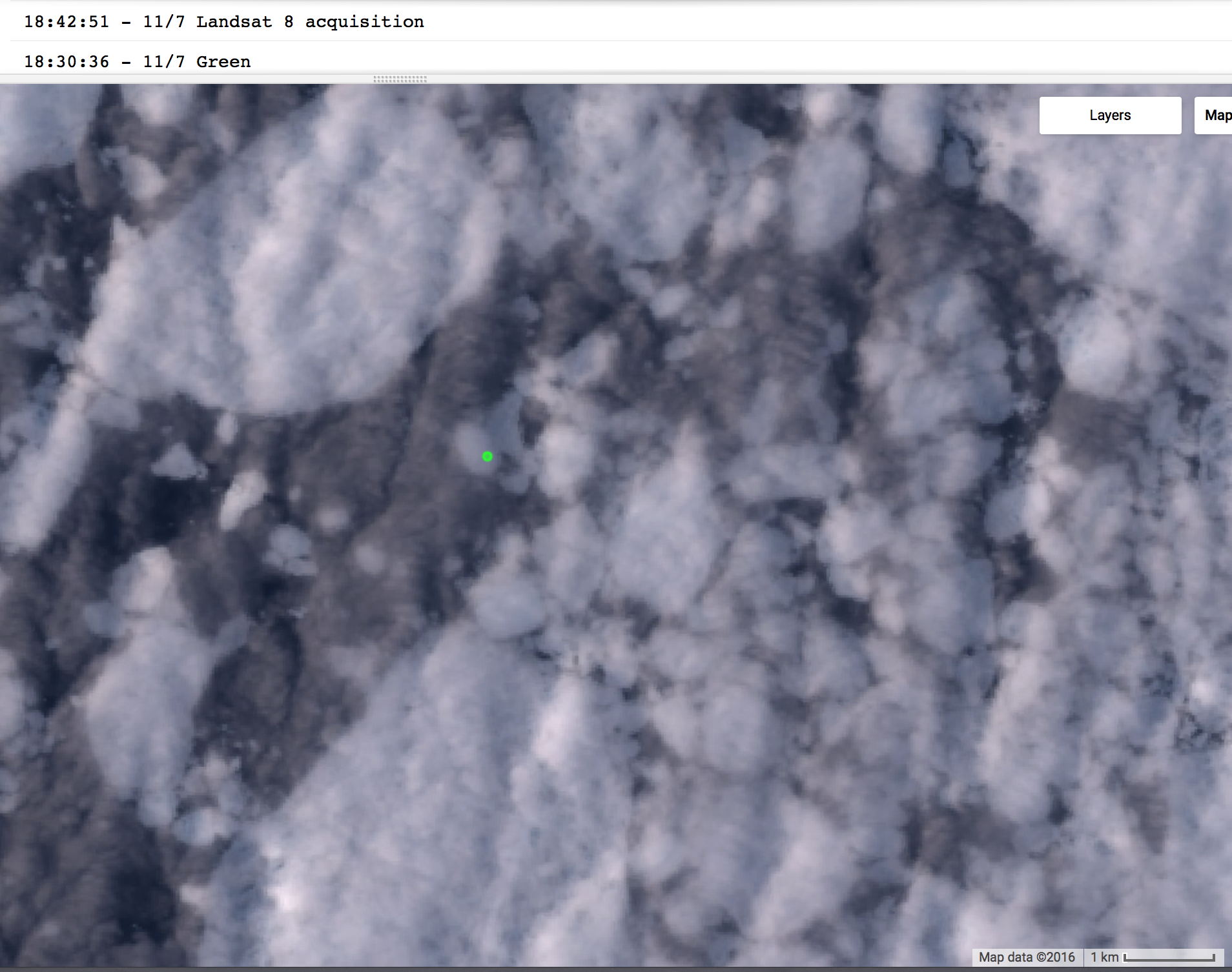

Supplement: Supplementary file 2 — Supplementary material [file mmc2.zip › GPS_tracker_data_python_plots_satellite/GPS_tracker_sat_data/landsat8/L8_20160711_g.png]

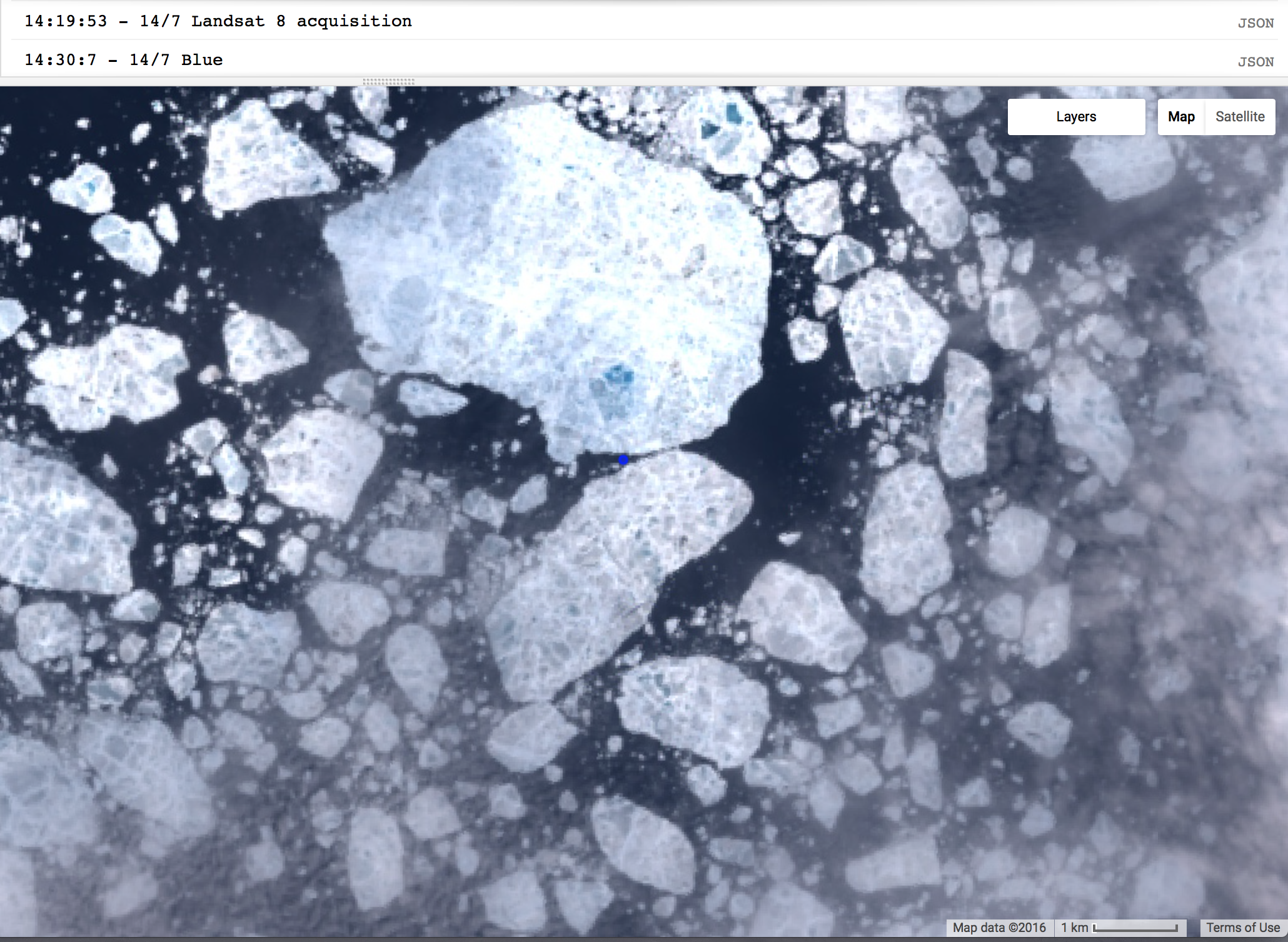

Supplement: Supplementary file 2 — Supplementary material [file mmc2.zip › GPS_tracker_data_python_plots_satellite/GPS_tracker_sat_data/landsat8/L8_20160714_b.png]

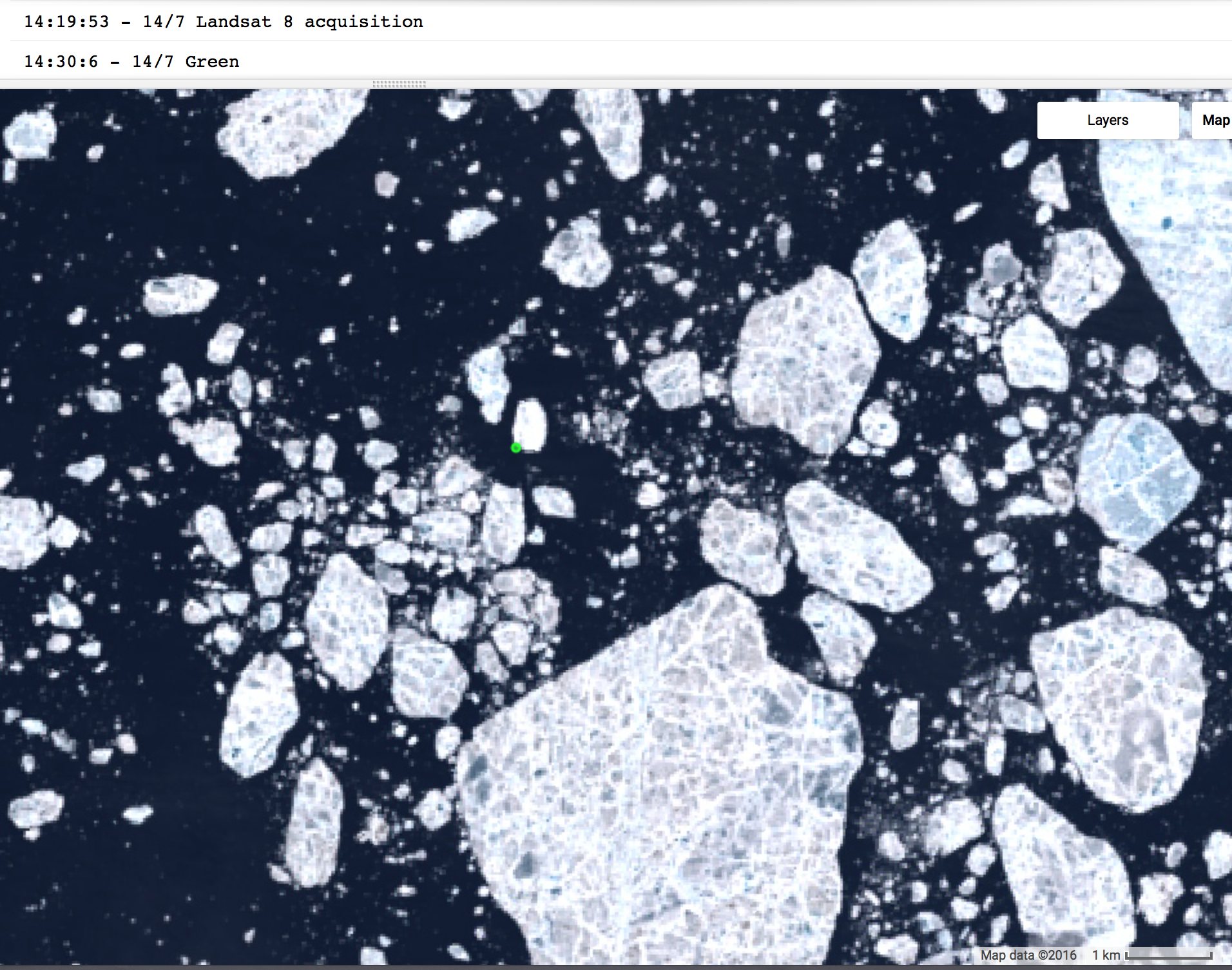

Supplement: Supplementary file 2 — Supplementary material [file mmc2.zip › GPS_tracker_data_python_plots_satellite/GPS_tracker_sat_data/landsat8/L8_20160714_g.png]

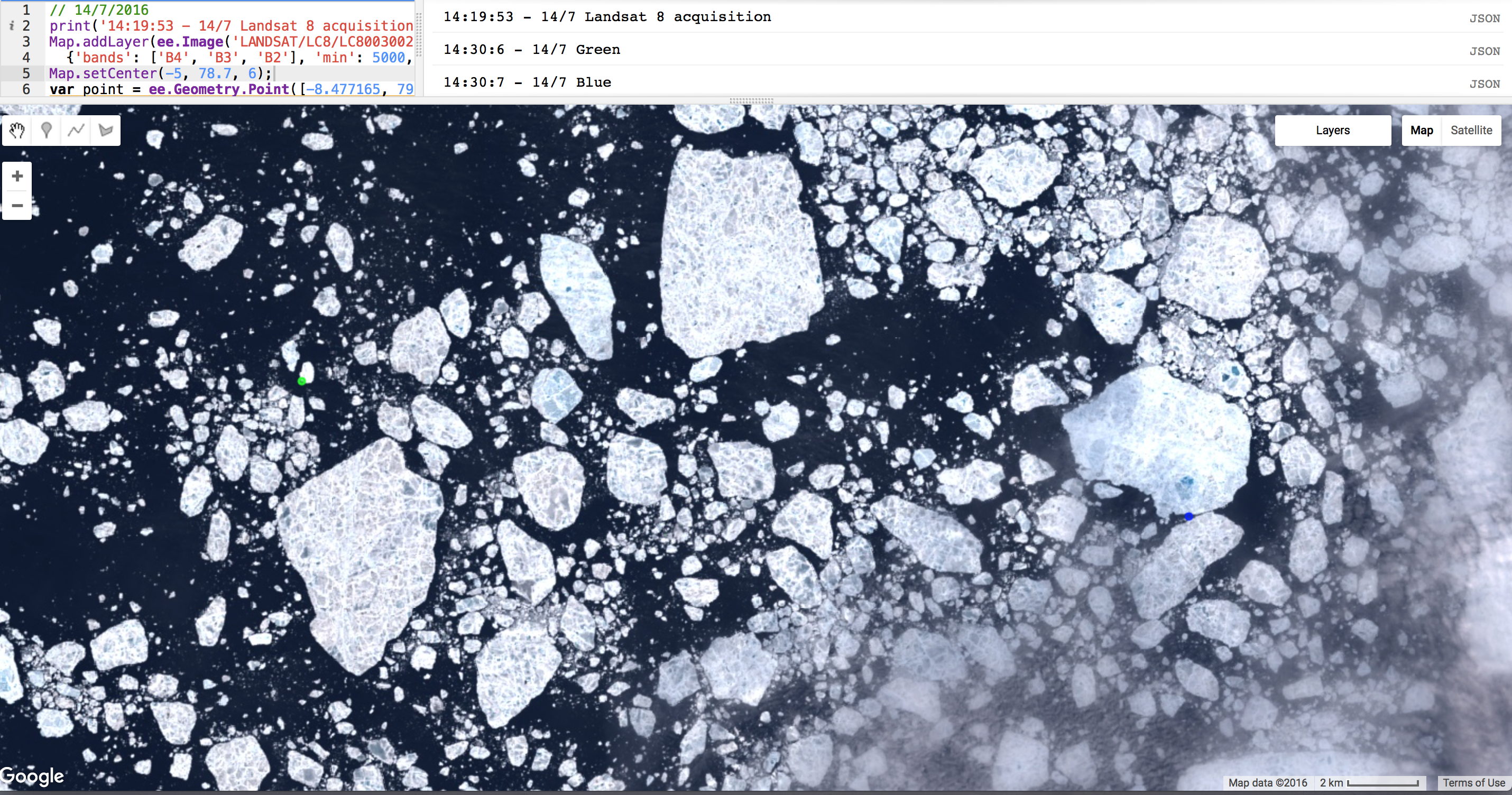

Supplement: Supplementary file 2 — Supplementary material [file mmc2.zip › GPS_tracker_data_python_plots_satellite/GPS_tracker_sat_data/landsat8/L8_20160714_gb.png]

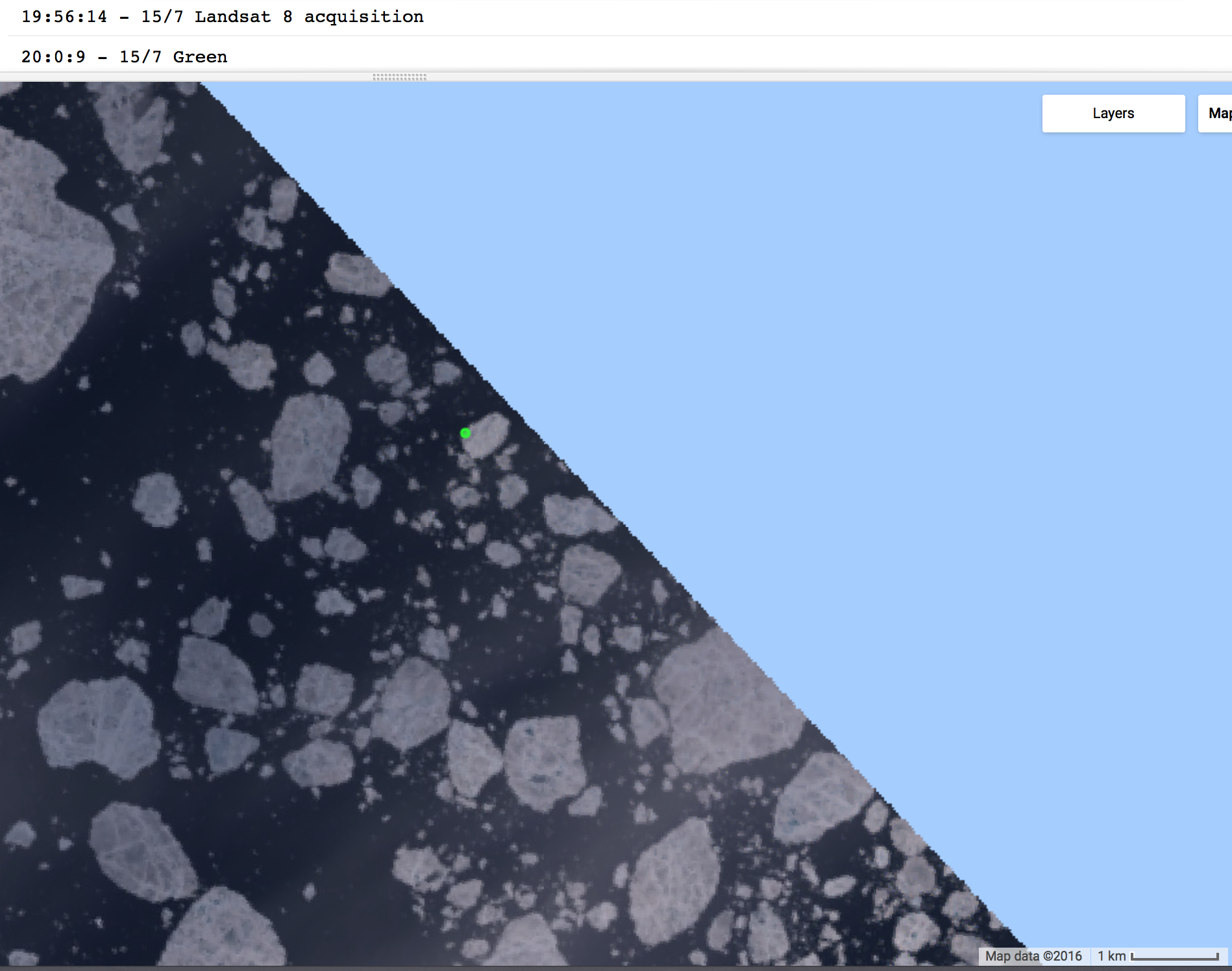

Supplement: Supplementary file 2 — Supplementary material [file mmc2.zip › GPS_tracker_data_python_plots_satellite/GPS_tracker_sat_data/landsat8/L8_20160715_g.png]

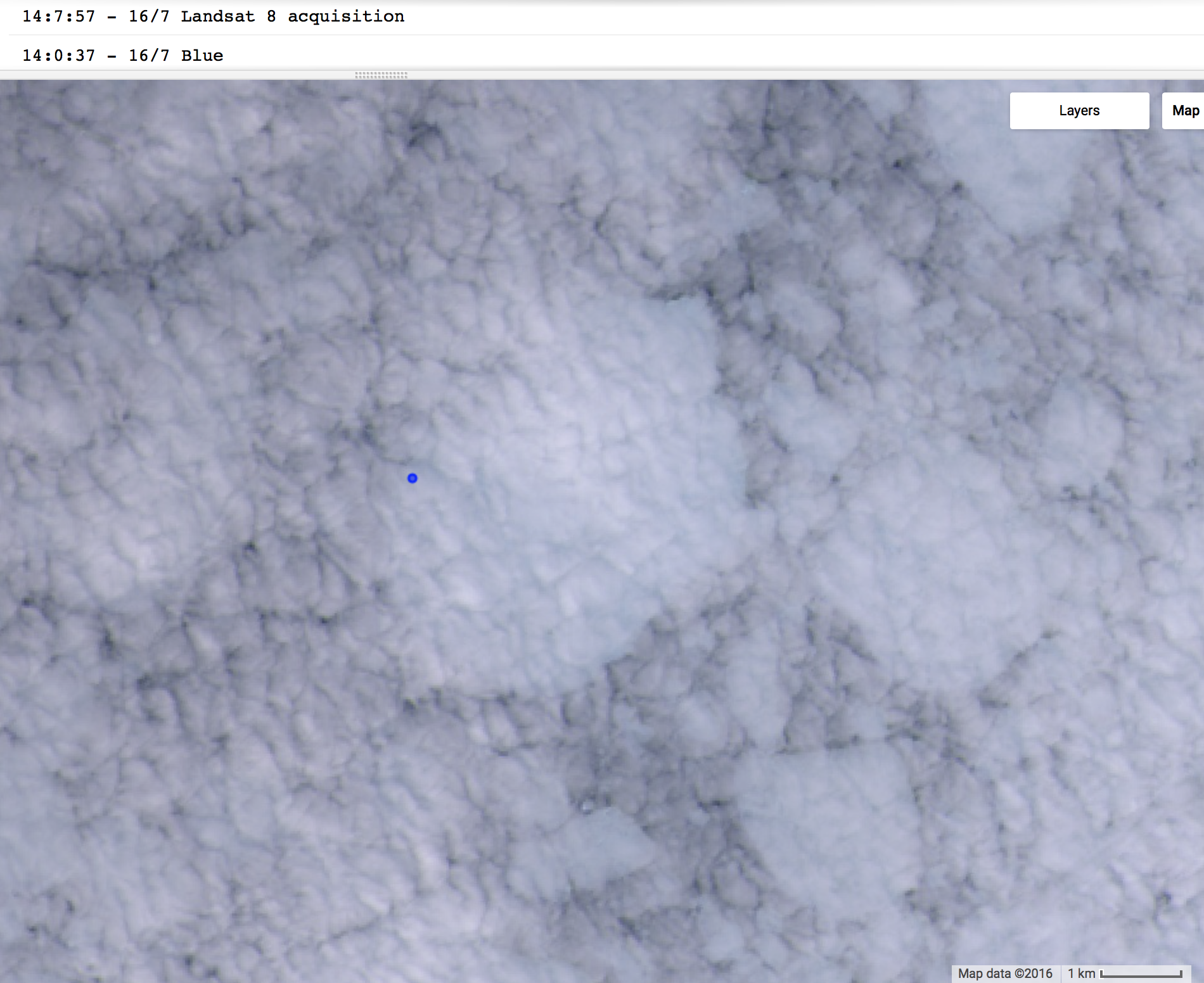

Supplement: Supplementary file 2 — Supplementary material [file mmc2.zip › GPS_tracker_data_python_plots_satellite/GPS_tracker_sat_data/landsat8/L8_20160716_b.png]

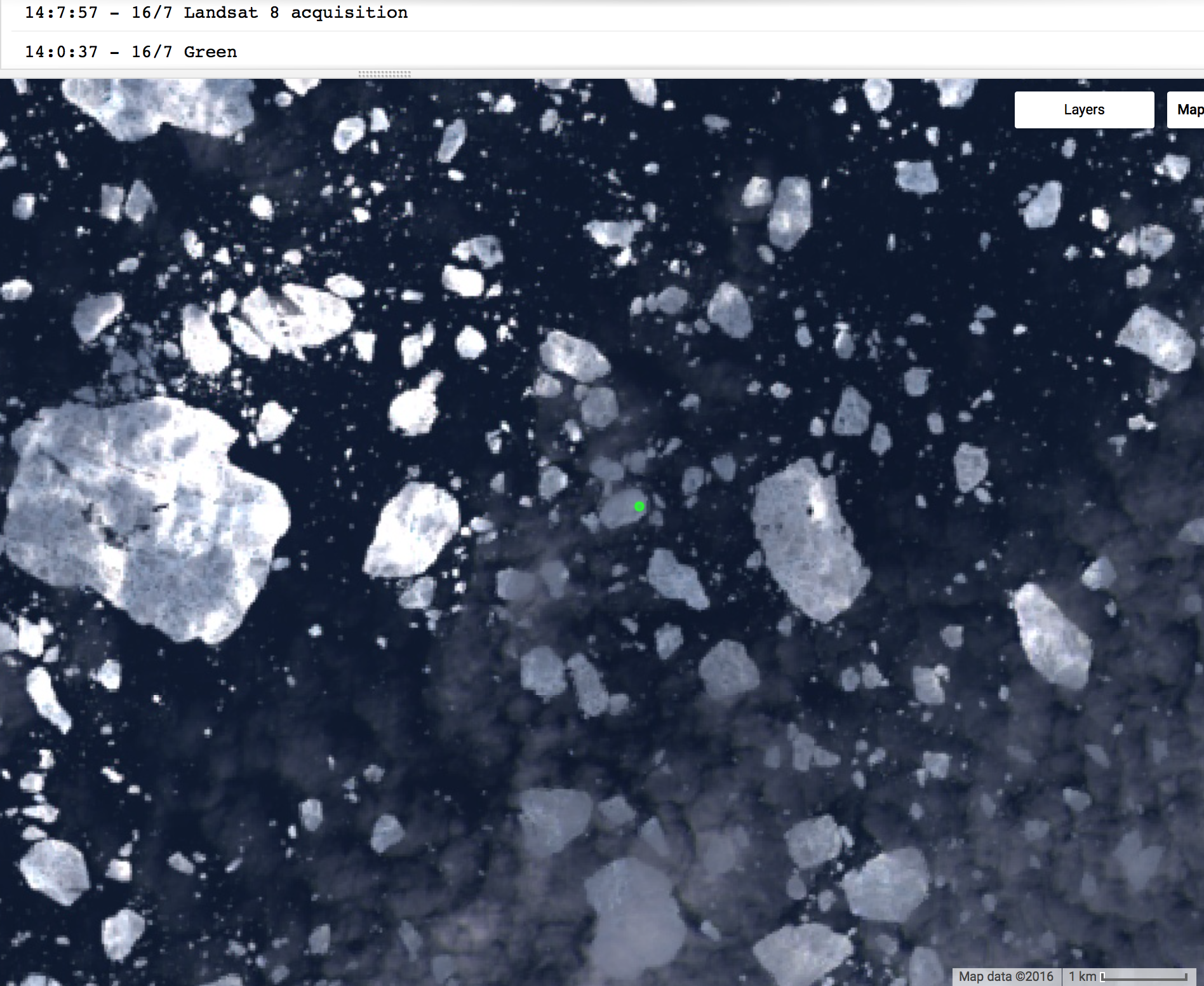

Supplement: Supplementary file 2 — Supplementary material [file mmc2.zip › GPS_tracker_data_python_plots_satellite/GPS_tracker_sat_data/landsat8/L8_20160716_g.png]

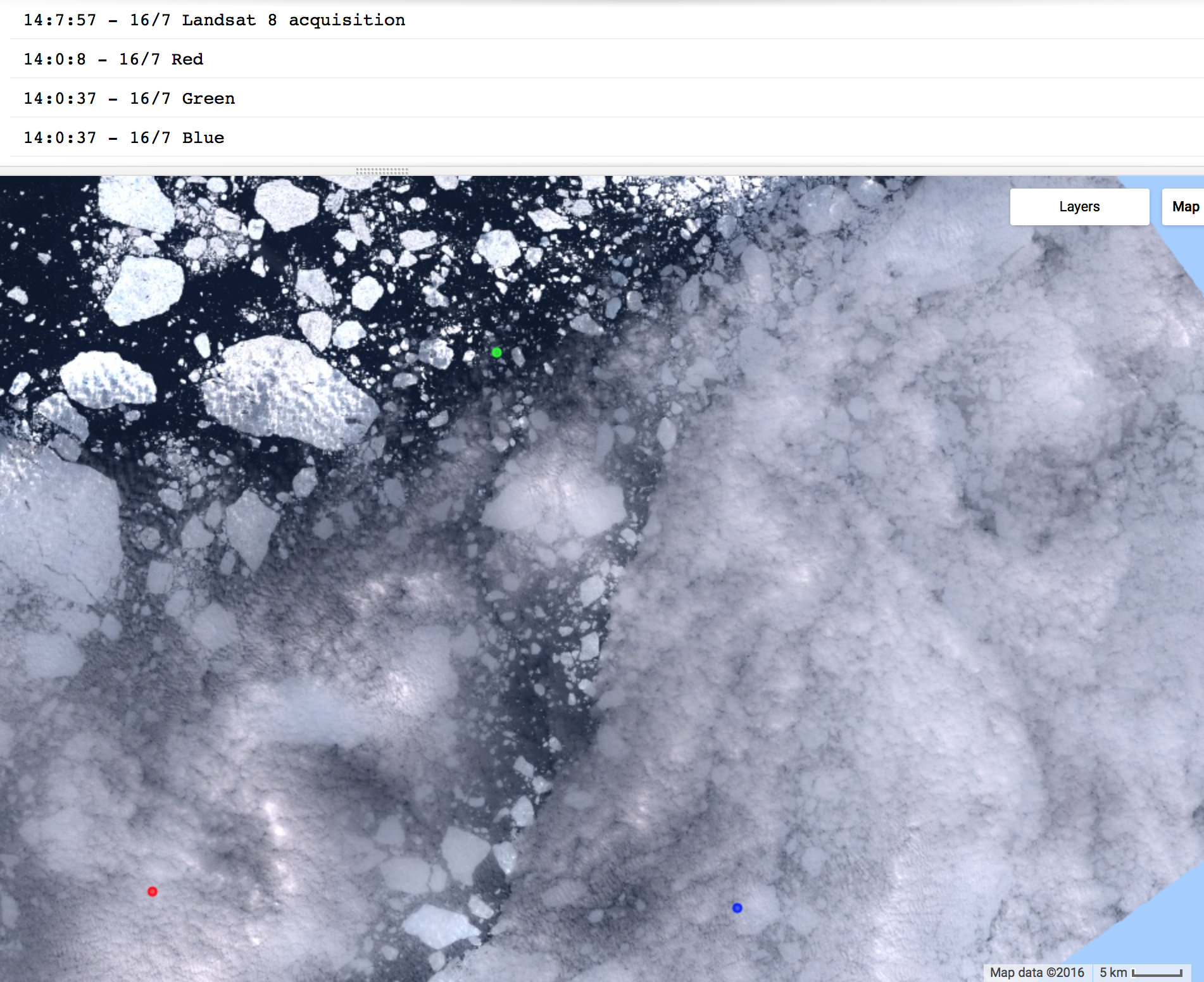

Supplement: Supplementary file 2 — Supplementary material [file mmc2.zip › GPS_tracker_data_python_plots_satellite/GPS_tracker_sat_data/landsat8/L8_20160716_rgb.png]

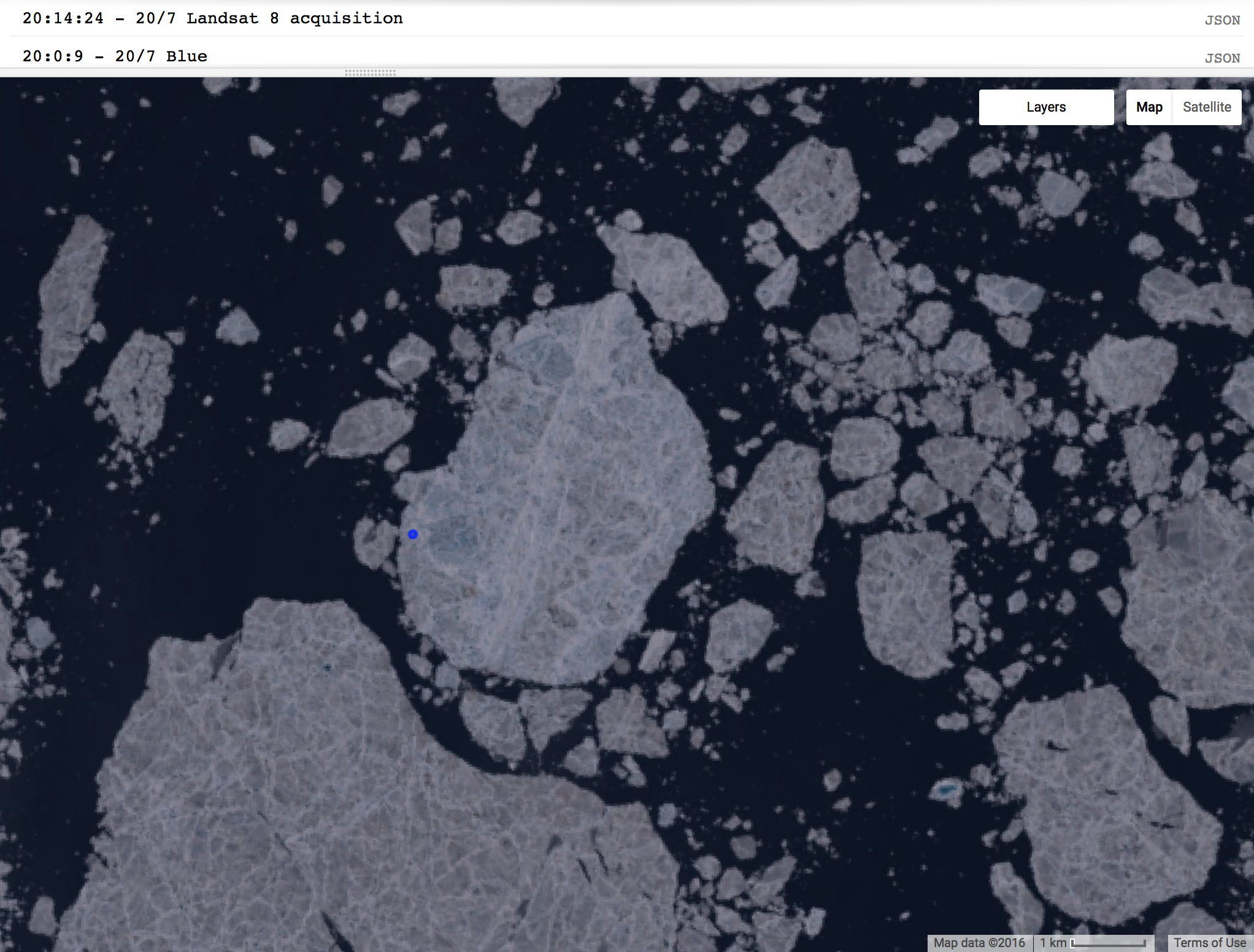

Supplement: Supplementary file 2 — Supplementary material [file mmc2.zip › GPS_tracker_data_python_plots_satellite/GPS_tracker_sat_data/landsat8/L8_20160720_b.png]

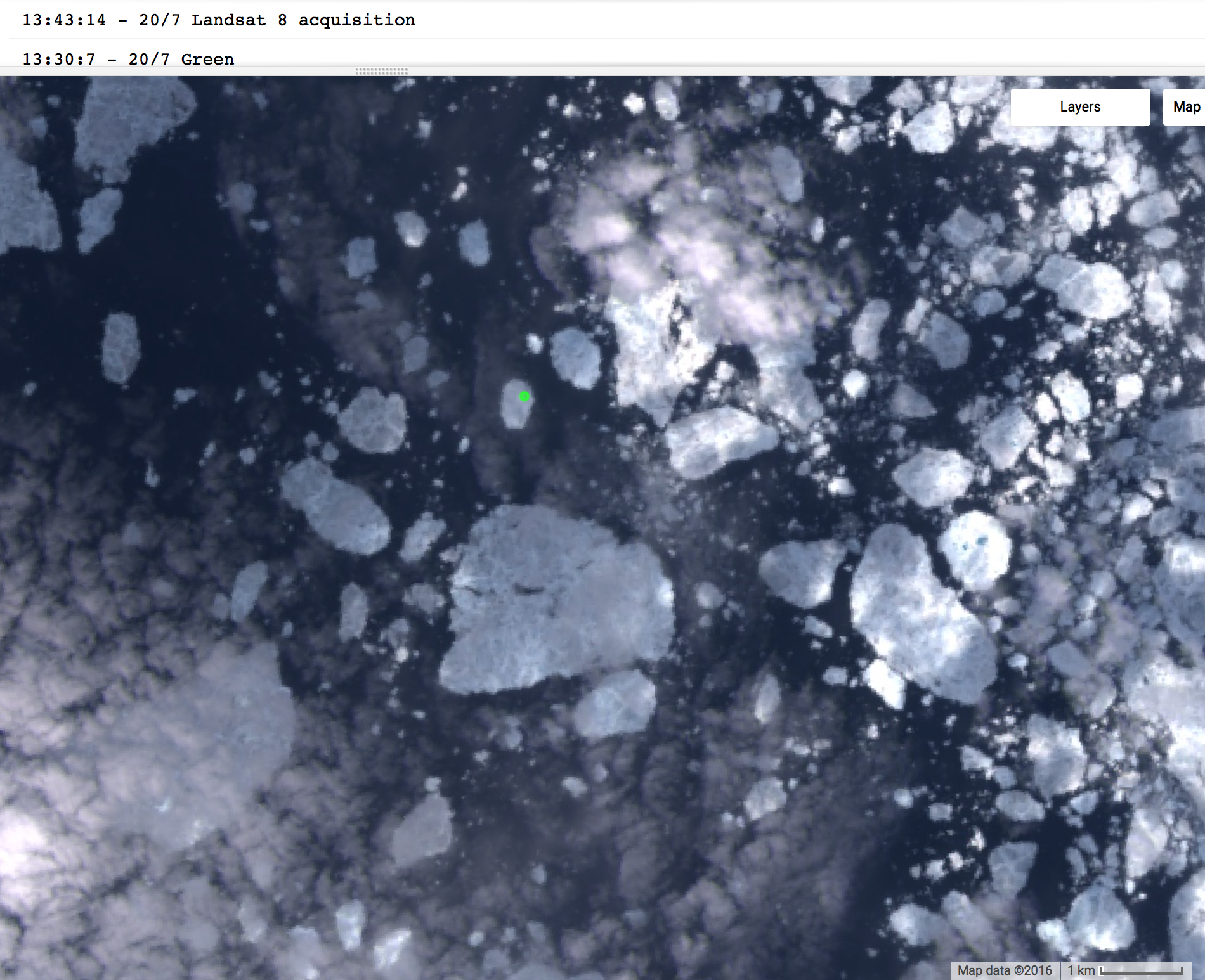

Supplement: Supplementary file 2 — Supplementary material [file mmc2.zip › GPS_tracker_data_python_plots_satellite/GPS_tracker_sat_data/landsat8/L8_20160720_g.png]

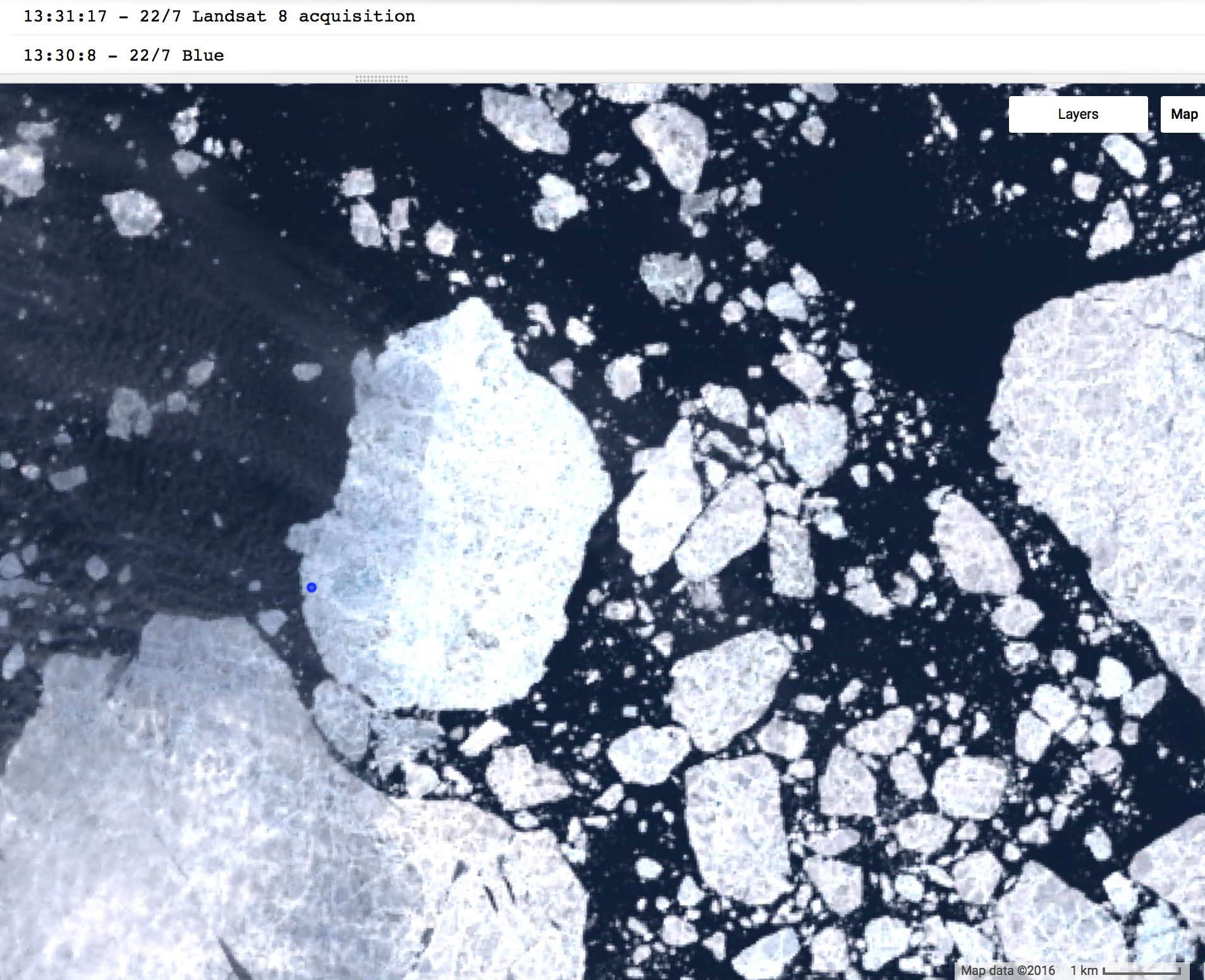

Supplement: Supplementary file 2 — Supplementary material [file mmc2.zip › GPS_tracker_data_python_plots_satellite/GPS_tracker_sat_data/landsat8/L8_20160722_b.png]

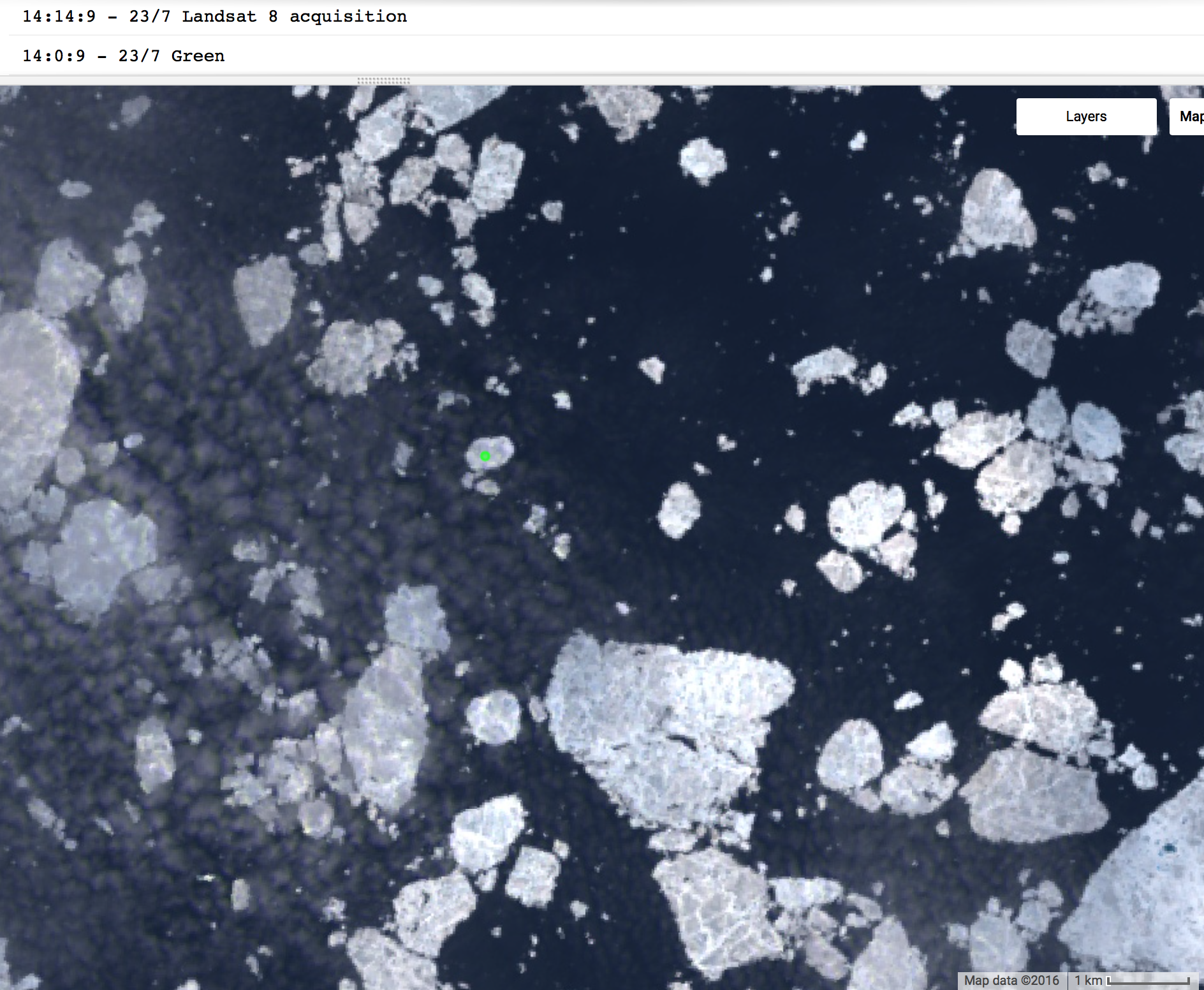

Supplement: Supplementary file 2 — Supplementary material [file mmc2.zip › GPS_tracker_data_python_plots_satellite/GPS_tracker_sat_data/landsat8/L8_20160723_g.png]

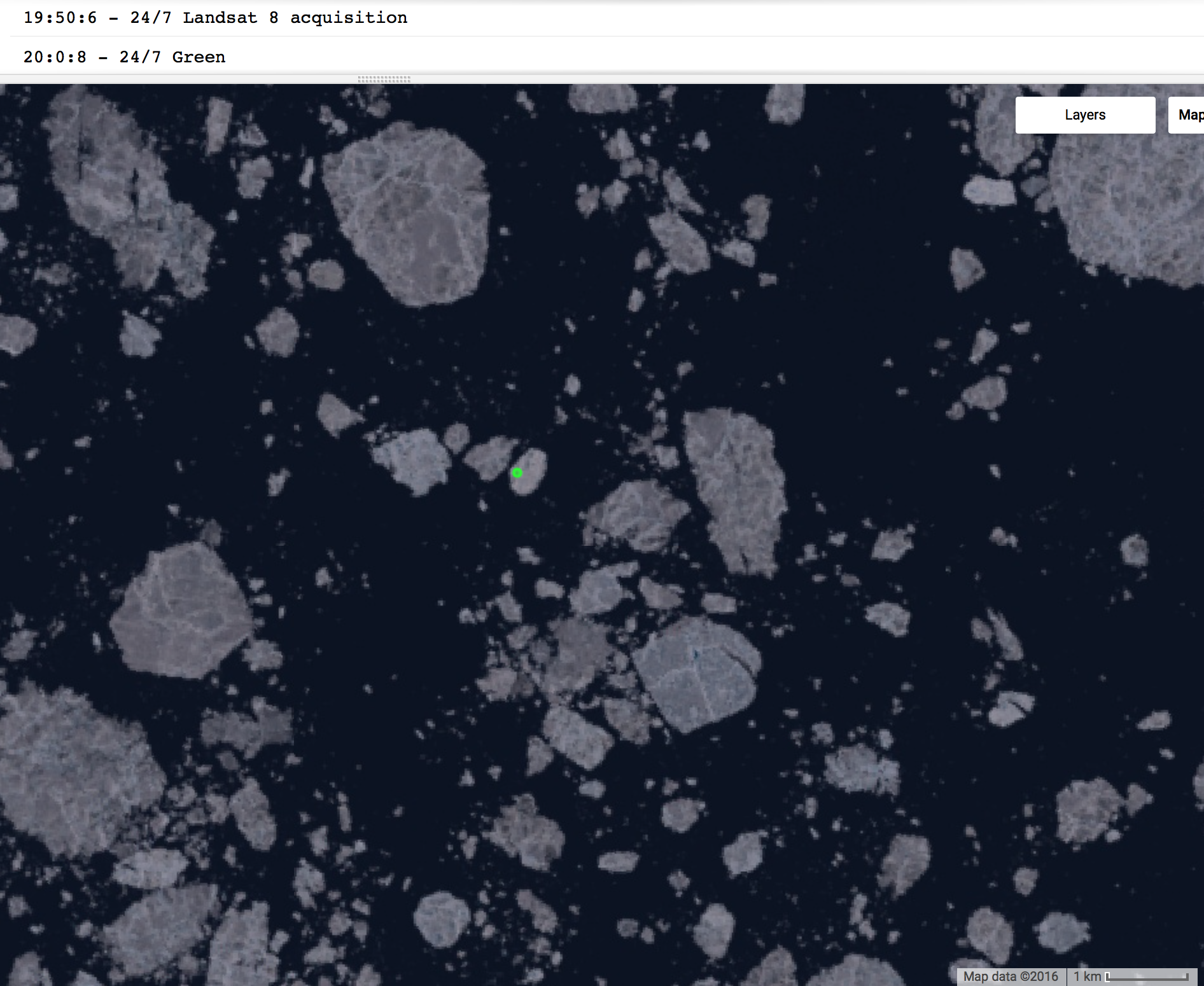

Supplement: Supplementary file 2 — Supplementary material [file mmc2.zip › GPS_tracker_data_python_plots_satellite/GPS_tracker_sat_data/landsat8/L8_20160724_g.png]

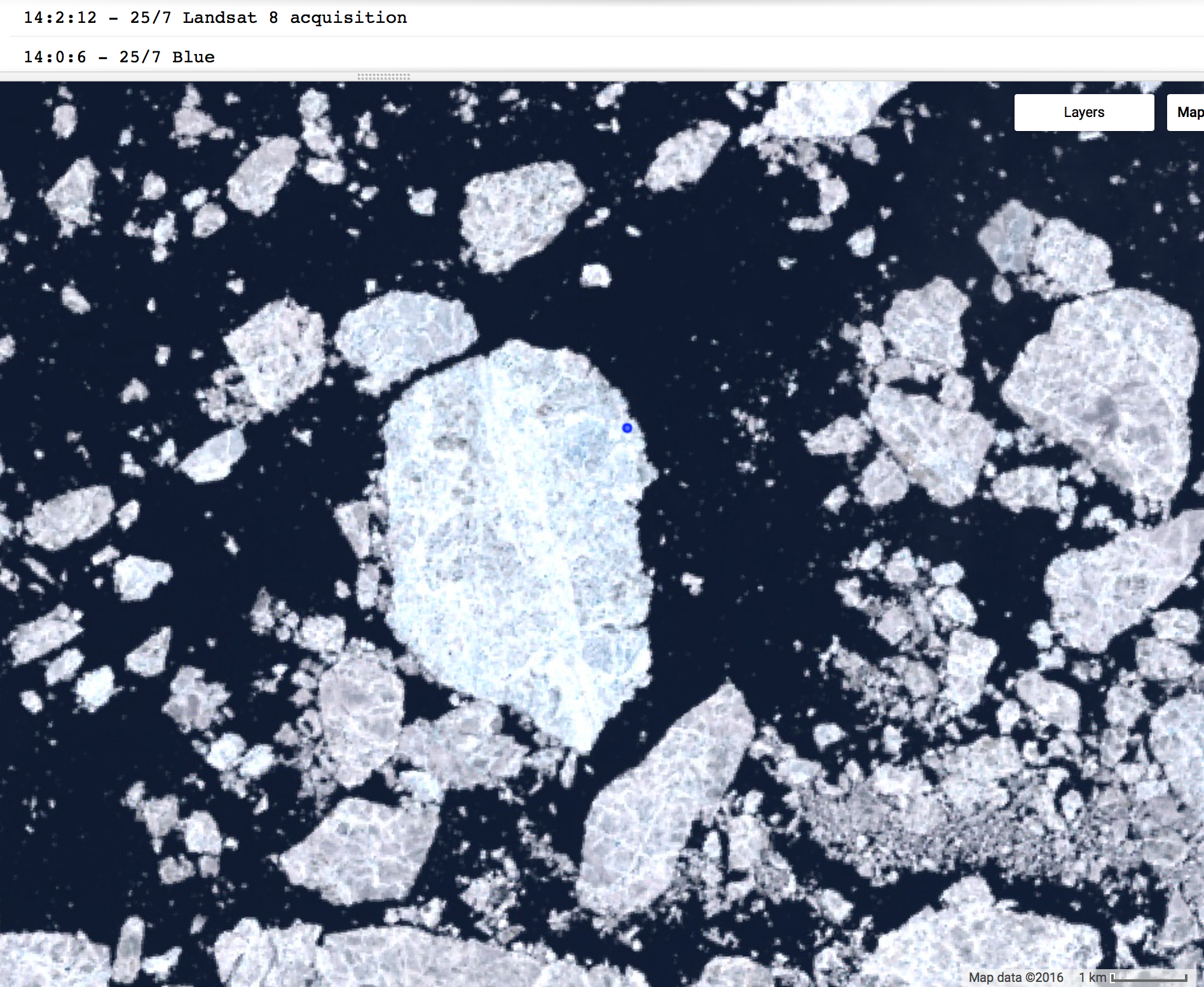

Supplement: Supplementary file 2 — Supplementary material [file mmc2.zip › GPS_tracker_data_python_plots_satellite/GPS_tracker_sat_data/landsat8/L8_20160725_b.png]

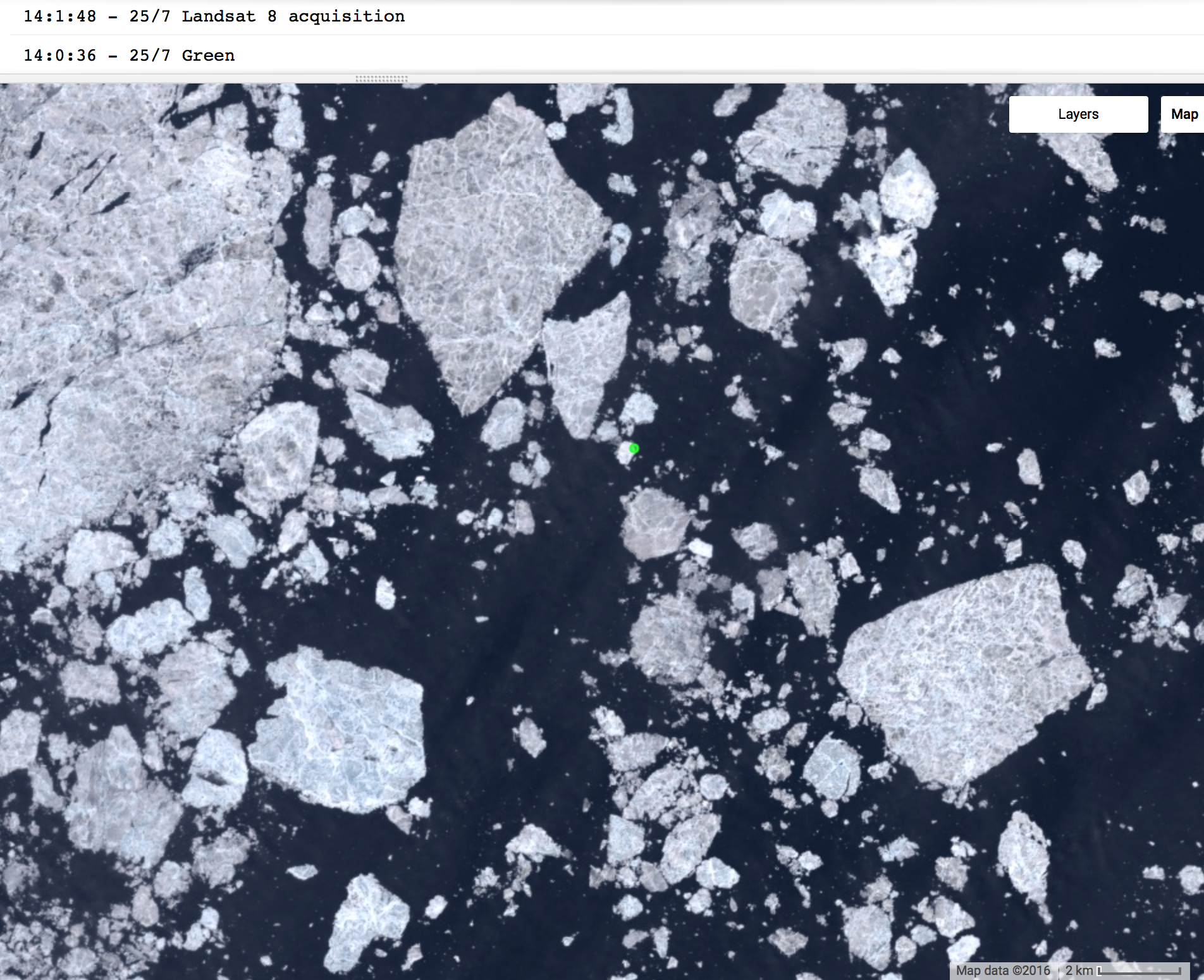

Supplement: Supplementary file 2 — Supplementary material [file mmc2.zip › GPS_tracker_data_python_plots_satellite/GPS_tracker_sat_data/landsat8/L8_20160725_g.png]

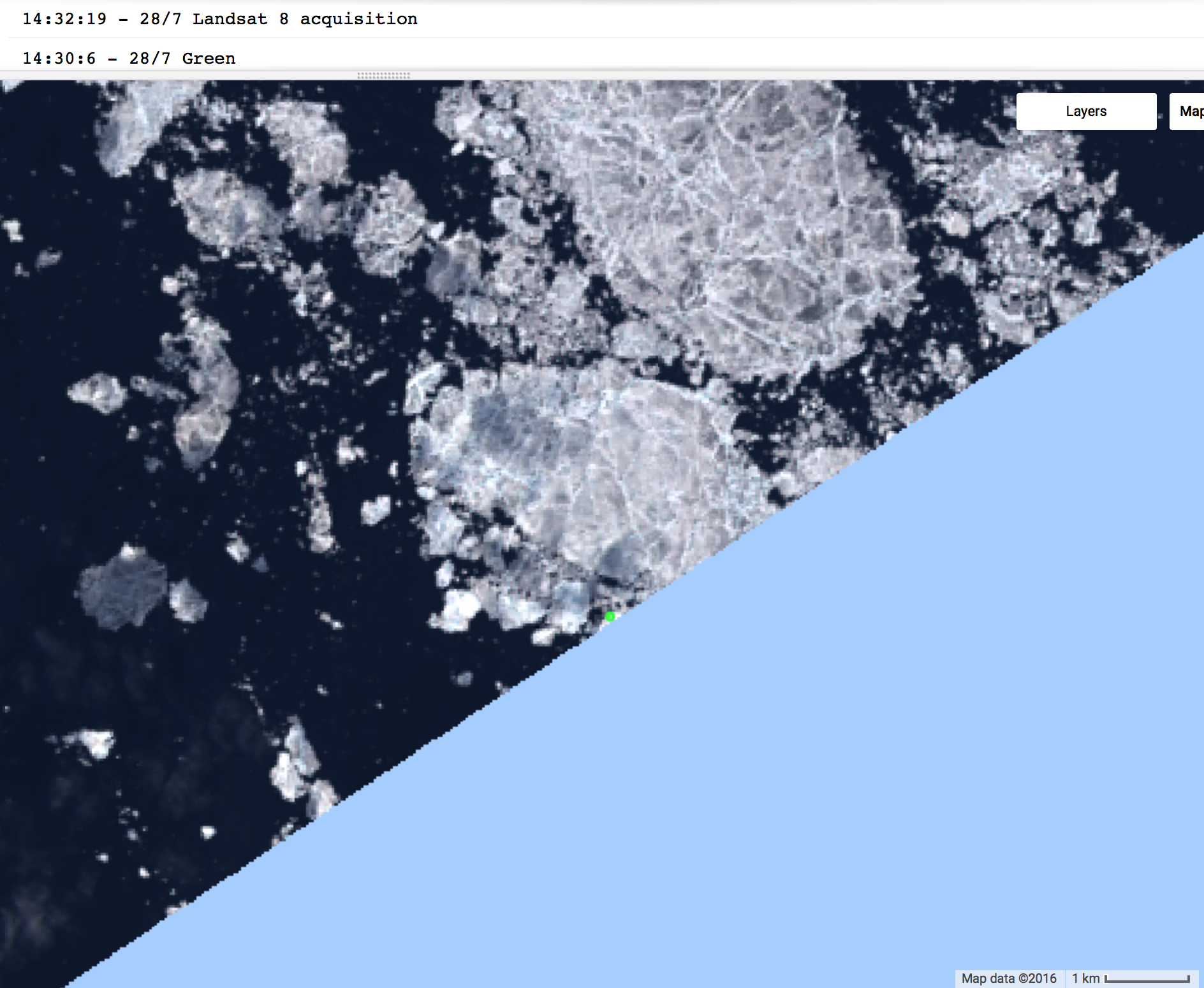

Supplement: Supplementary file 2 — Supplementary material [file mmc2.zip › GPS_tracker_data_python_plots_satellite/GPS_tracker_sat_data/landsat8/L8_20160728_g.png]

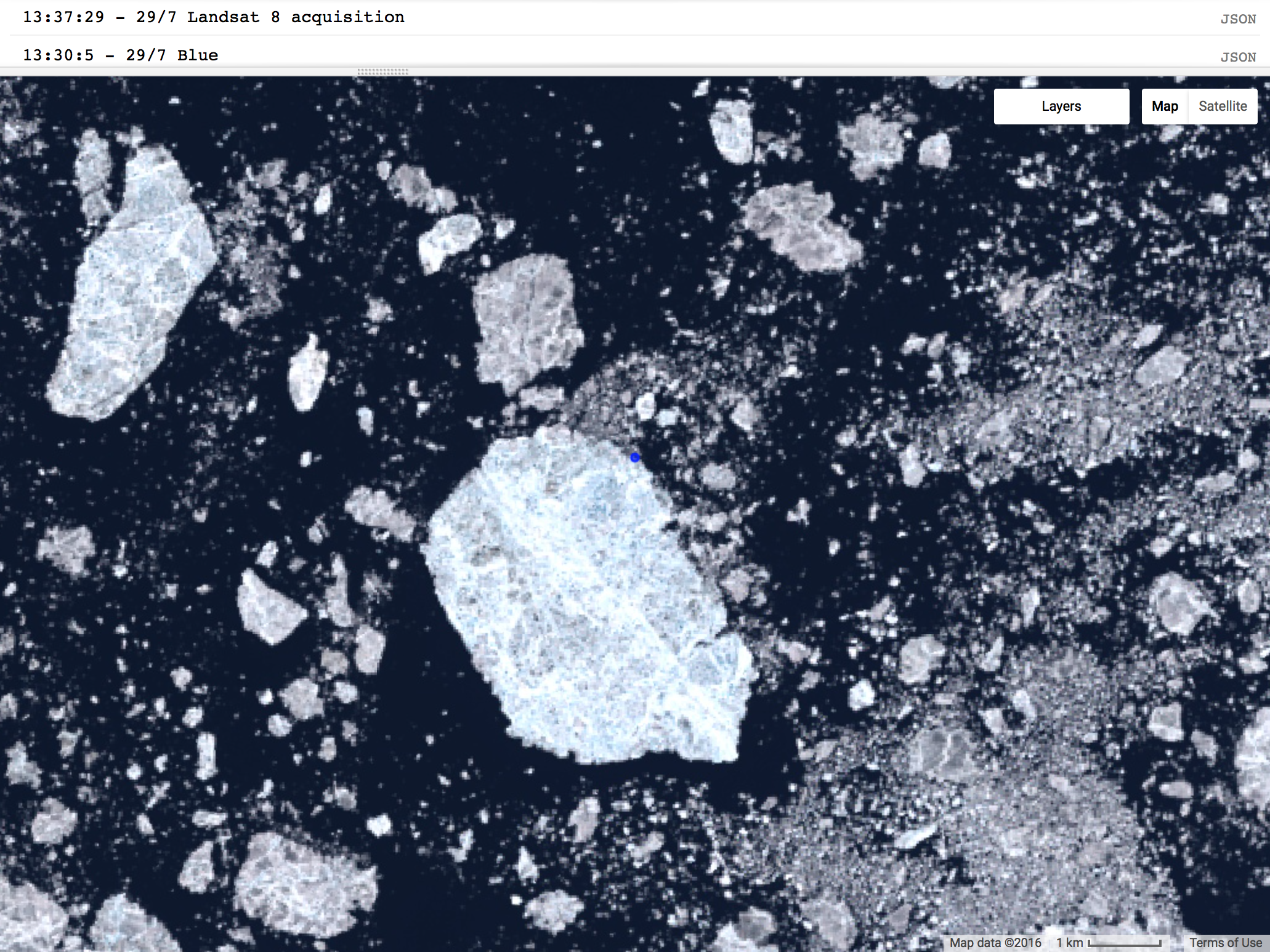

Supplement: Supplementary file 2 — Supplementary material [file mmc2.zip › GPS_tracker_data_python_plots_satellite/GPS_tracker_sat_data/landsat8/L8_20160729_b.png]

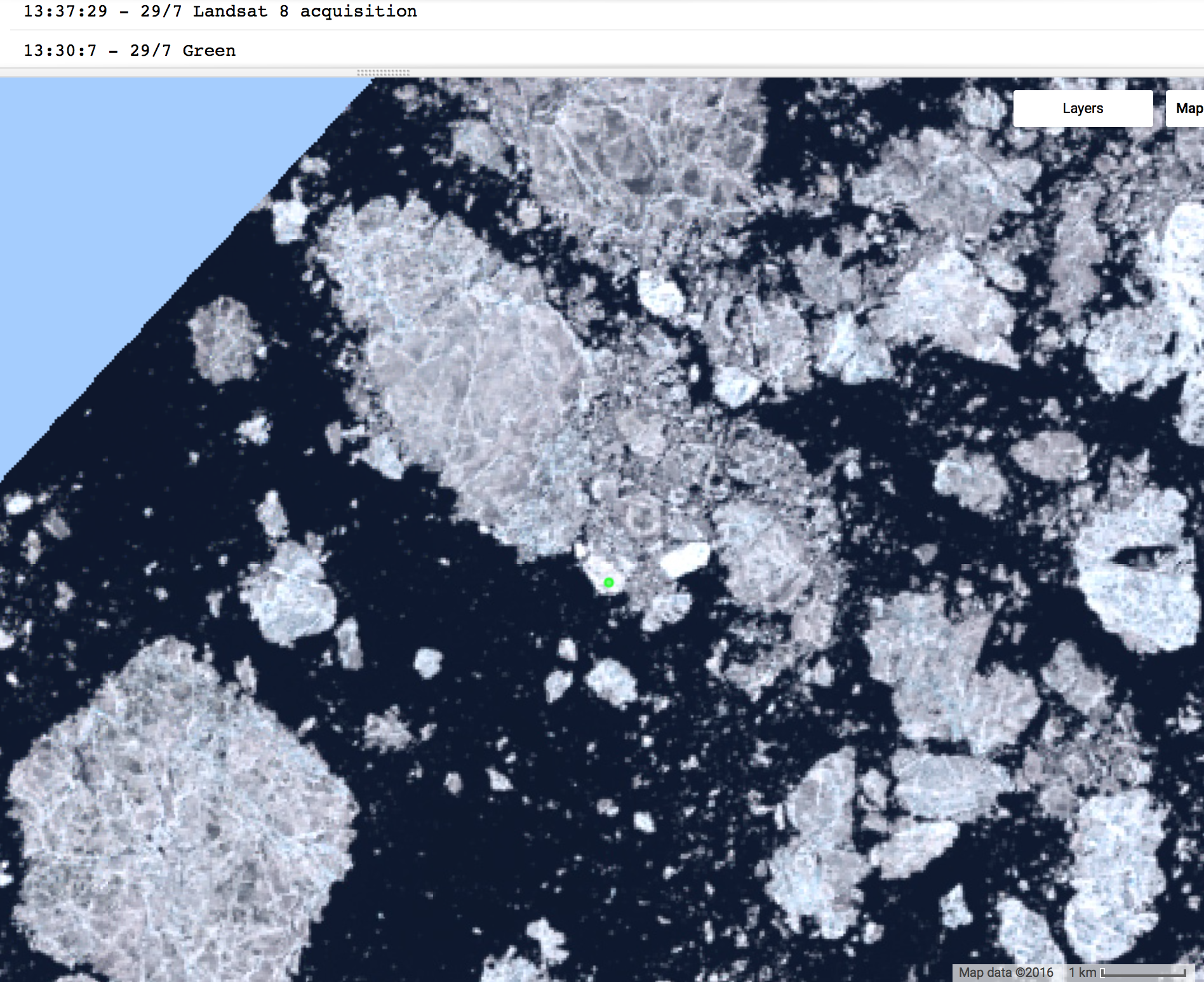

Supplement: Supplementary file 2 — Supplementary material [file mmc2.zip › GPS_tracker_data_python_plots_satellite/GPS_tracker_sat_data/landsat8/L8_20160729_g.png]

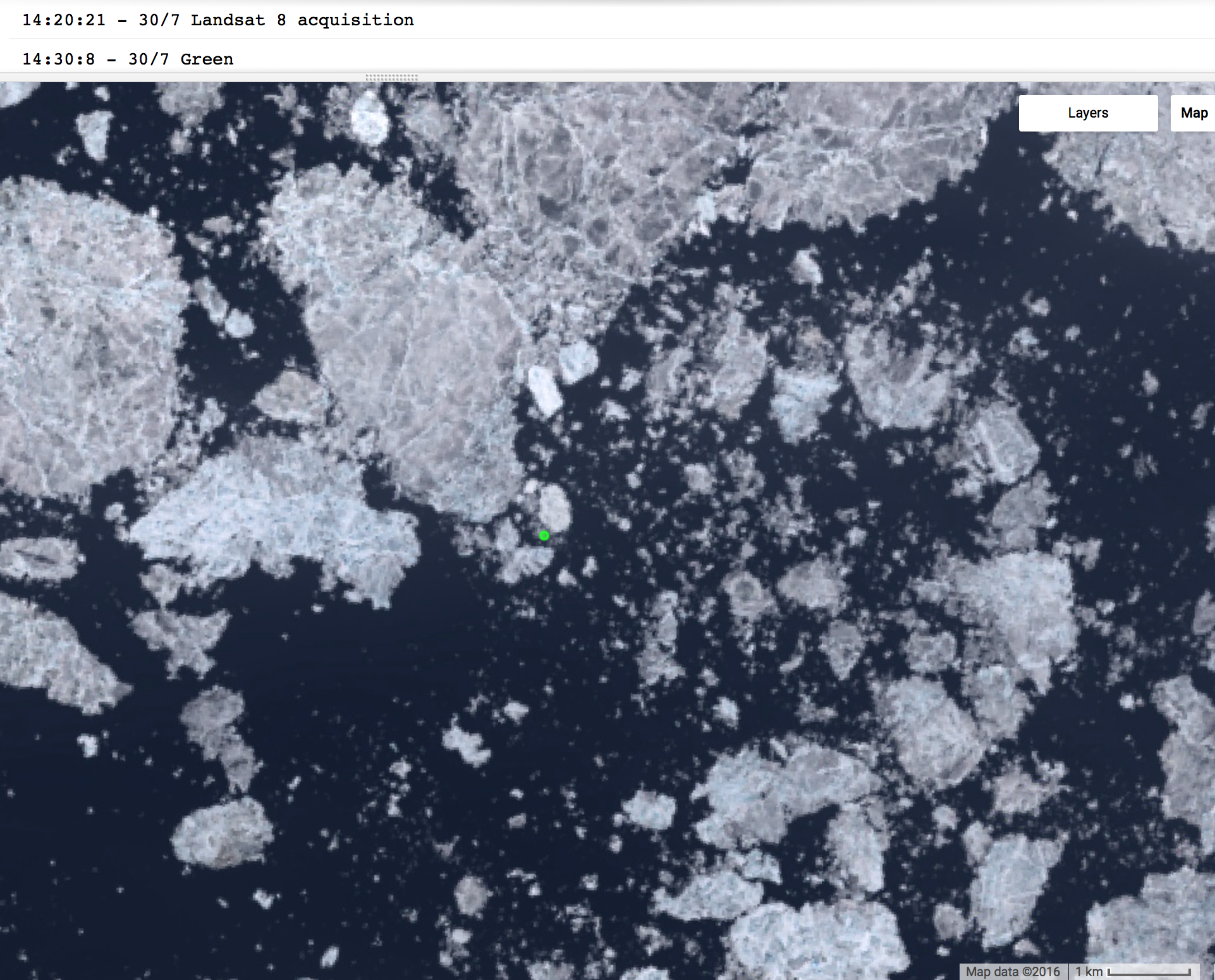

Supplement: Supplementary file 2 — Supplementary material [file mmc2.zip › GPS_tracker_data_python_plots_satellite/GPS_tracker_sat_data/landsat8/L8_20160730_g.png]

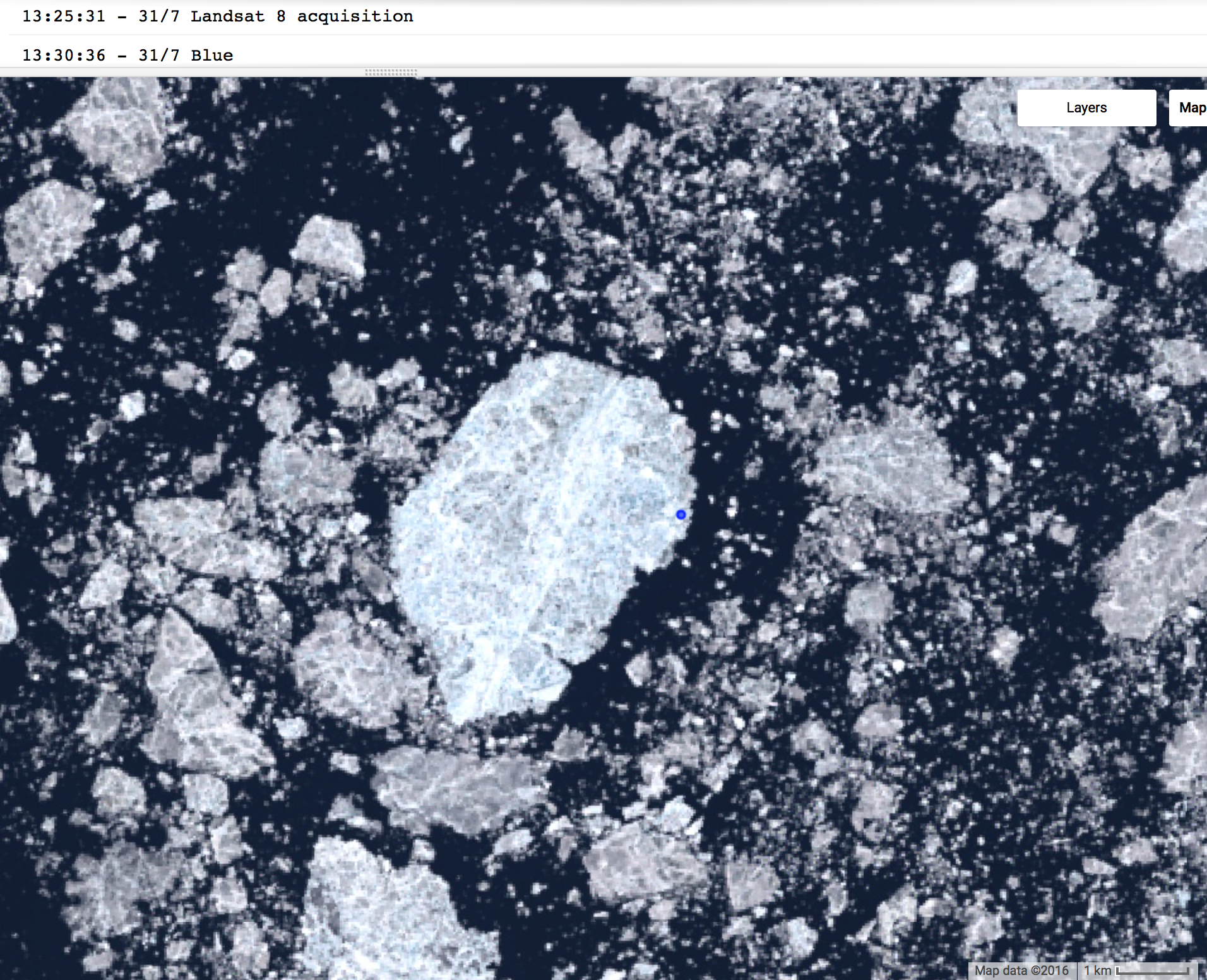

Supplement: Supplementary file 2 — Supplementary material [file mmc2.zip › GPS_tracker_data_python_plots_satellite/GPS_tracker_sat_data/landsat8/L8_20160731_b.png]

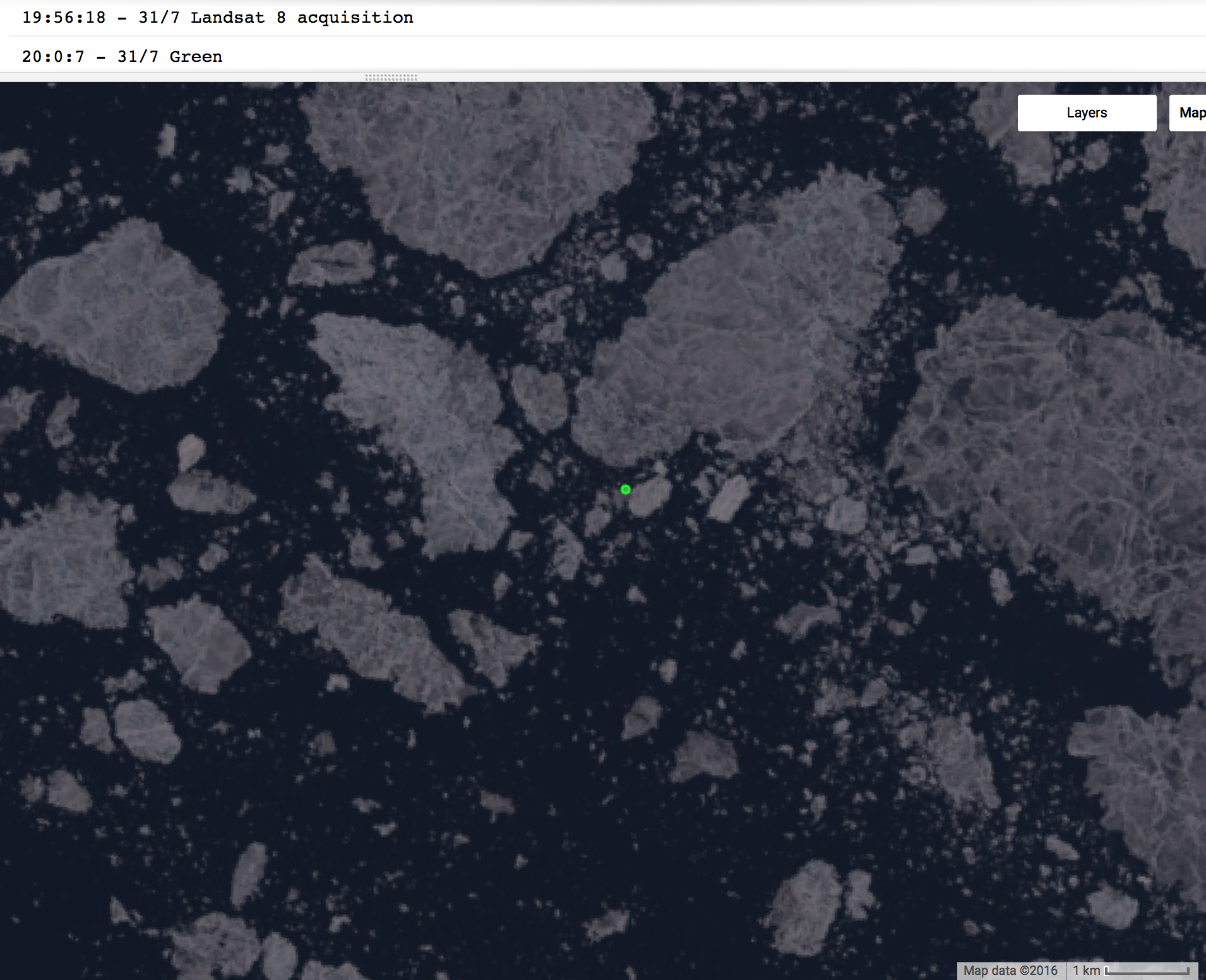

Supplement: Supplementary file 2 — Supplementary material [file mmc2.zip › GPS_tracker_data_python_plots_satellite/GPS_tracker_sat_data/landsat8/L8_20160731_g.png]

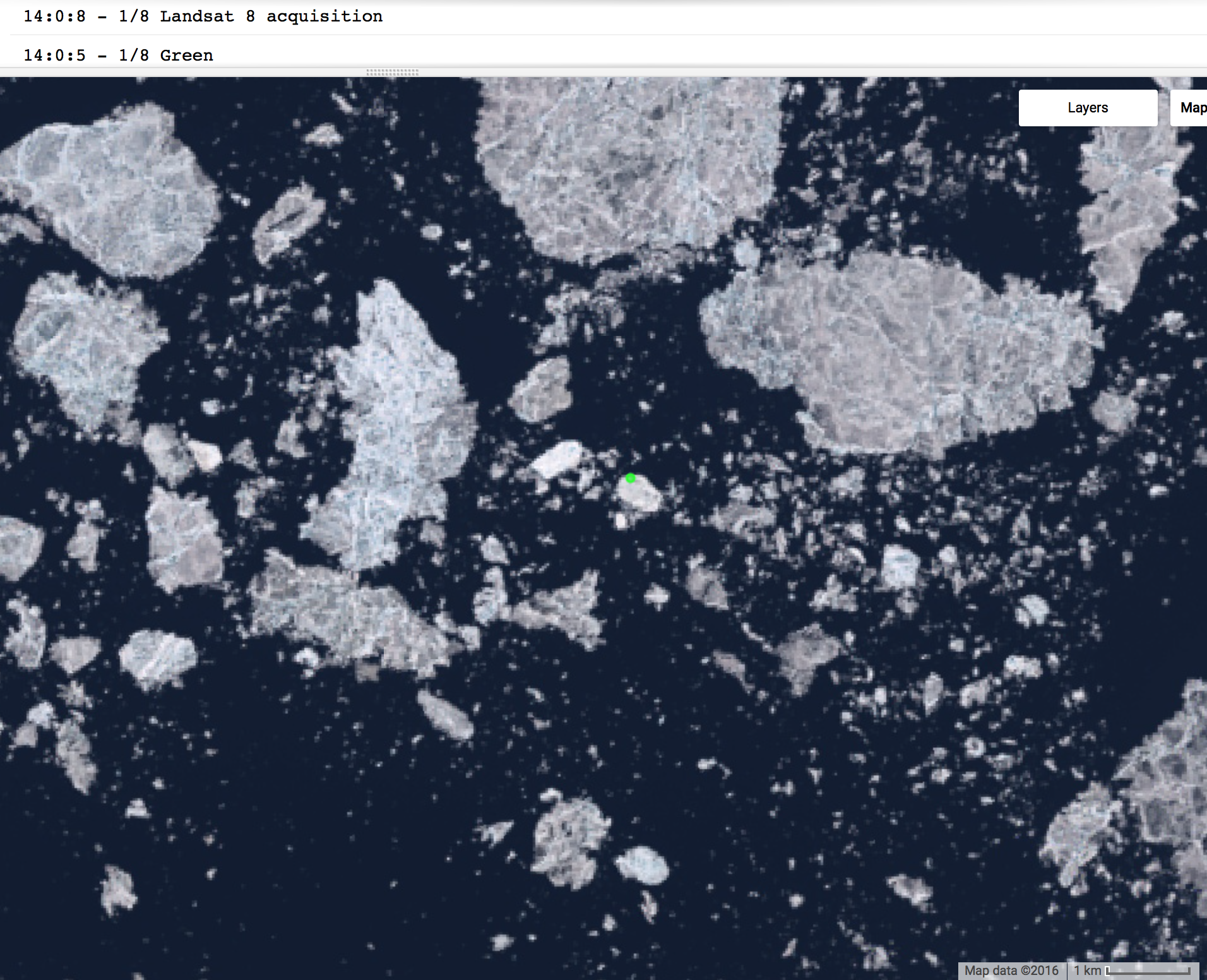

Supplement: Supplementary file 2 — Supplementary material [file mmc2.zip › GPS_tracker_data_python_plots_satellite/GPS_tracker_sat_data/landsat8/L8_20160801_g.png]

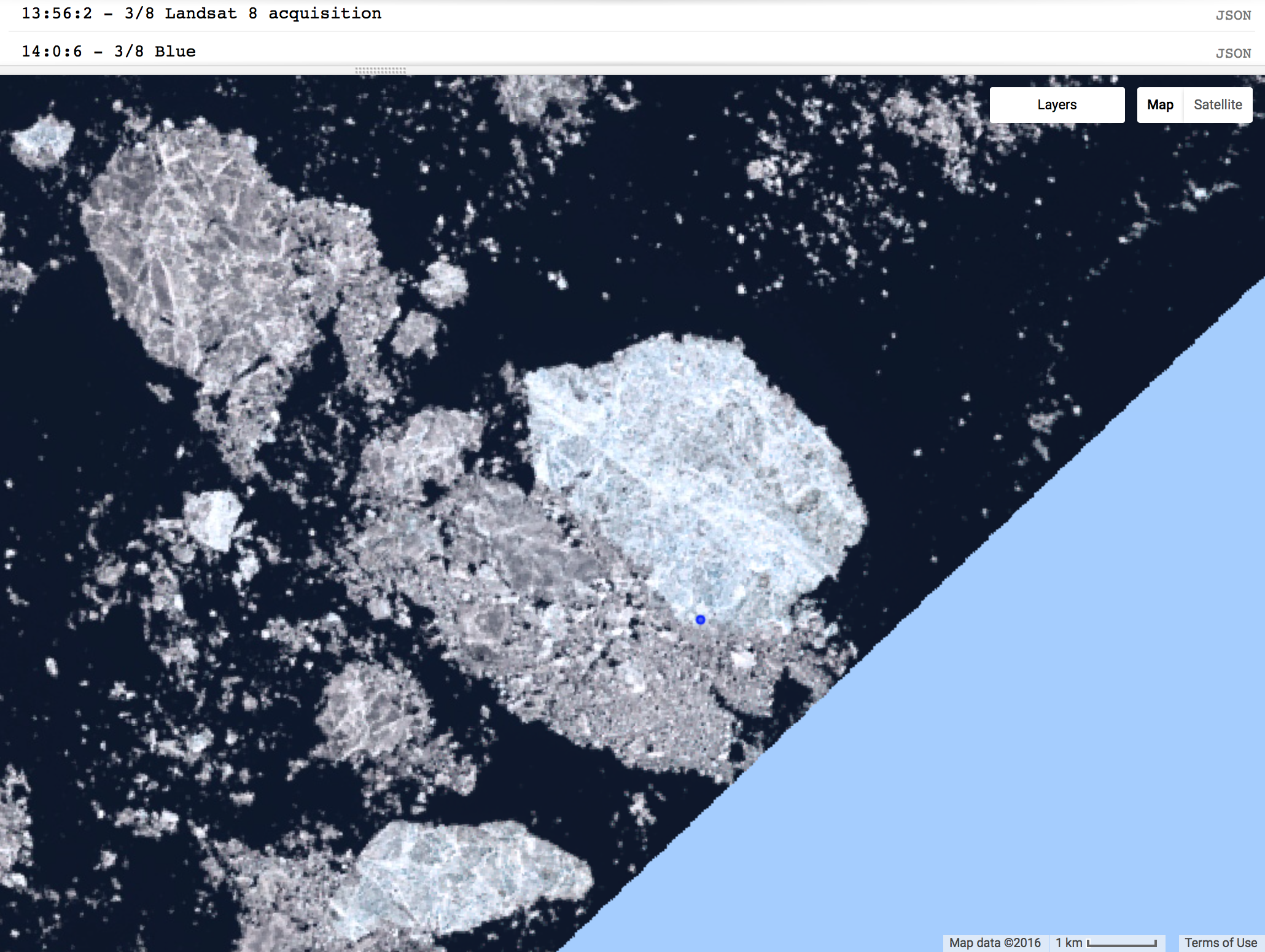

Supplement: Supplementary file 2 — Supplementary material [file mmc2.zip › GPS_tracker_data_python_plots_satellite/GPS_tracker_sat_data/landsat8/L8_20160803_b.png]

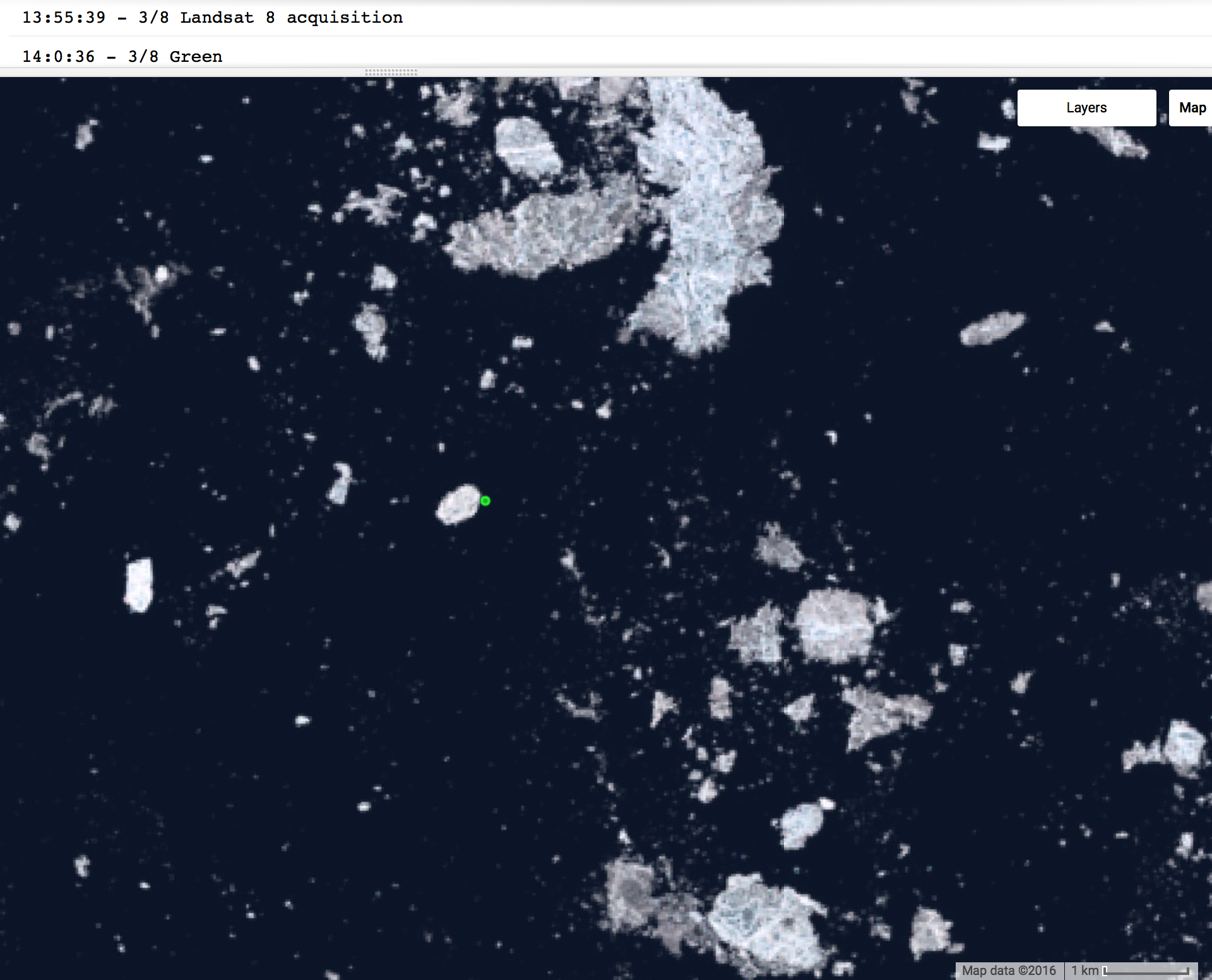

Supplement: Supplementary file 2 — Supplementary material [file mmc2.zip › GPS_tracker_data_python_plots_satellite/GPS_tracker_sat_data/landsat8/L8_20160803_g.png]

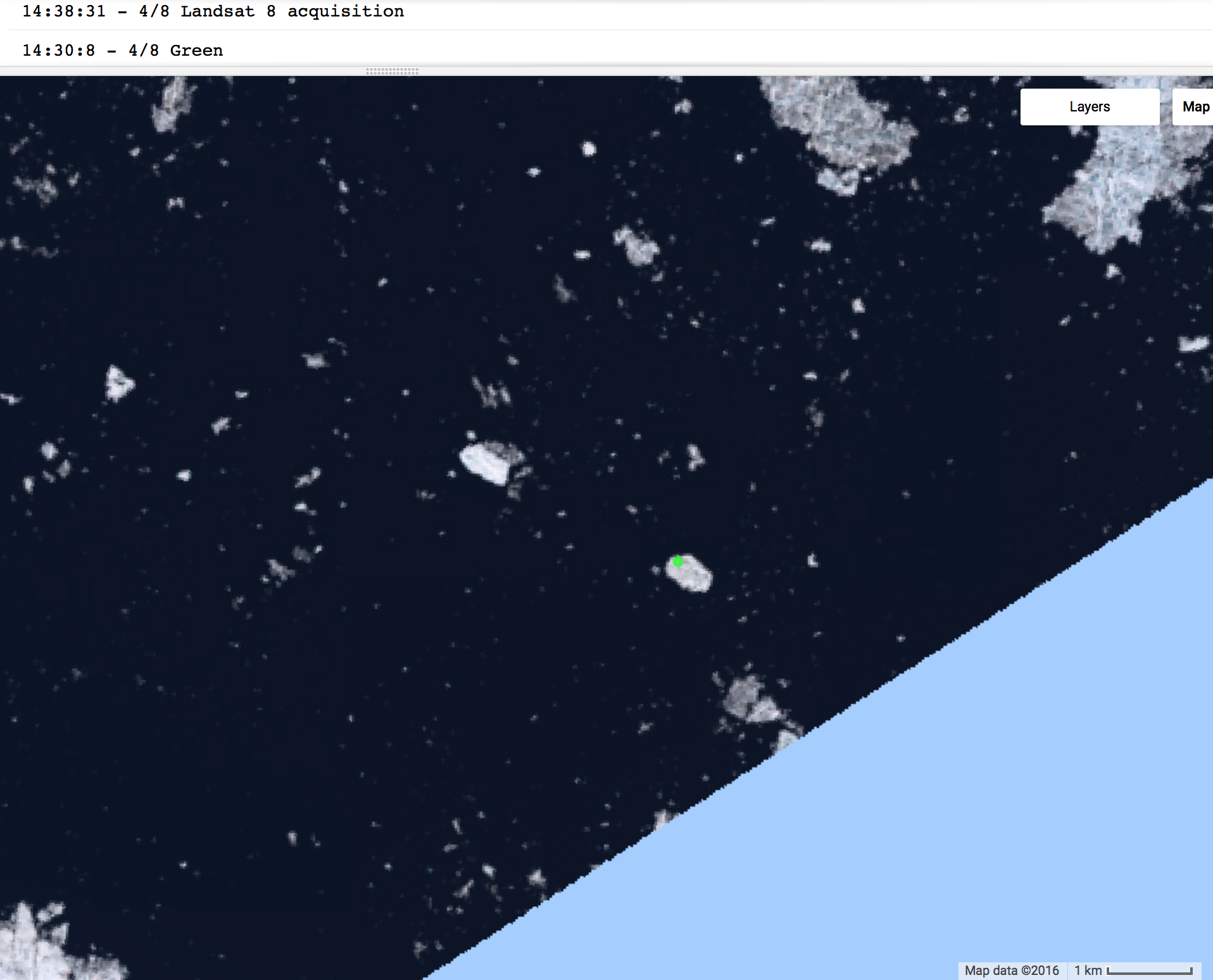

Supplement: Supplementary file 2 — Supplementary material [file mmc2.zip › GPS_tracker_data_python_plots_satellite/GPS_tracker_sat_data/landsat8/L8_20160804_g.png]

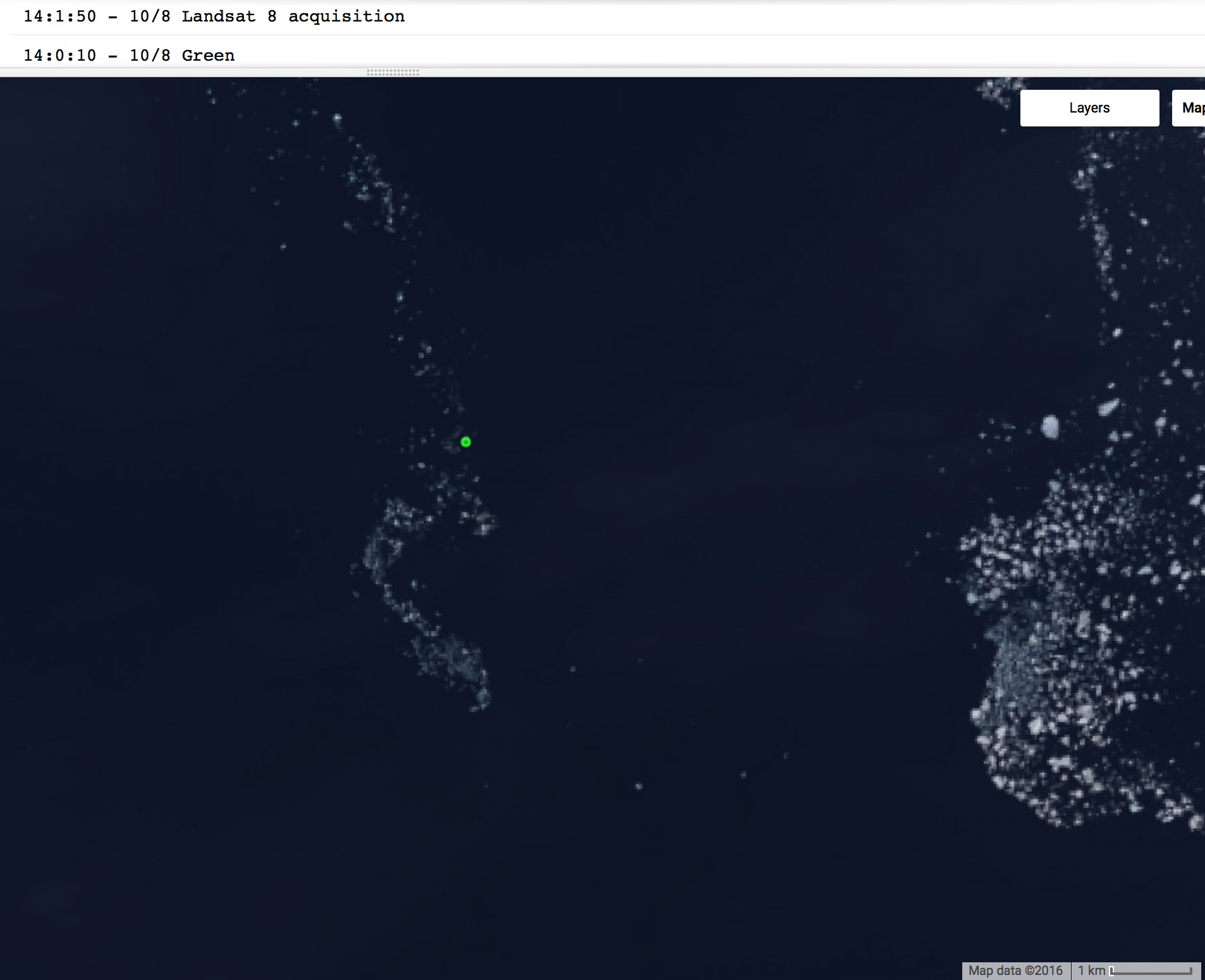

Supplement: Supplementary file 2 — Supplementary material [file mmc2.zip › GPS_tracker_data_python_plots_satellite/GPS_tracker_sat_data/landsat8/L8_20160810_g.png]

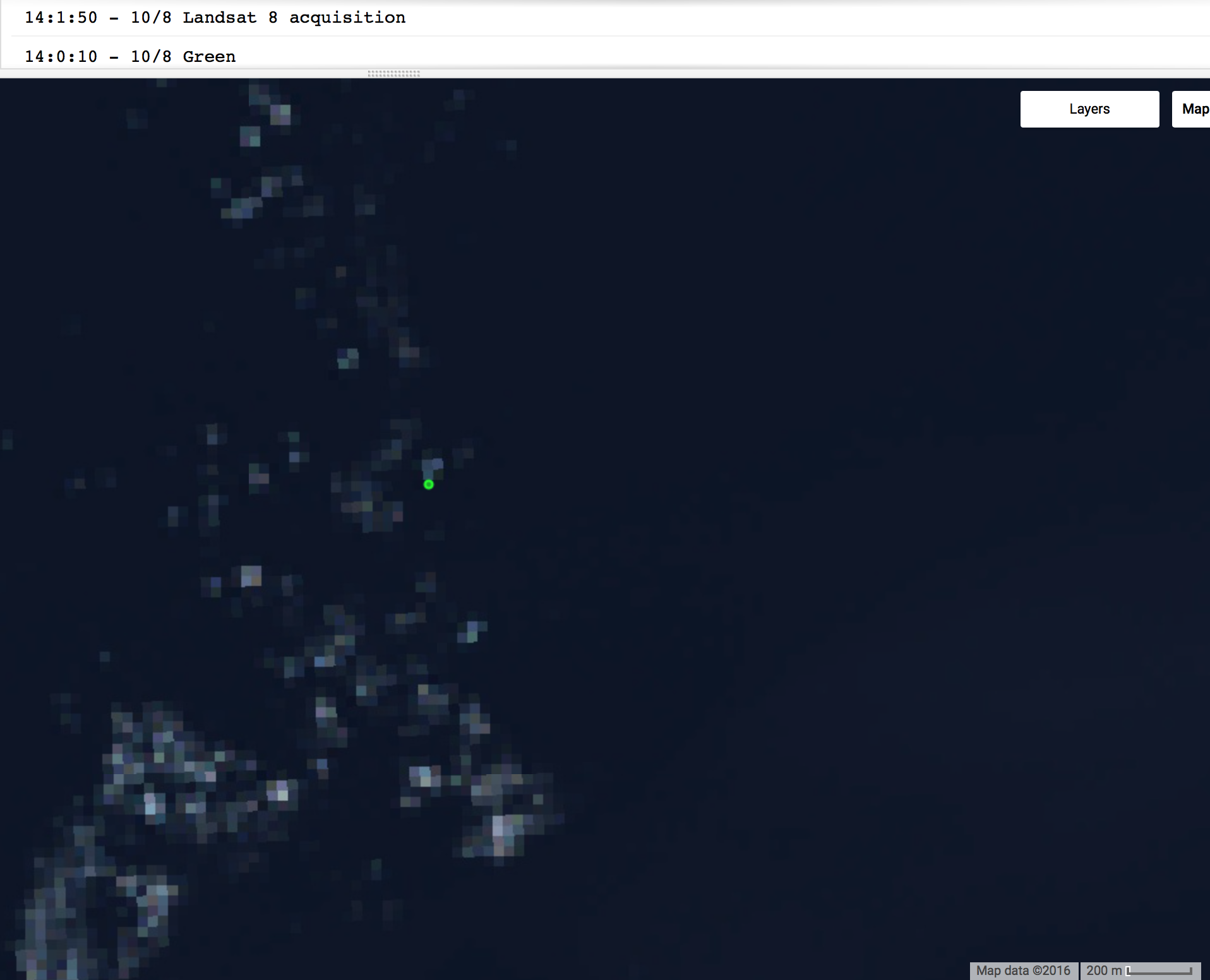

Supplement: Supplementary file 2 — Supplementary material [file mmc2.zip › GPS_tracker_data_python_plots_satellite/GPS_tracker_sat_data/landsat8/L8_20160810_g_200m.png]

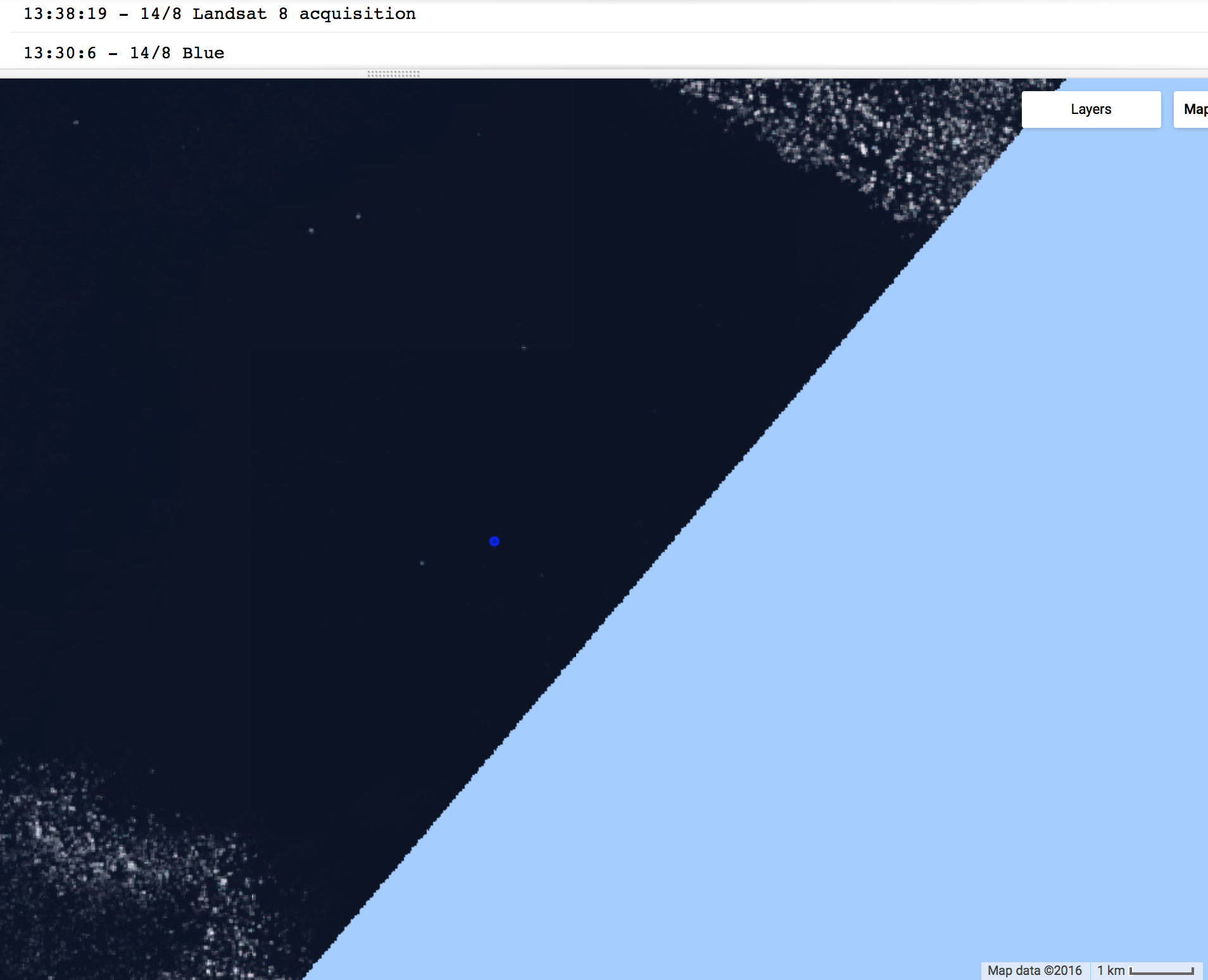

Supplement: Supplementary file 2 — Supplementary material [file mmc2.zip › GPS_tracker_data_python_plots_satellite/GPS_tracker_sat_data/landsat8/L8_20160814_b.png]

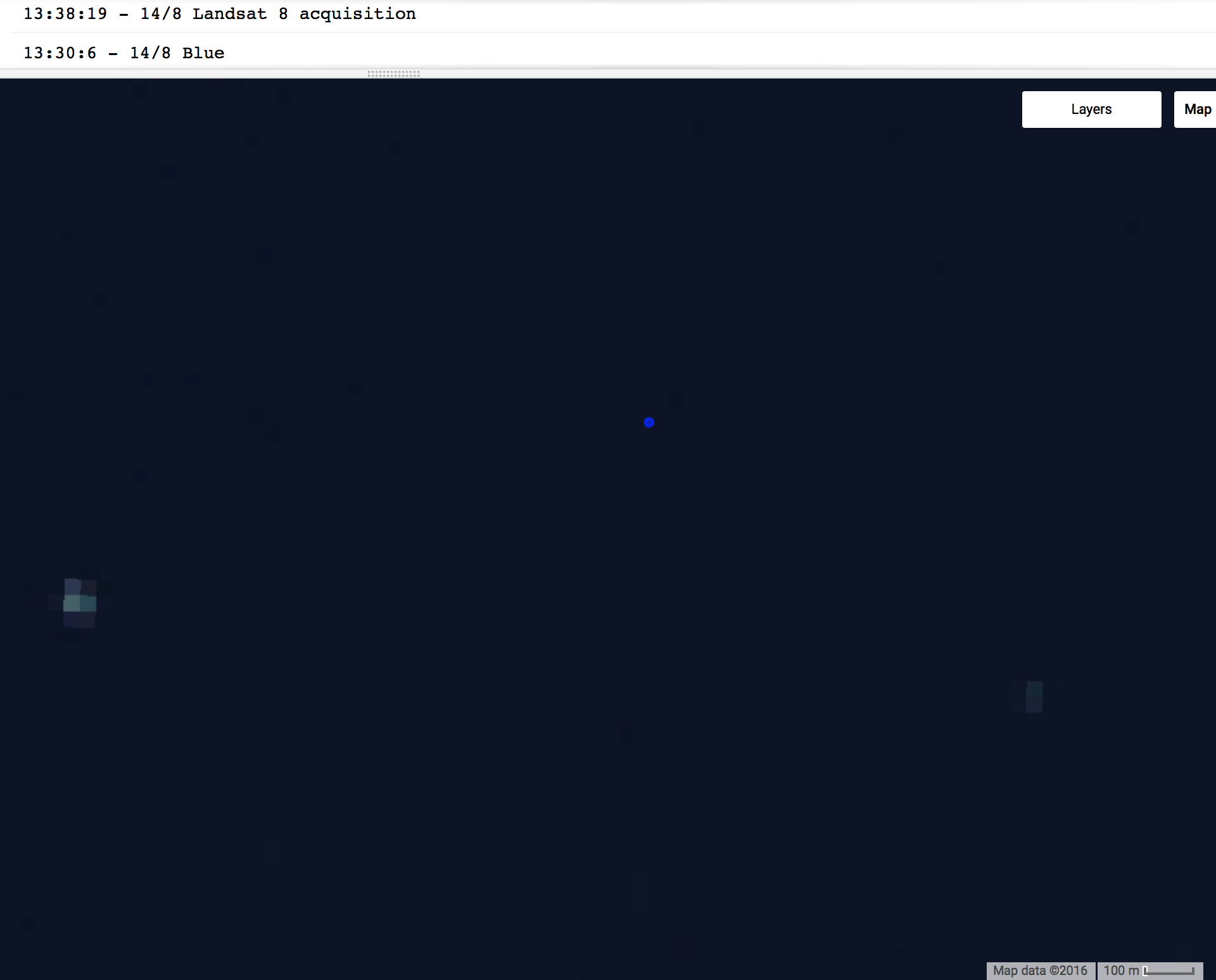

Supplement: Supplementary file 2 — Supplementary material [file mmc2.zip › GPS_tracker_data_python_plots_satellite/GPS_tracker_sat_data/landsat8/L8_20160814_b_100m.png]

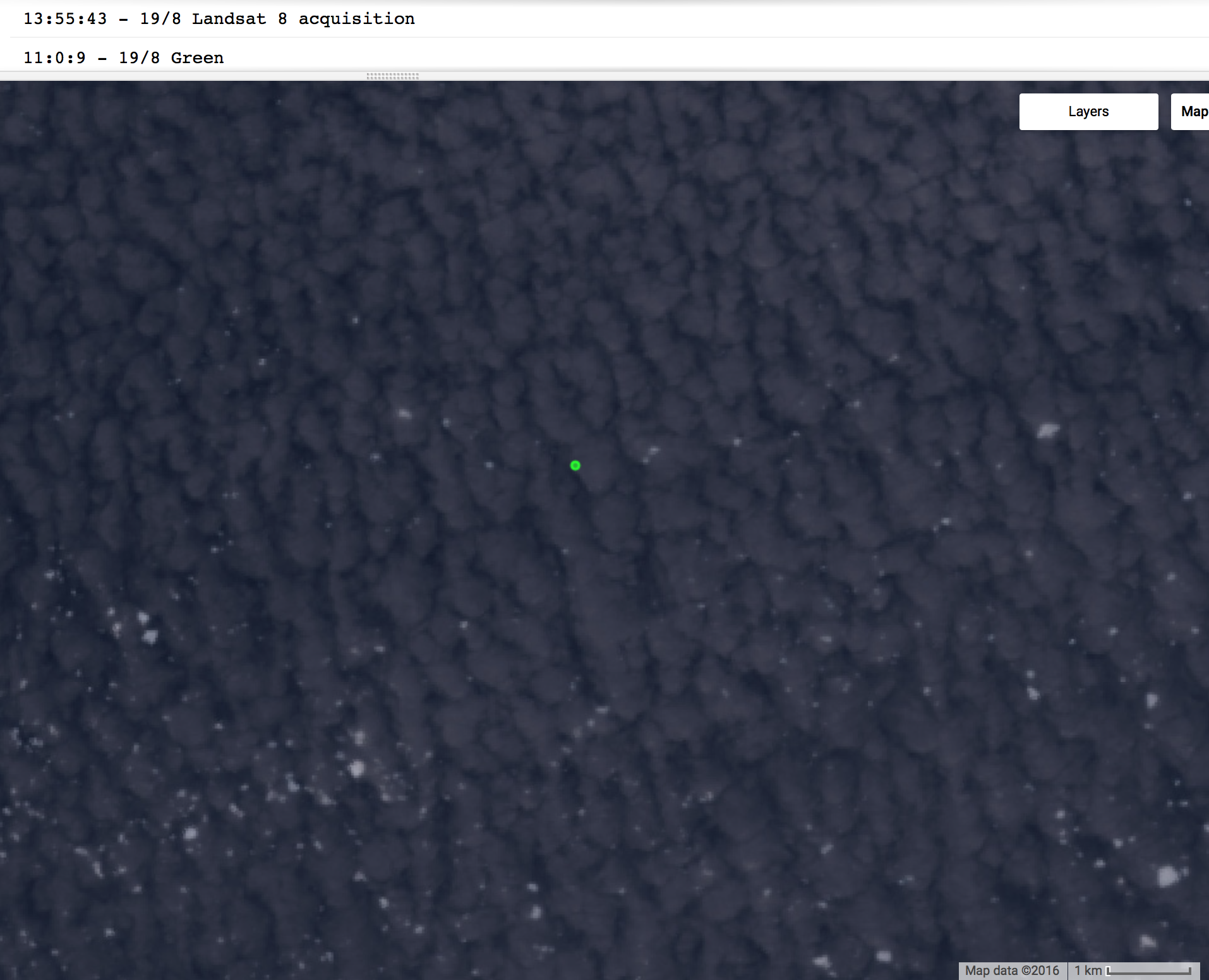

Supplement: Supplementary file 2 — Supplementary material [file mmc2.zip › GPS_tracker_data_python_plots_satellite/GPS_tracker_sat_data/landsat8/L8_20160819_g.png]

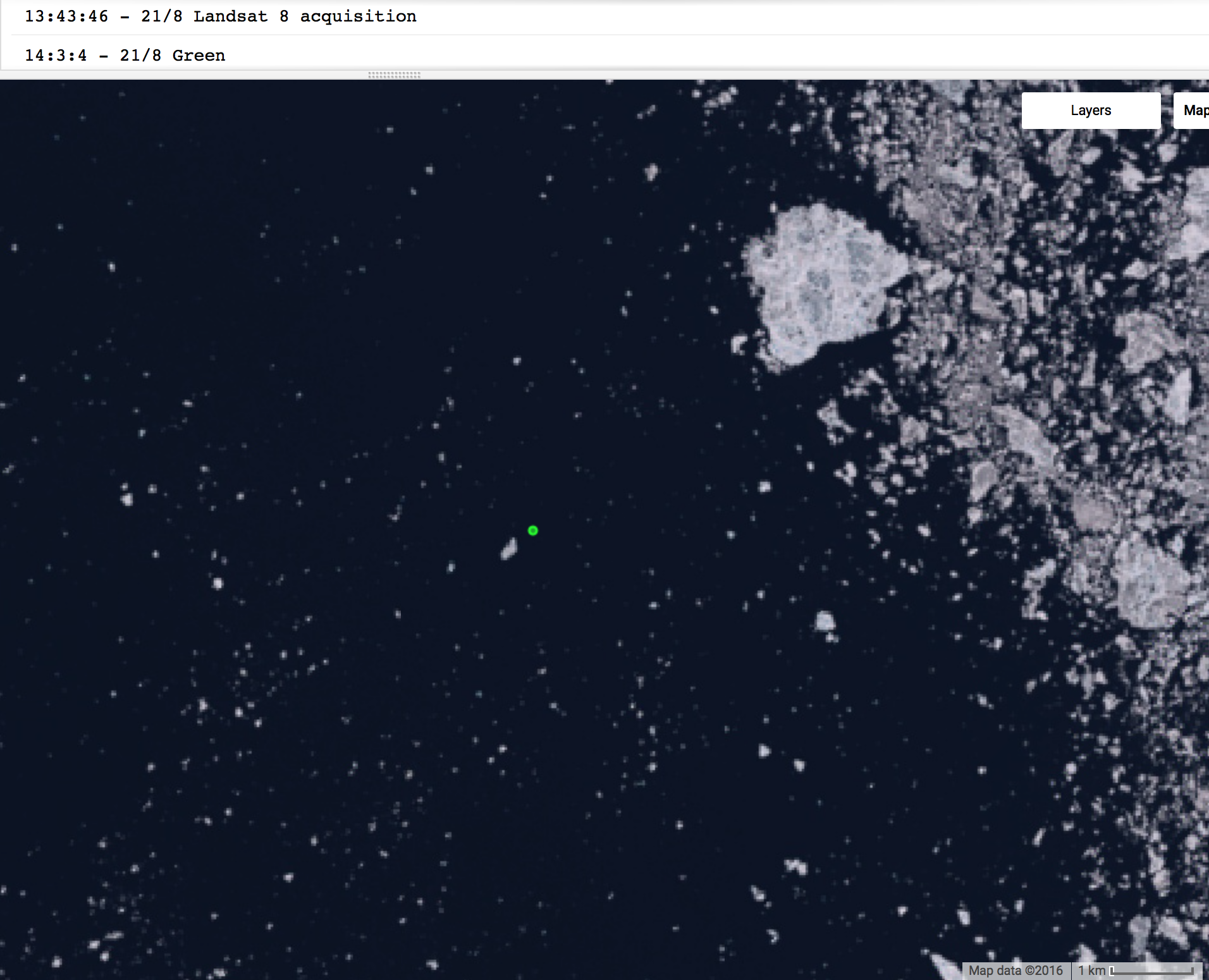

Supplement: Supplementary file 2 — Supplementary material [file mmc2.zip › GPS_tracker_data_python_plots_satellite/GPS_tracker_sat_data/landsat8/L8_20160821_g.png]

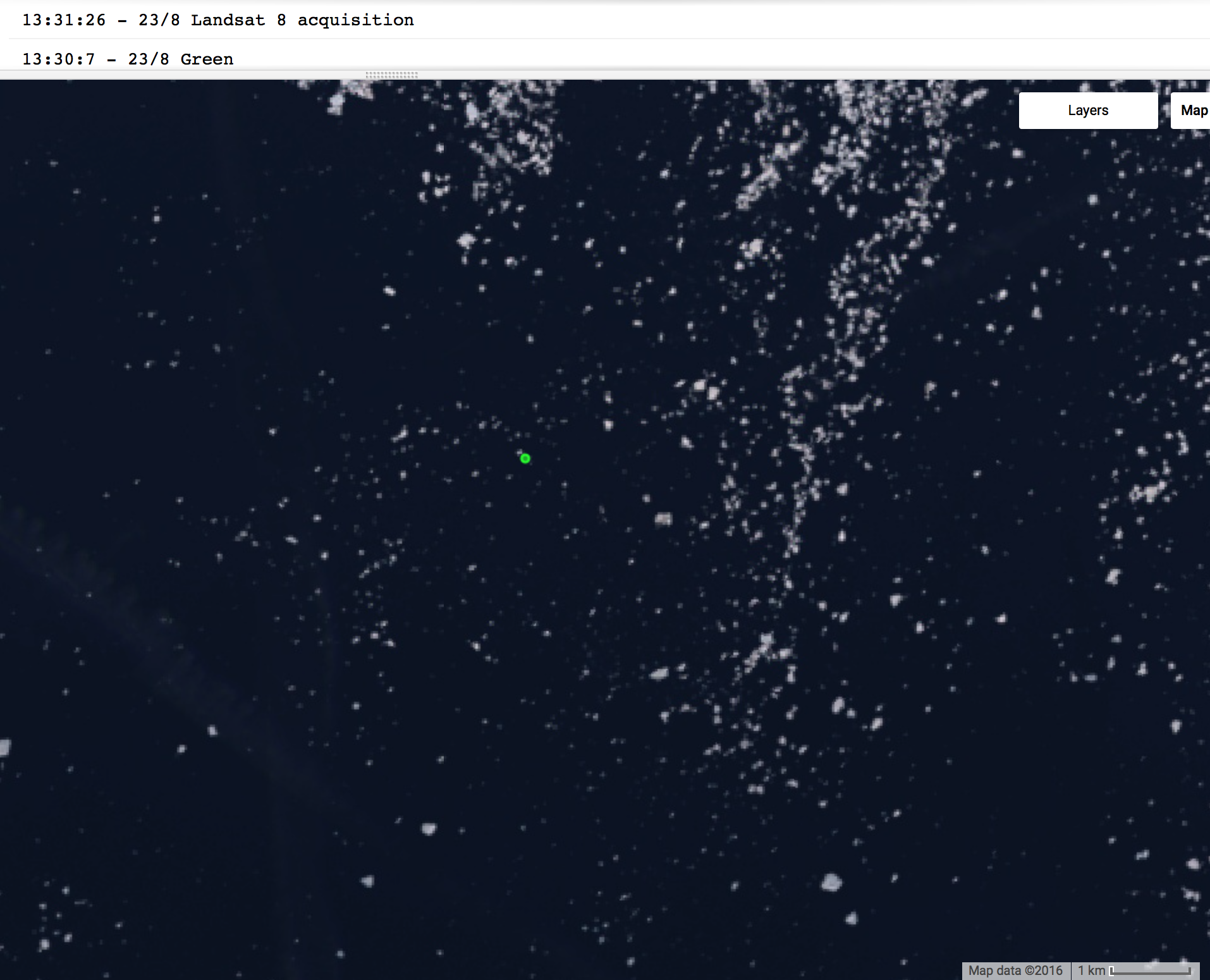

Supplement: Supplementary file 2 — Supplementary material [file mmc2.zip › GPS_tracker_data_python_plots_satellite/GPS_tracker_sat_data/landsat8/L8_20160823_g.png]

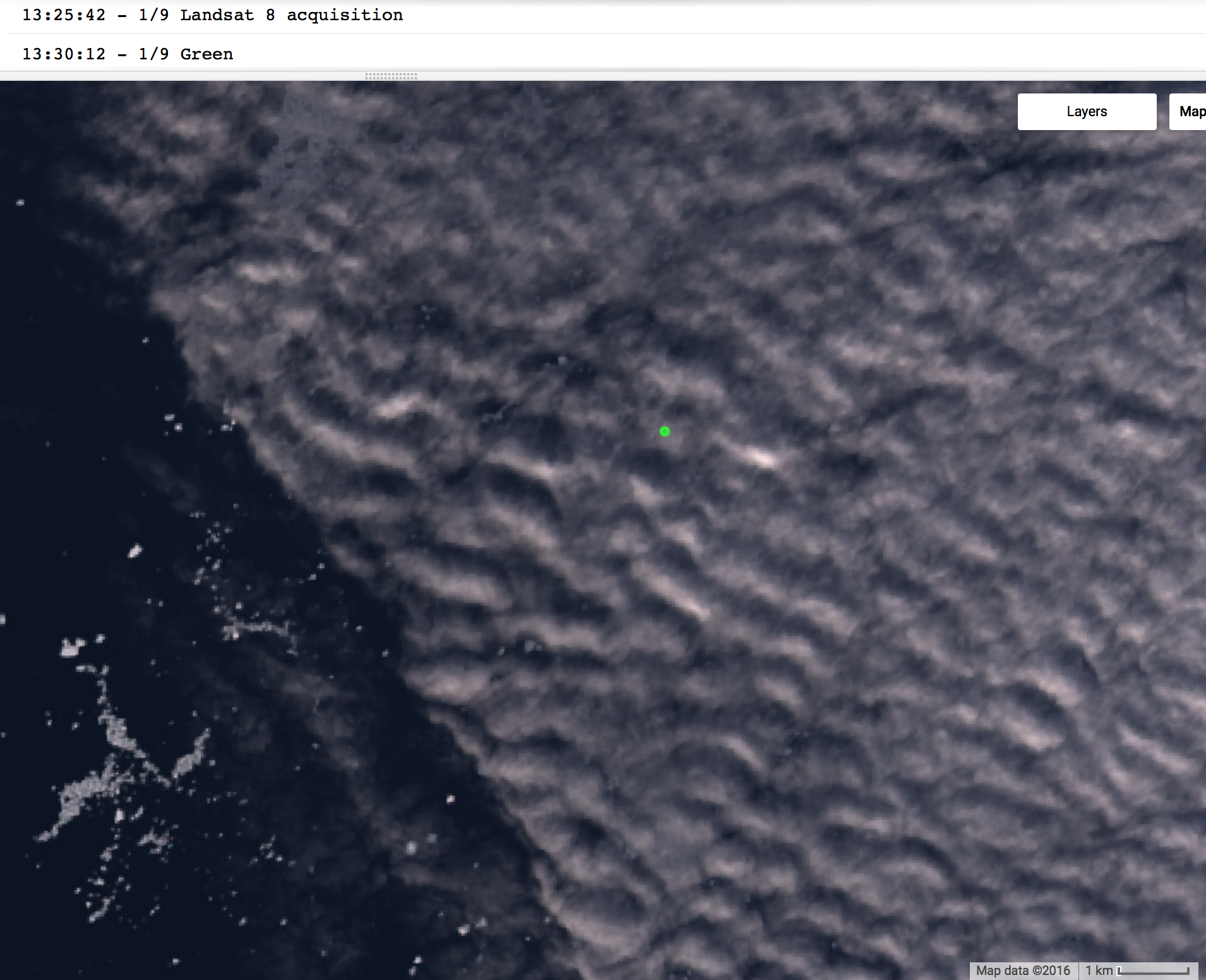

Supplement: Supplementary file 2 — Supplementary material [file mmc2.zip › GPS_tracker_data_python_plots_satellite/GPS_tracker_sat_data/landsat8/L8_20160901_g.png]

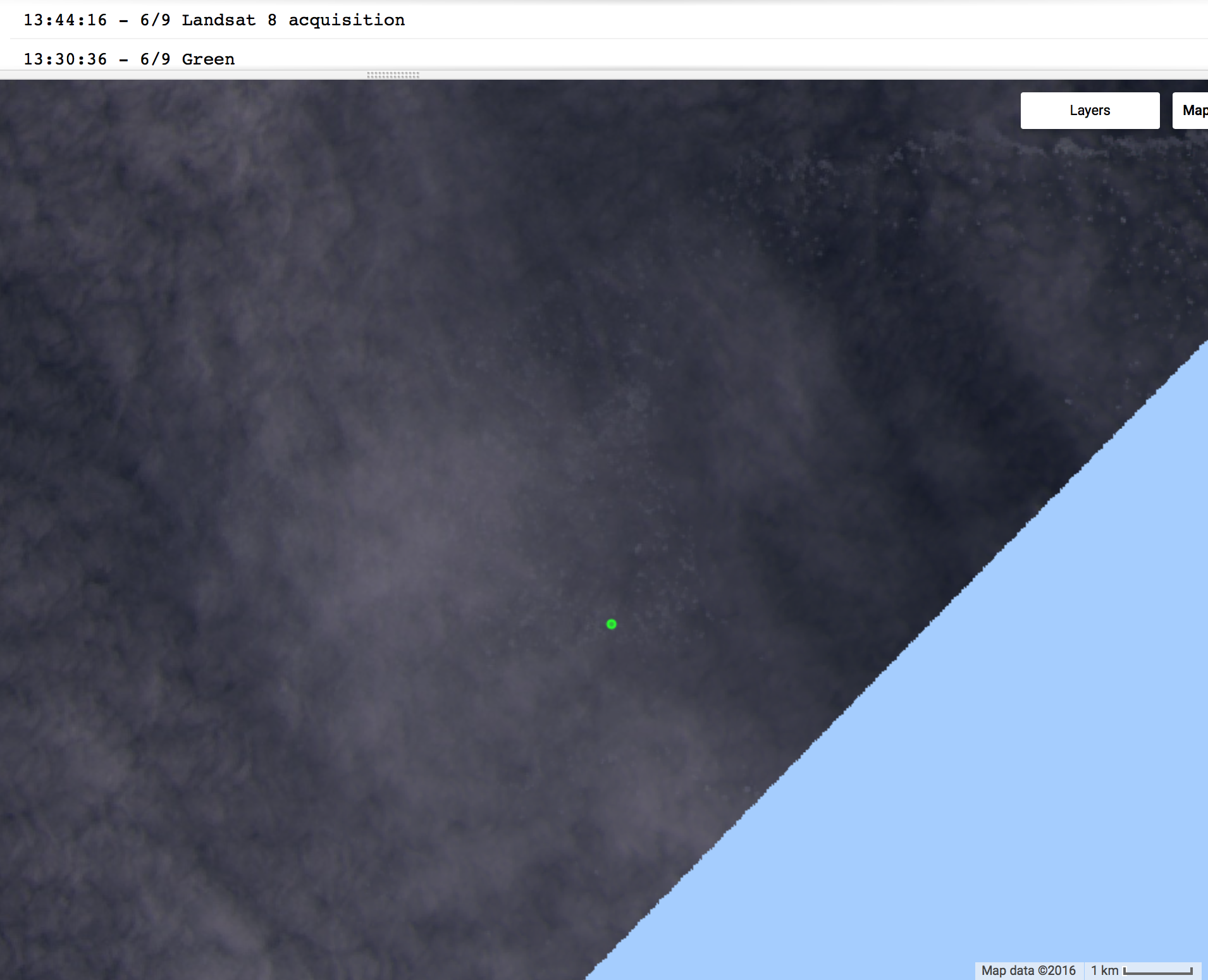

Supplement: Supplementary file 2 — Supplementary material [file mmc2.zip › GPS_tracker_data_python_plots_satellite/GPS_tracker_sat_data/landsat8/L8_20160906_g.png]

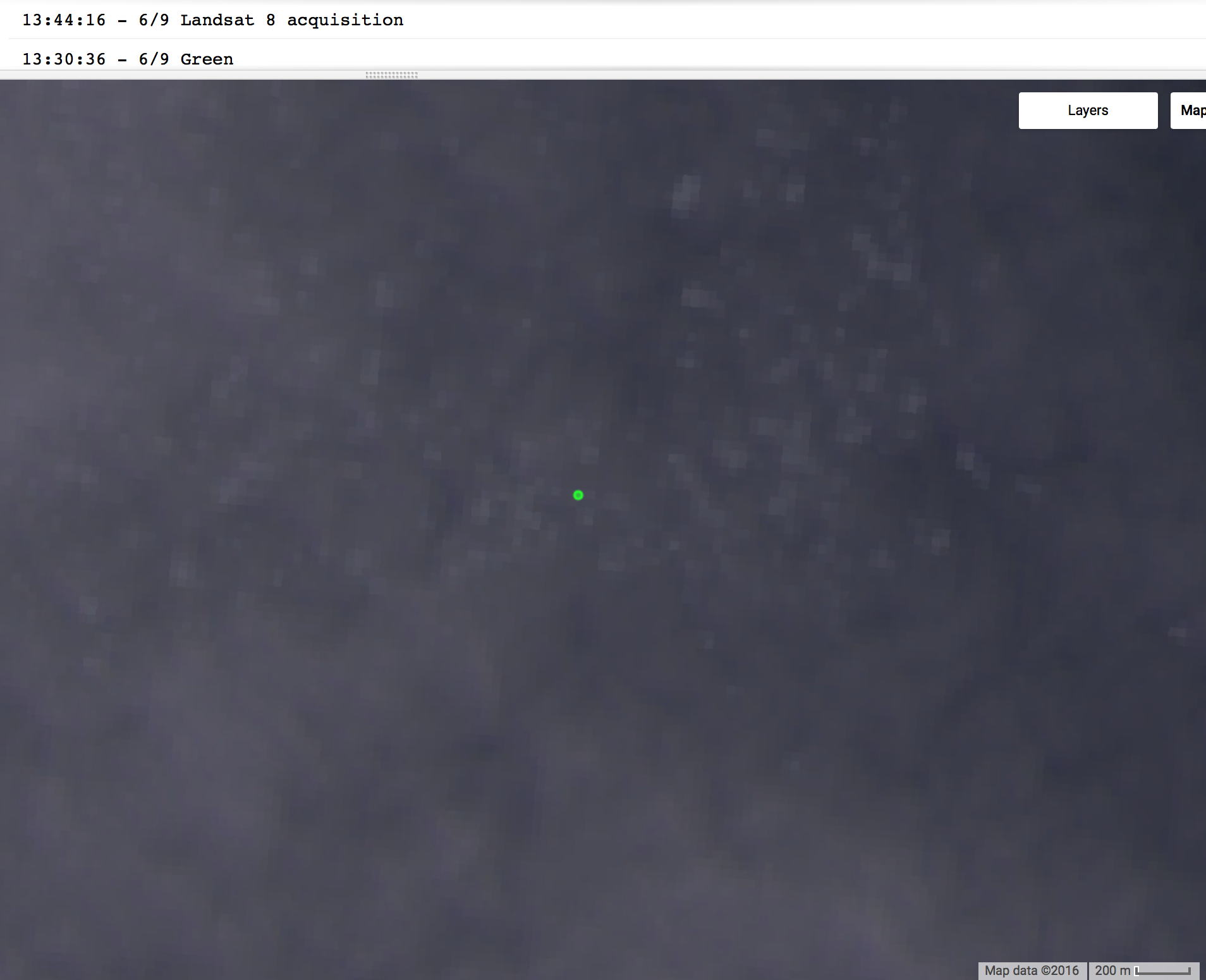

Supplement: Supplementary file 2 — Supplementary material [file mmc2.zip › GPS_tracker_data_python_plots_satellite/GPS_tracker_sat_data/landsat8/L8_20160906_g_200m.png]

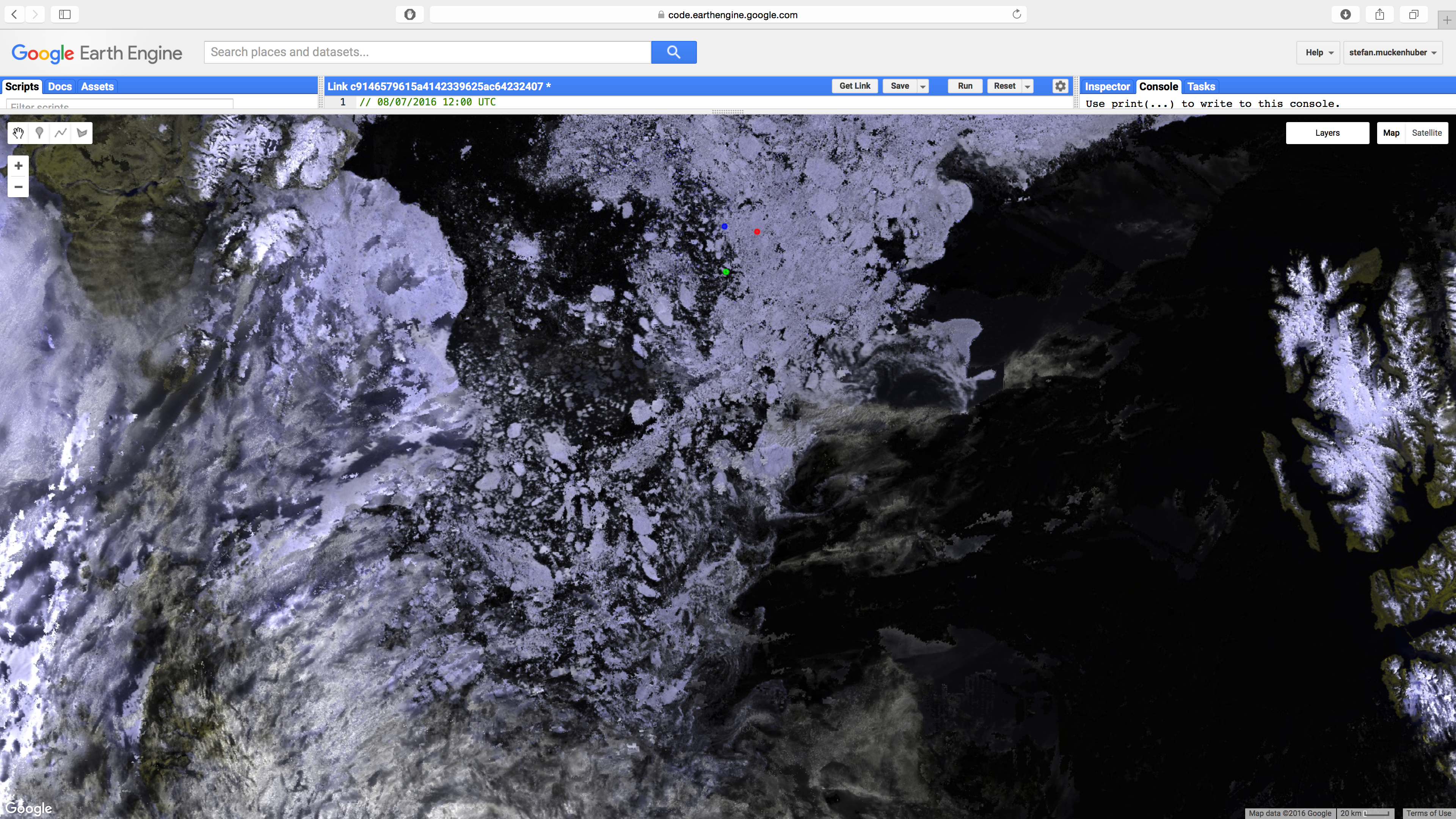

Supplement: Supplementary file 2 — Supplementary material [file mmc2.zip › GPS_tracker_data_python_plots_satellite/GPS_tracker_sat_data/MODIS_EE/MODIS_20160708.png]

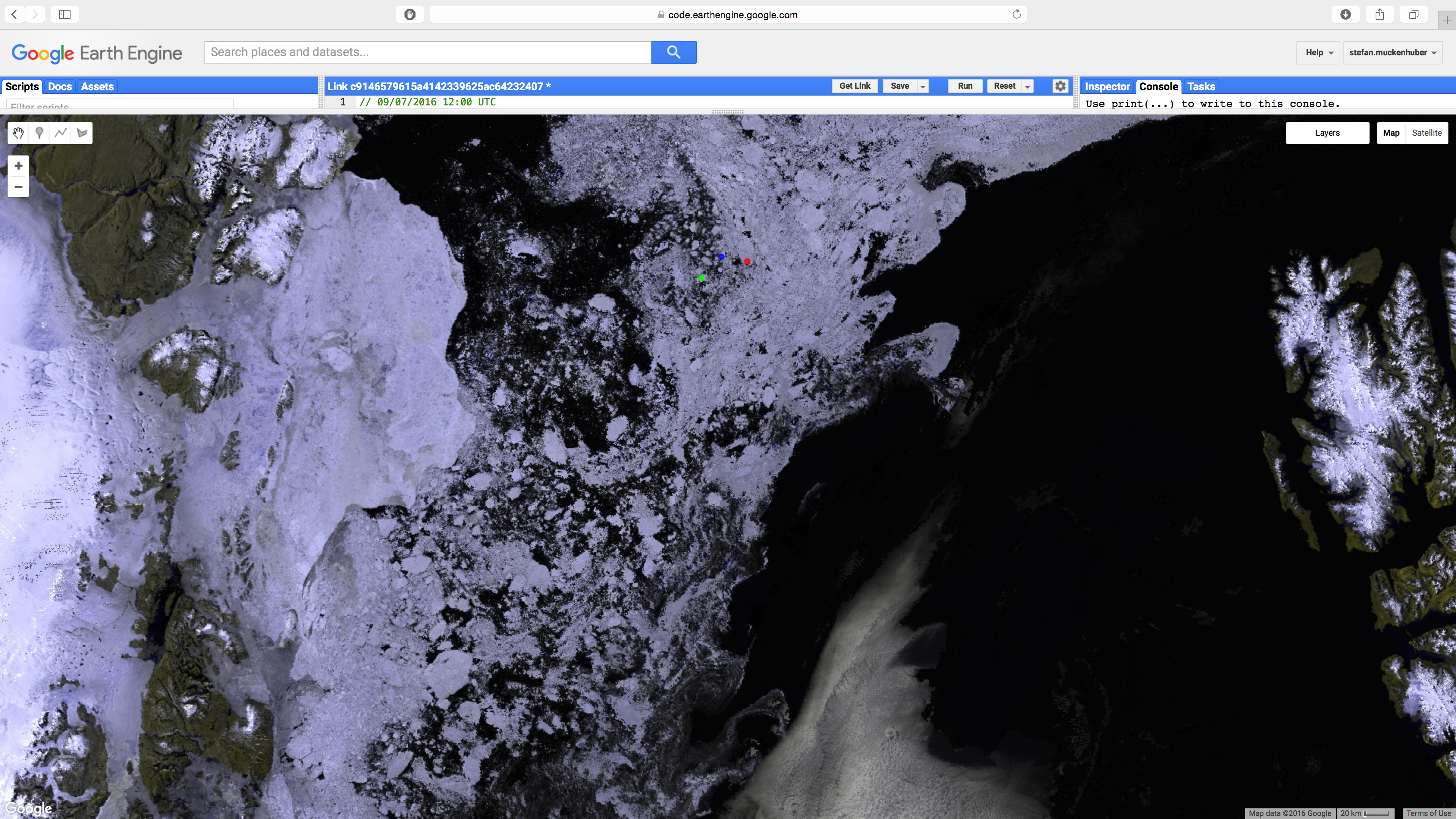

Supplement: Supplementary file 2 — Supplementary material [file mmc2.zip › GPS_tracker_data_python_plots_satellite/GPS_tracker_sat_data/MODIS_EE/MODIS_20160709.png]

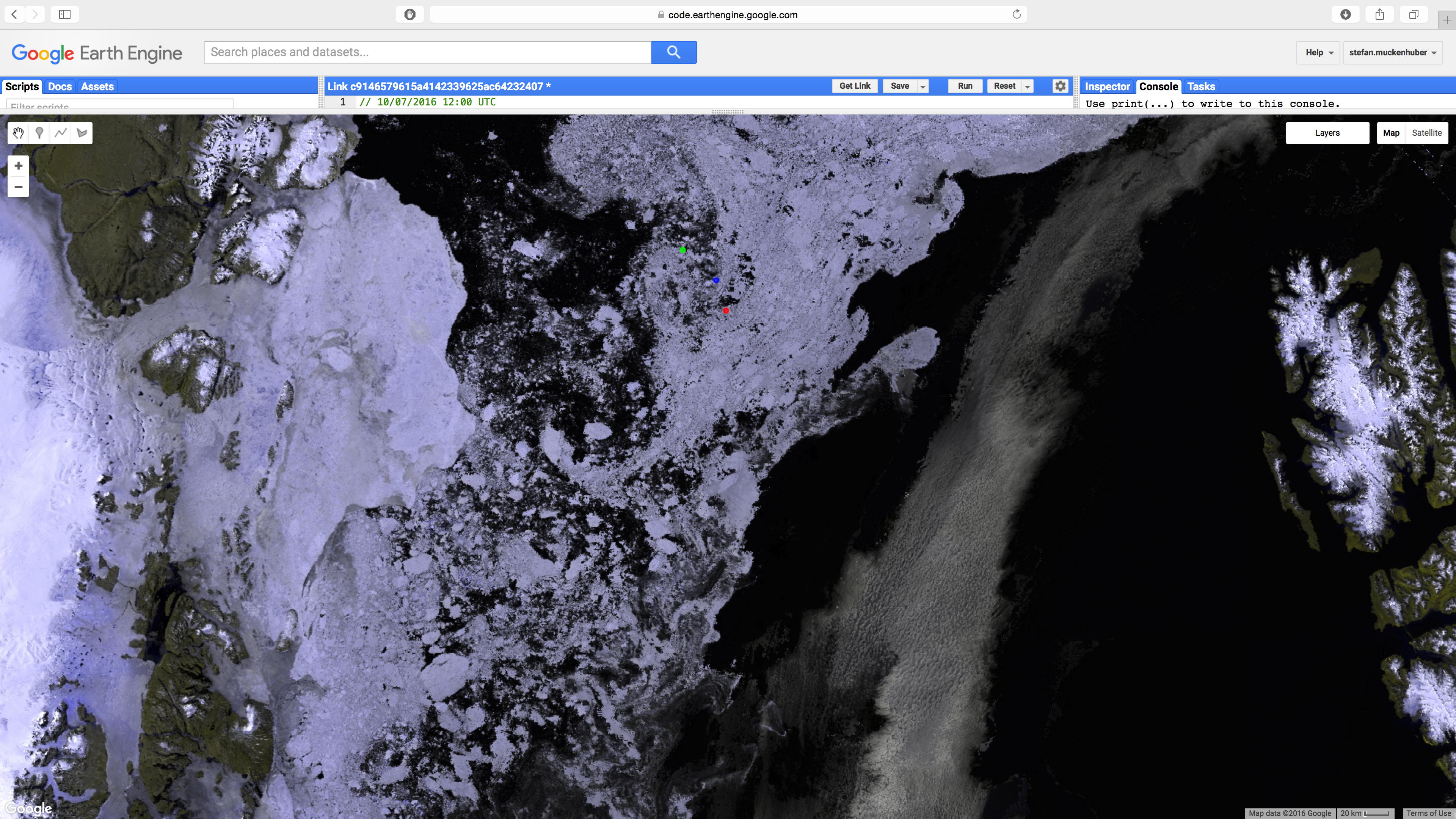

Supplement: Supplementary file 2 — Supplementary material [file mmc2.zip › GPS_tracker_data_python_plots_satellite/GPS_tracker_sat_data/MODIS_EE/MODIS_20160710.png]

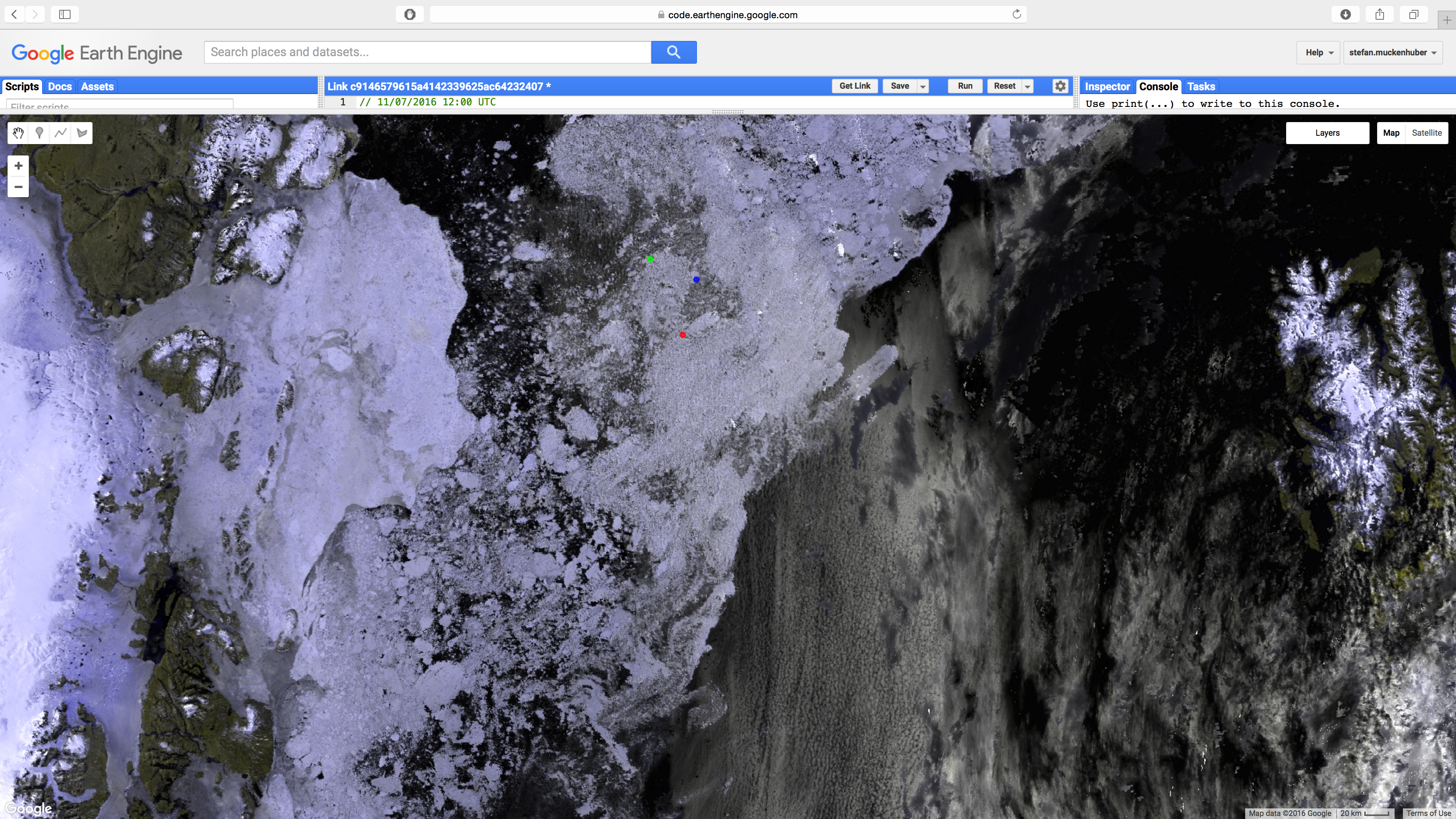

Supplement: Supplementary file 2 — Supplementary material [file mmc2.zip › GPS_tracker_data_python_plots_satellite/GPS_tracker_sat_data/MODIS_EE/MODIS_20160711.png]

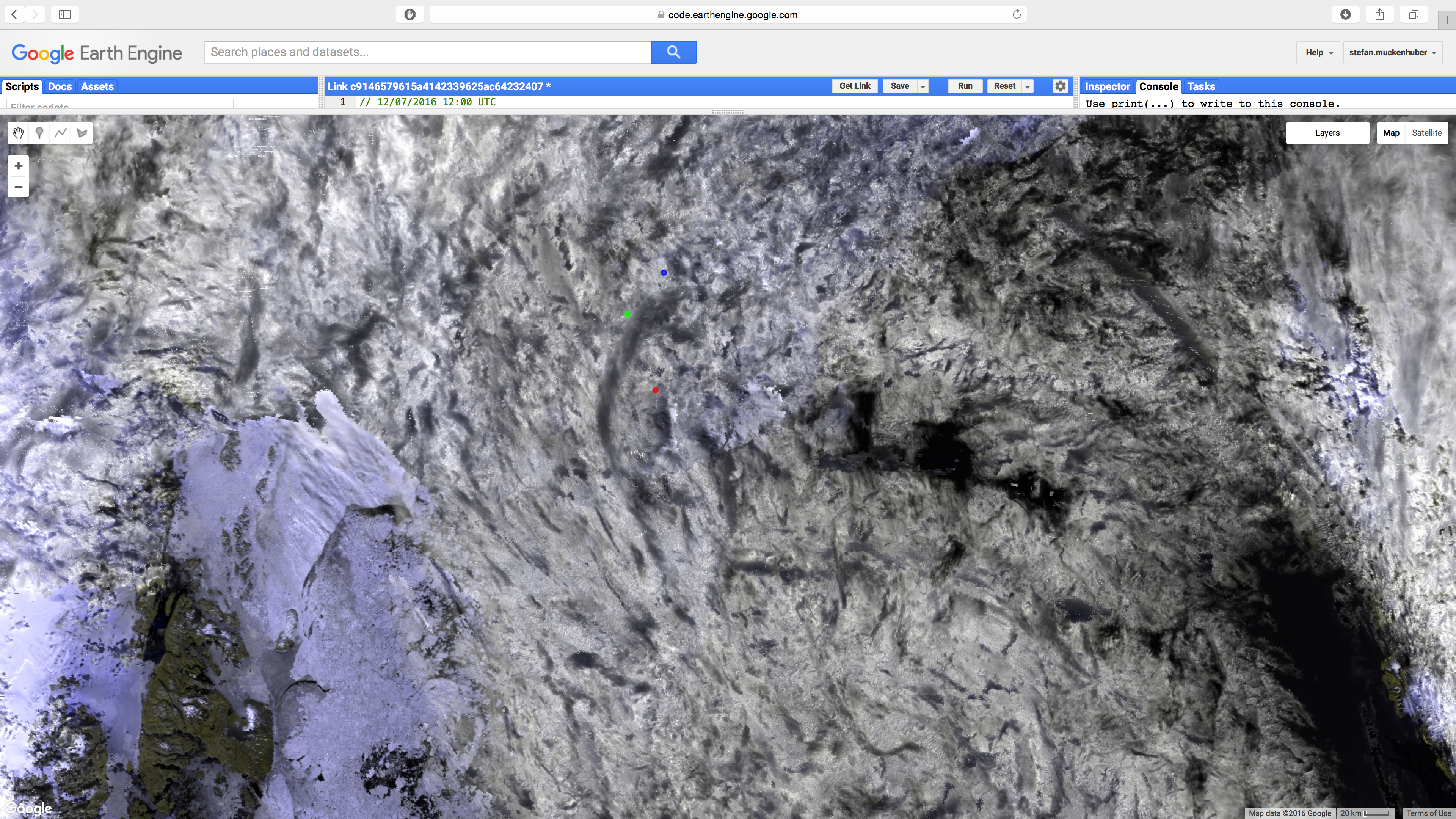

Supplement: Supplementary file 2 — Supplementary material [file mmc2.zip › GPS_tracker_data_python_plots_satellite/GPS_tracker_sat_data/MODIS_EE/MODIS_20160712.png]

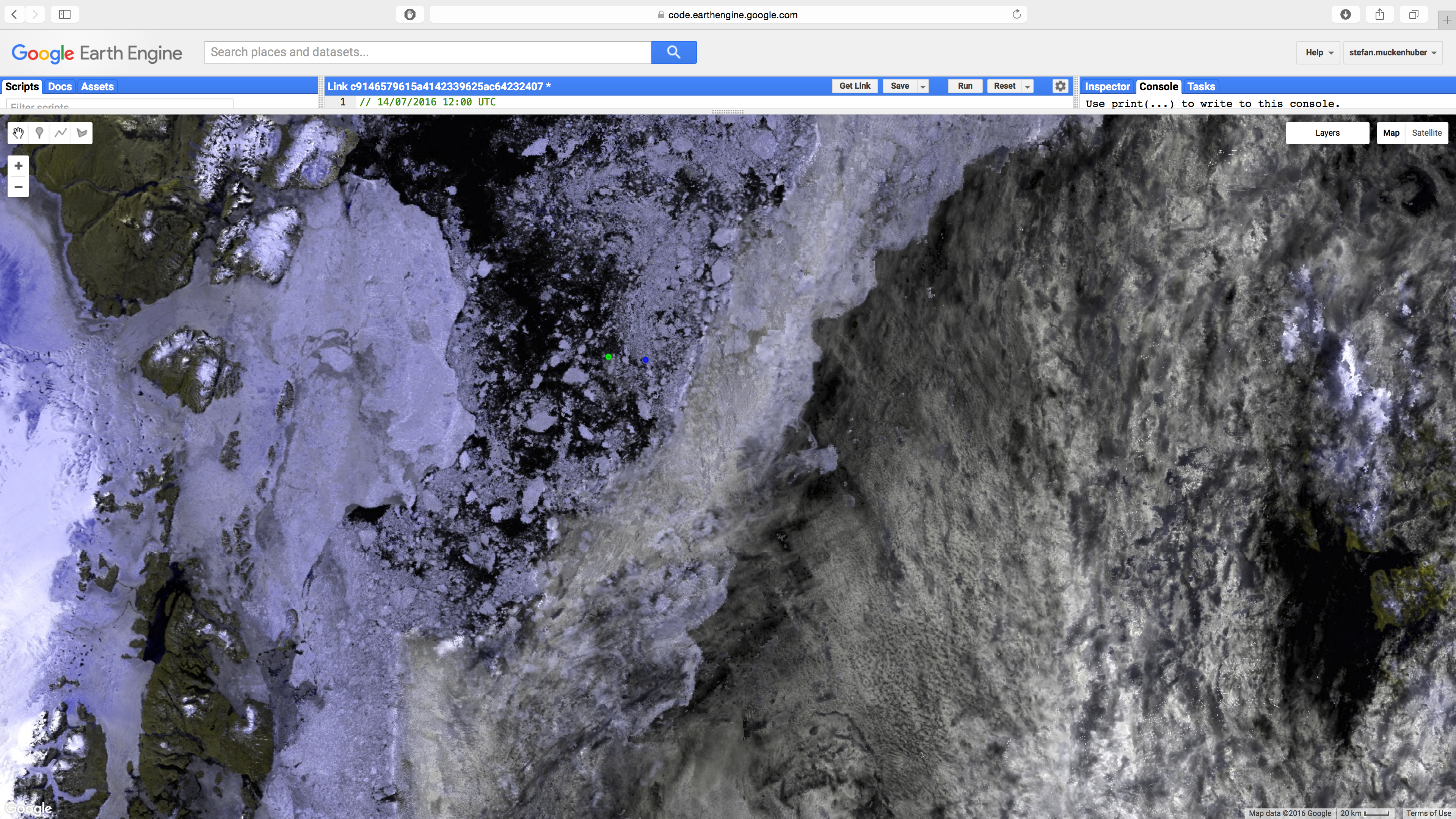

Supplement: Supplementary file 2 — Supplementary material [file mmc2.zip › GPS_tracker_data_python_plots_satellite/GPS_tracker_sat_data/MODIS_EE/MODIS_20160714.png]

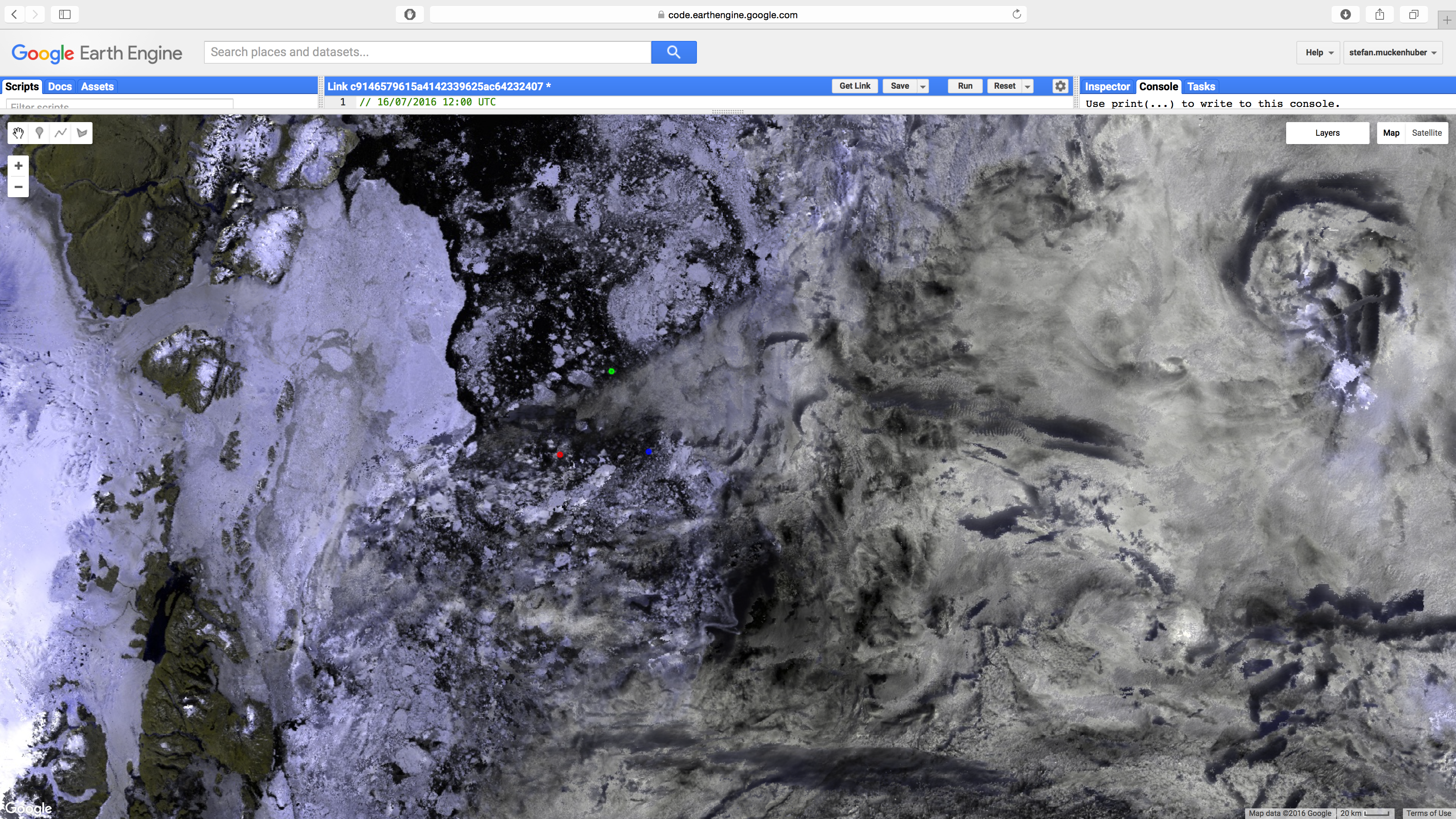

Supplement: Supplementary file 2 — Supplementary material [file mmc2.zip › GPS_tracker_data_python_plots_satellite/GPS_tracker_sat_data/MODIS_EE/MODIS_20160716.png]

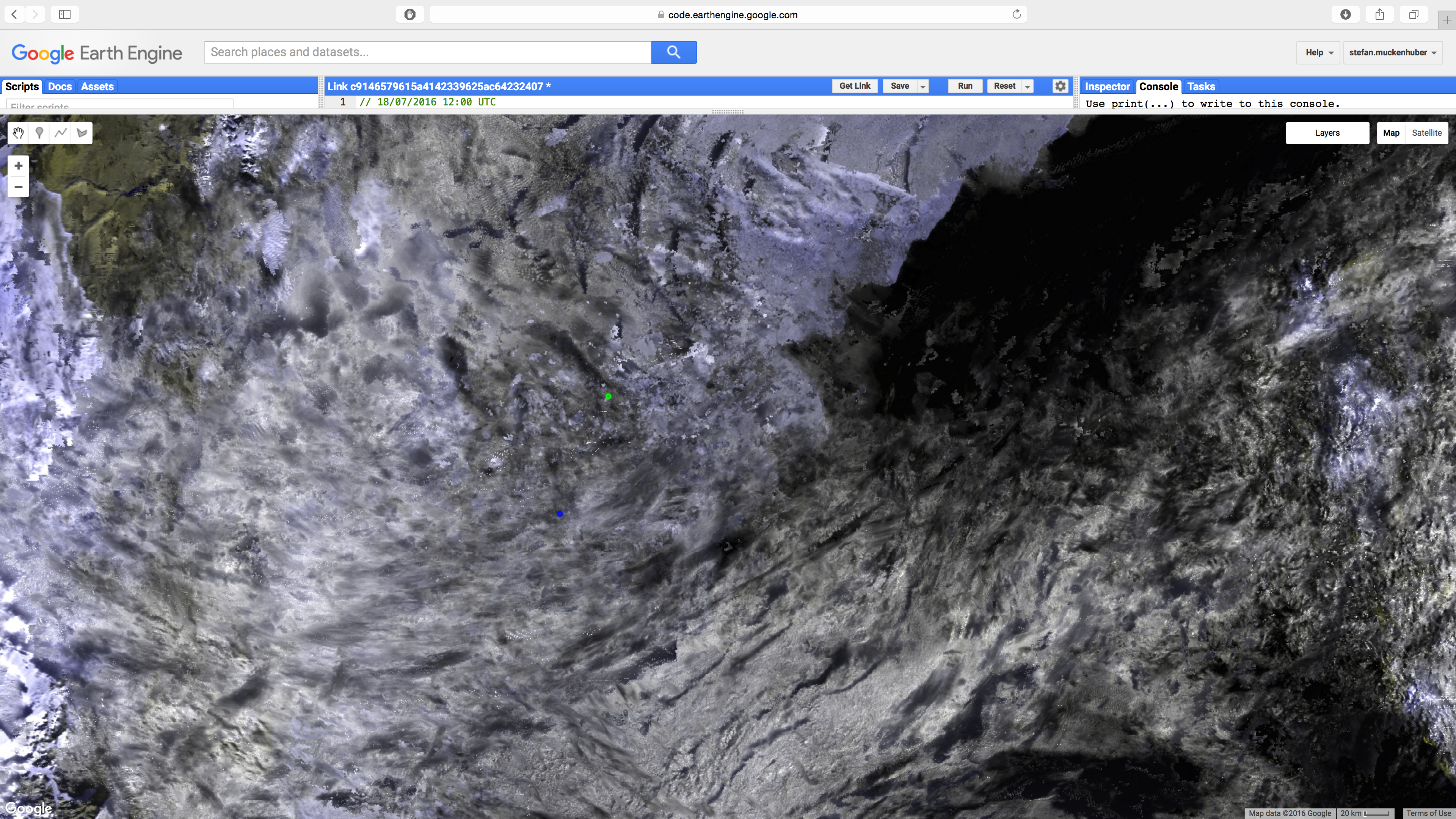

Supplement: Supplementary file 2 — Supplementary material [file mmc2.zip › GPS_tracker_data_python_plots_satellite/GPS_tracker_sat_data/MODIS_EE/MODIS_20160718.png]

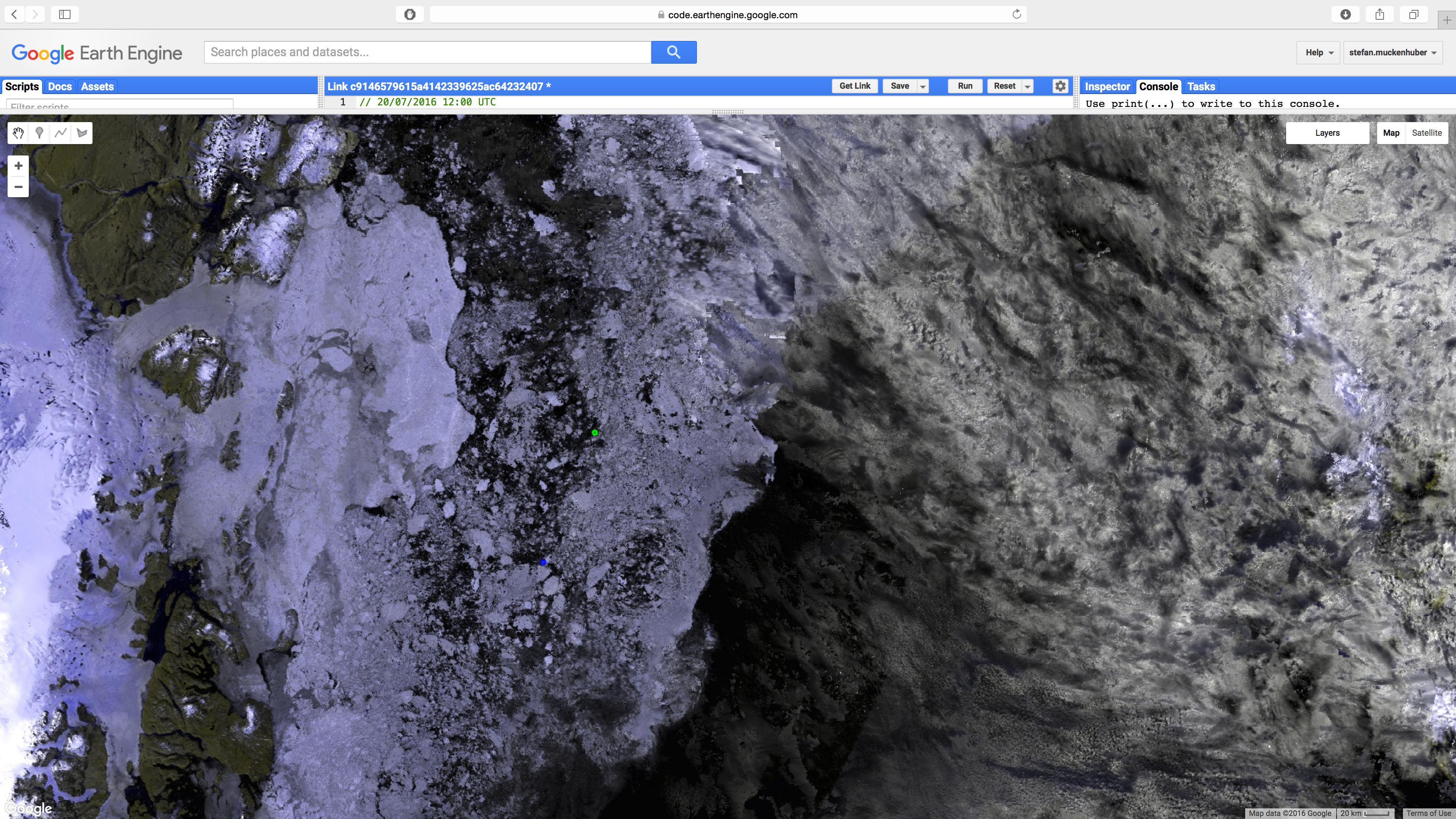

Supplement: Supplementary file 2 — Supplementary material [file mmc2.zip › GPS_tracker_data_python_plots_satellite/GPS_tracker_sat_data/MODIS_EE/MODIS_20160720.png]

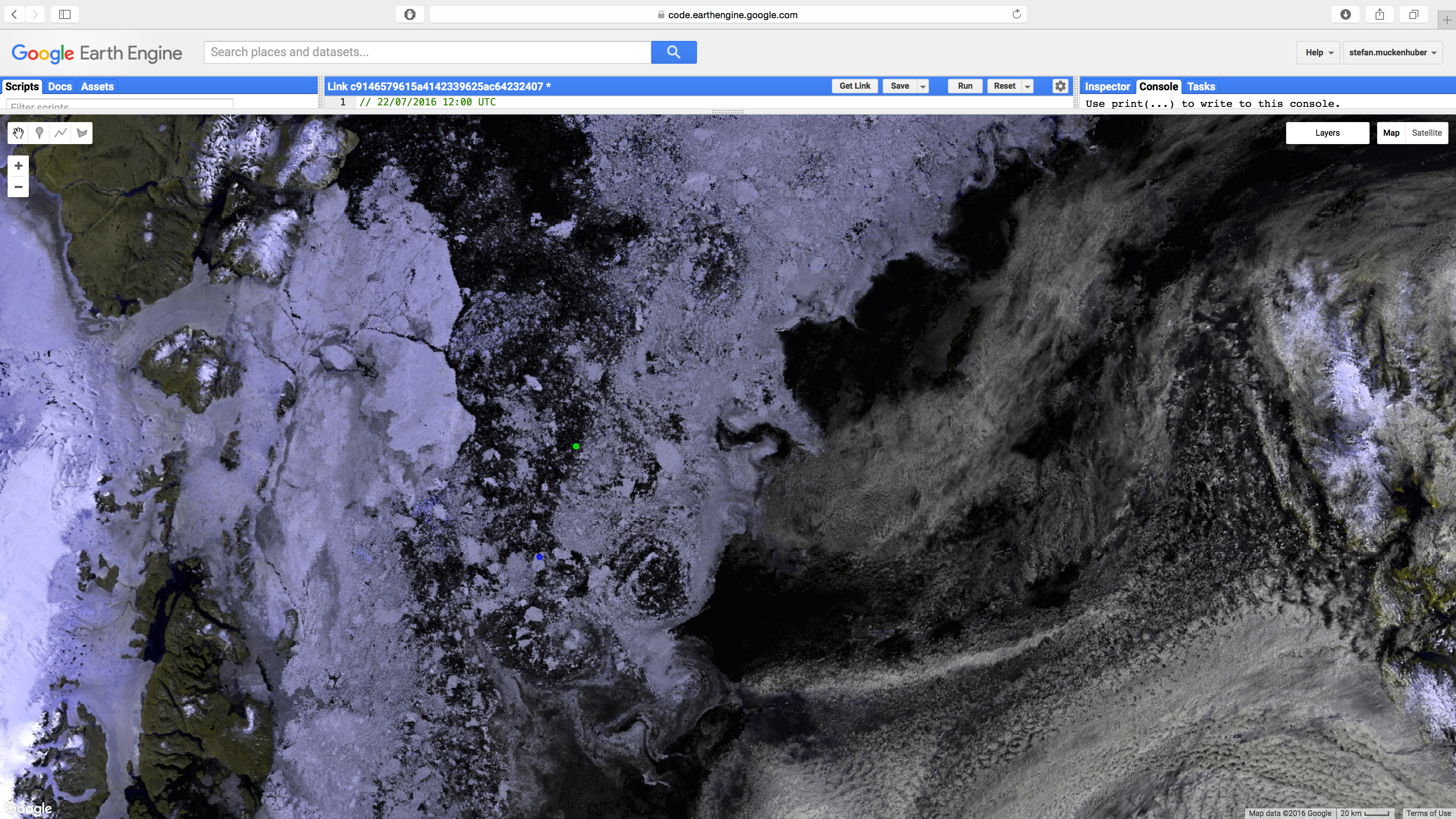

Supplement: Supplementary file 2 — Supplementary material [file mmc2.zip › GPS_tracker_data_python_plots_satellite/GPS_tracker_sat_data/MODIS_EE/MODIS_20160722.png]

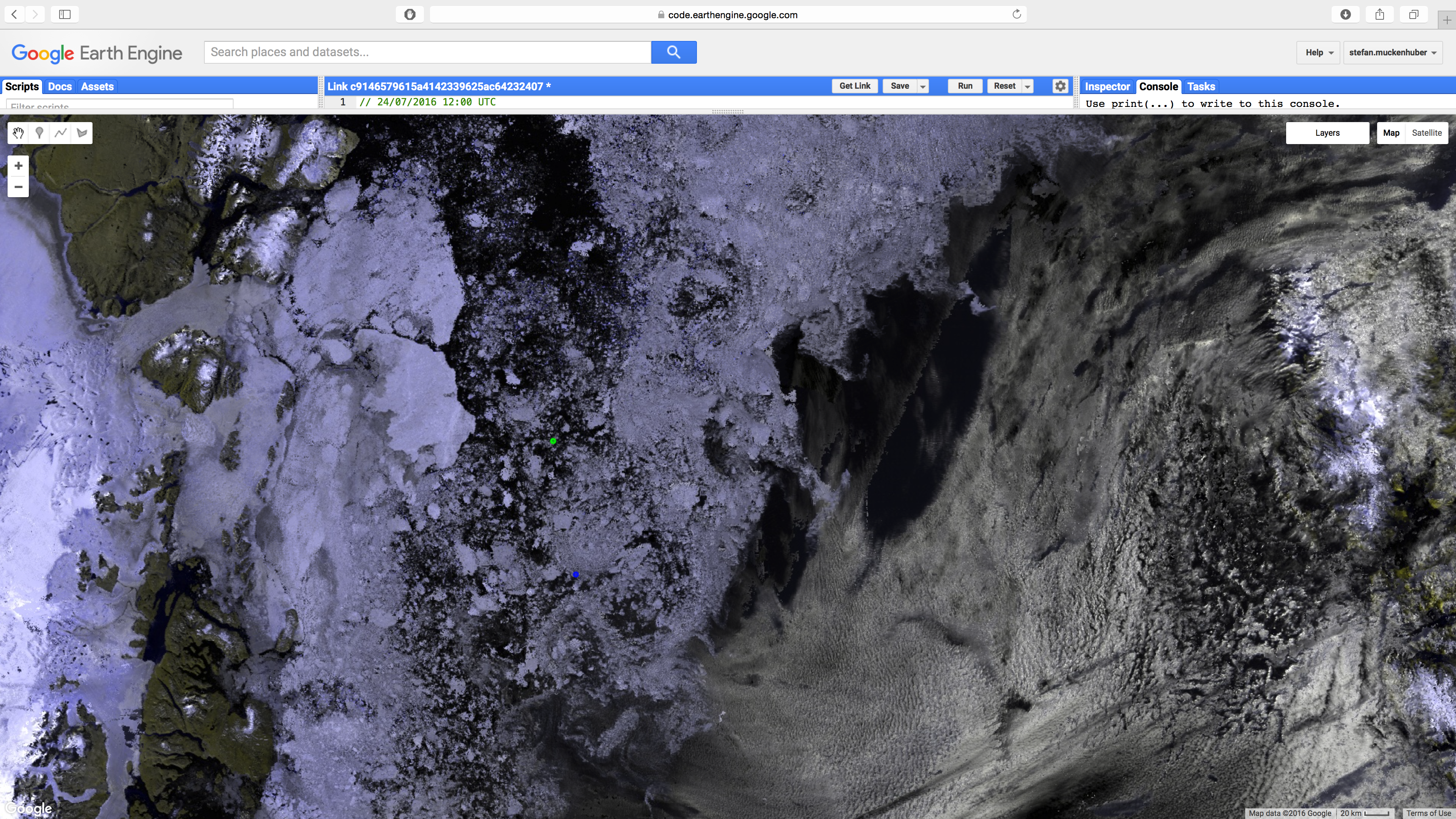

Supplement: Supplementary file 2 — Supplementary material [file mmc2.zip › GPS_tracker_data_python_plots_satellite/GPS_tracker_sat_data/MODIS_EE/MODIS_20160724.png]

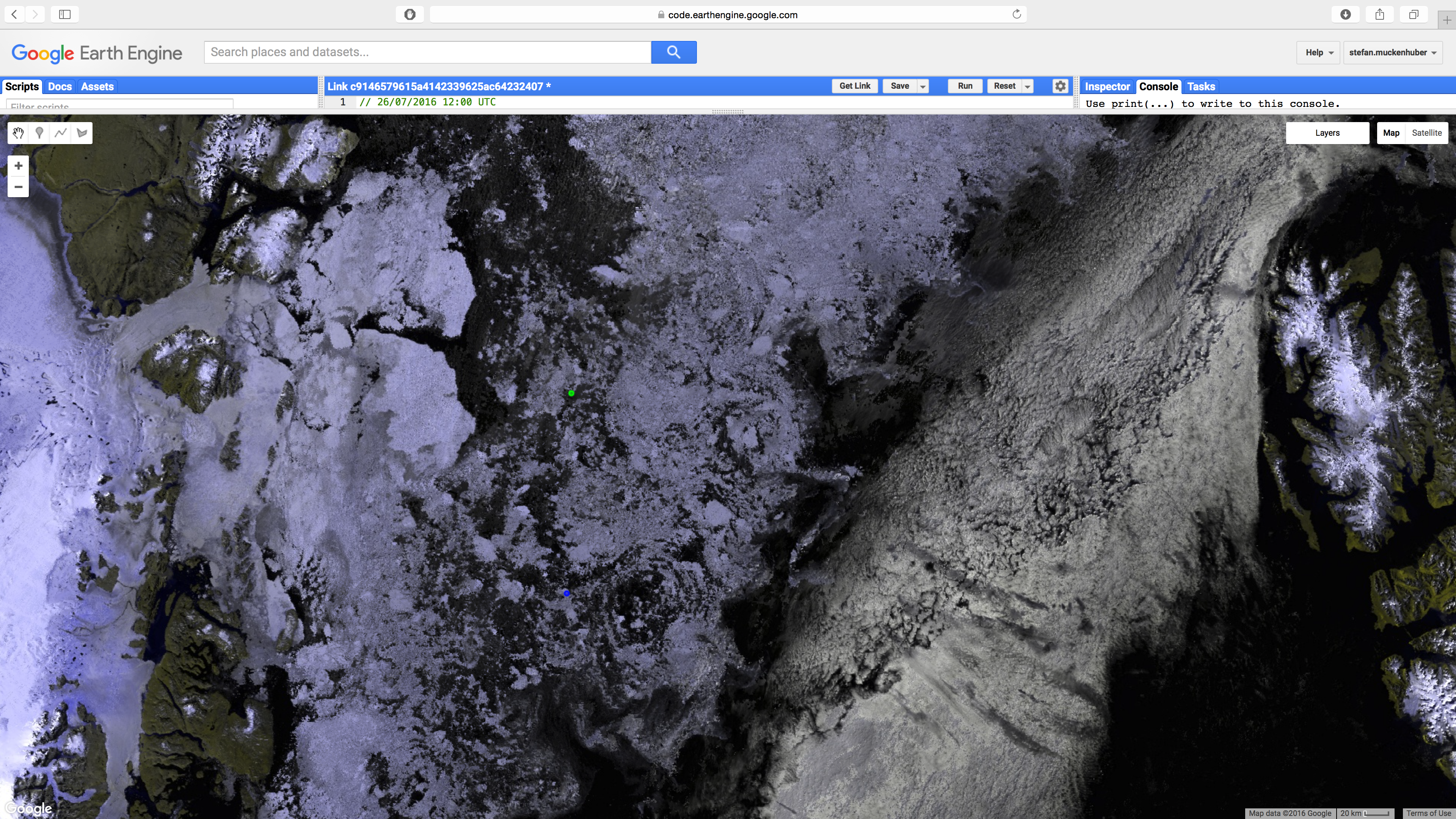

Supplement: Supplementary file 2 — Supplementary material [file mmc2.zip › GPS_tracker_data_python_plots_satellite/GPS_tracker_sat_data/MODIS_EE/MODIS_20160726.png]

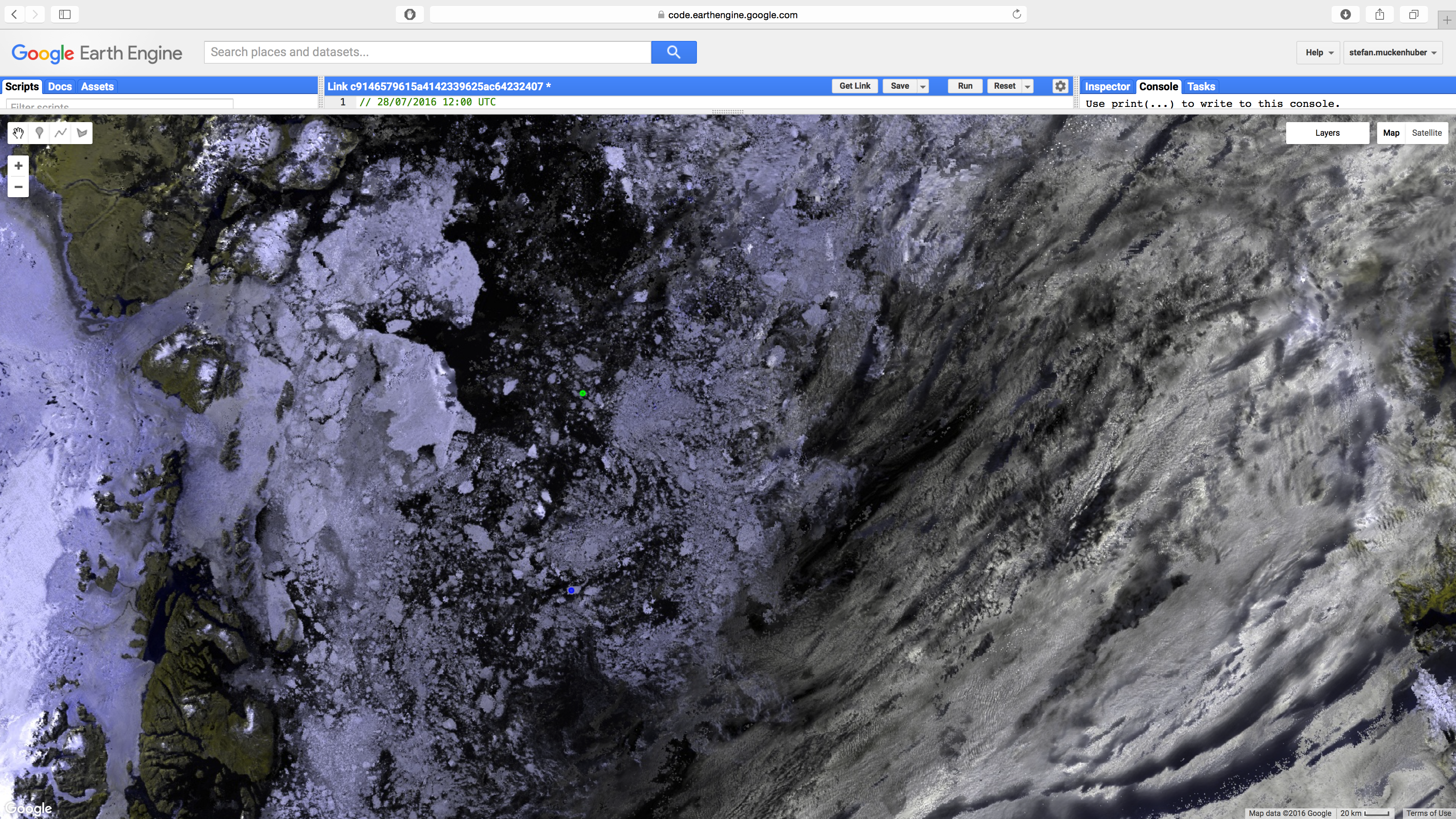

Supplement: Supplementary file 2 — Supplementary material [file mmc2.zip › GPS_tracker_data_python_plots_satellite/GPS_tracker_sat_data/MODIS_EE/MODIS_20160728.png]

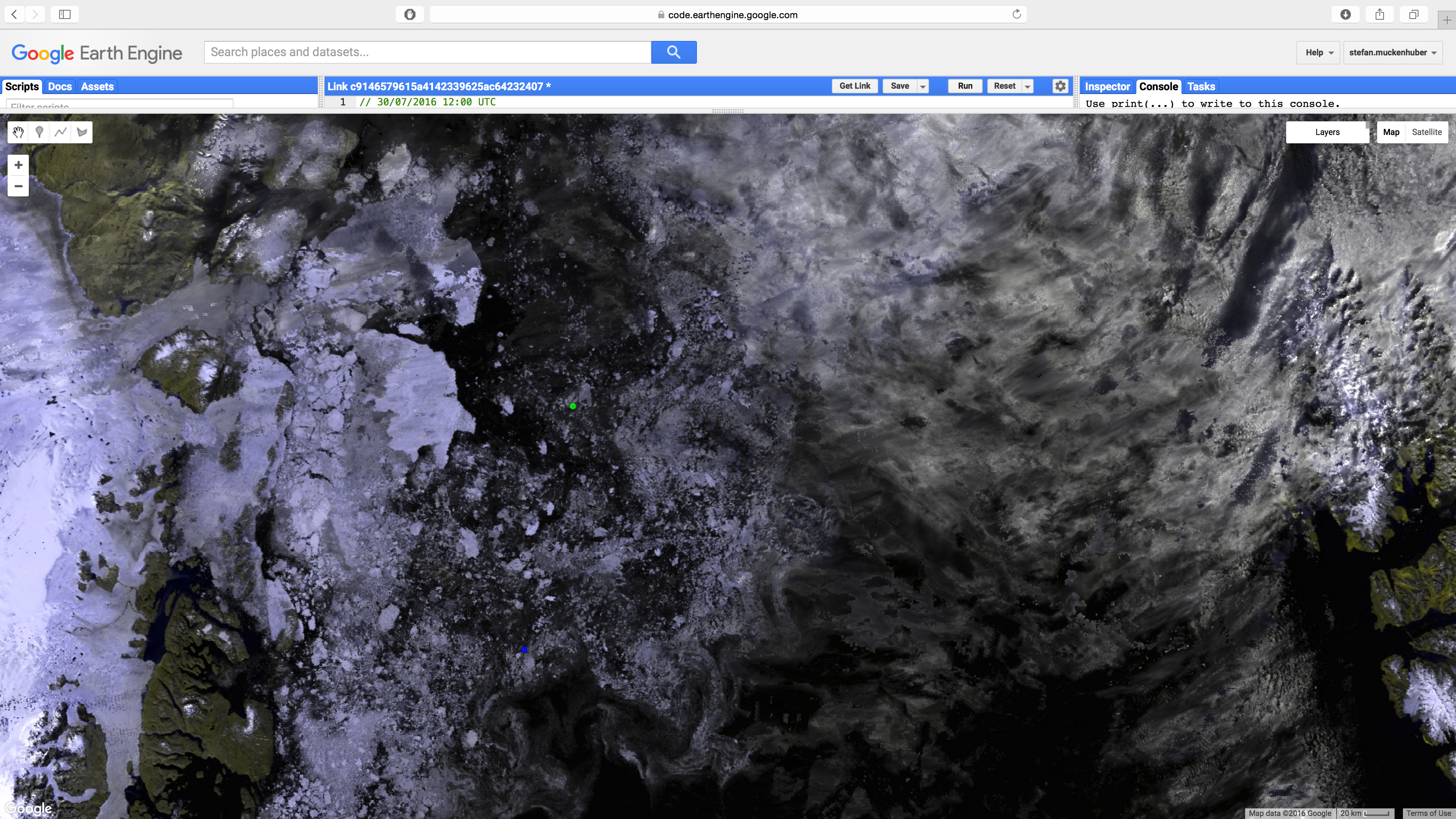

Supplement: Supplementary file 2 — Supplementary material [file mmc2.zip › GPS_tracker_data_python_plots_satellite/GPS_tracker_sat_data/MODIS_EE/MODIS_20160730.png]

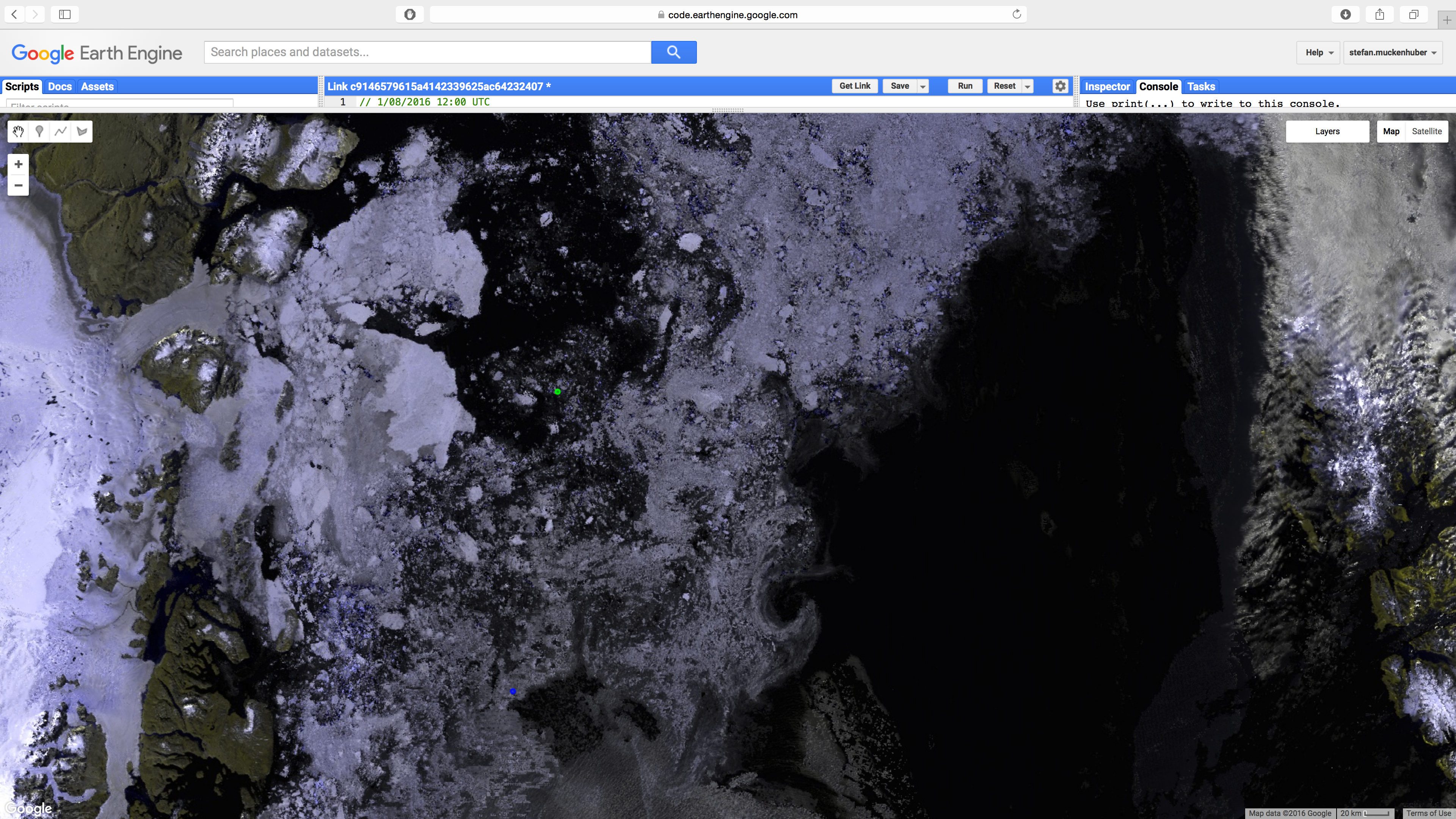

Supplement: Supplementary file 2 — Supplementary material [file mmc2.zip › GPS_tracker_data_python_plots_satellite/GPS_tracker_sat_data/MODIS_EE/MODIS_20160801.png]

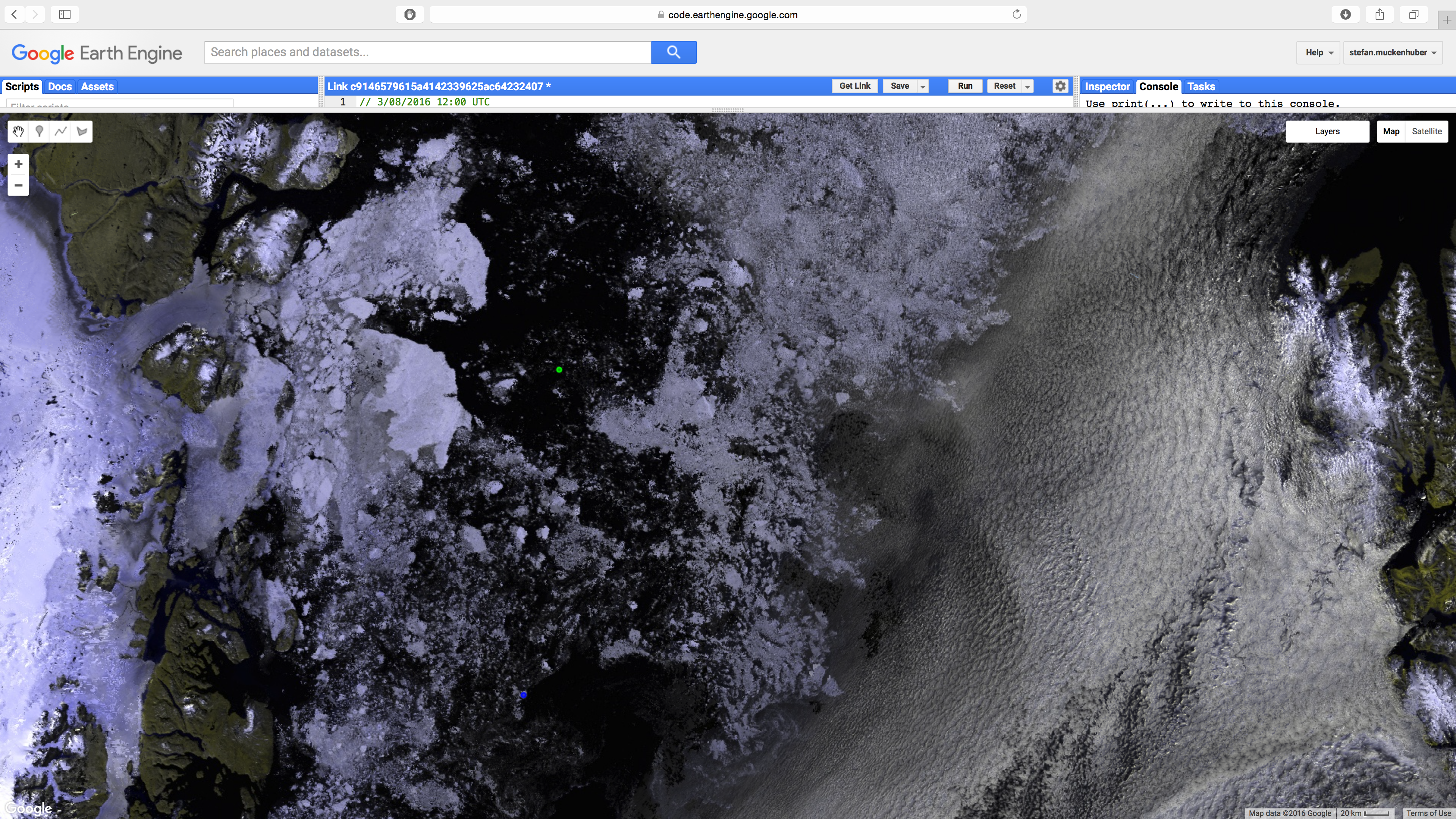

Supplement: Supplementary file 2 — Supplementary material [file mmc2.zip › GPS_tracker_data_python_plots_satellite/GPS_tracker_sat_data/MODIS_EE/MODIS_20160803.png]

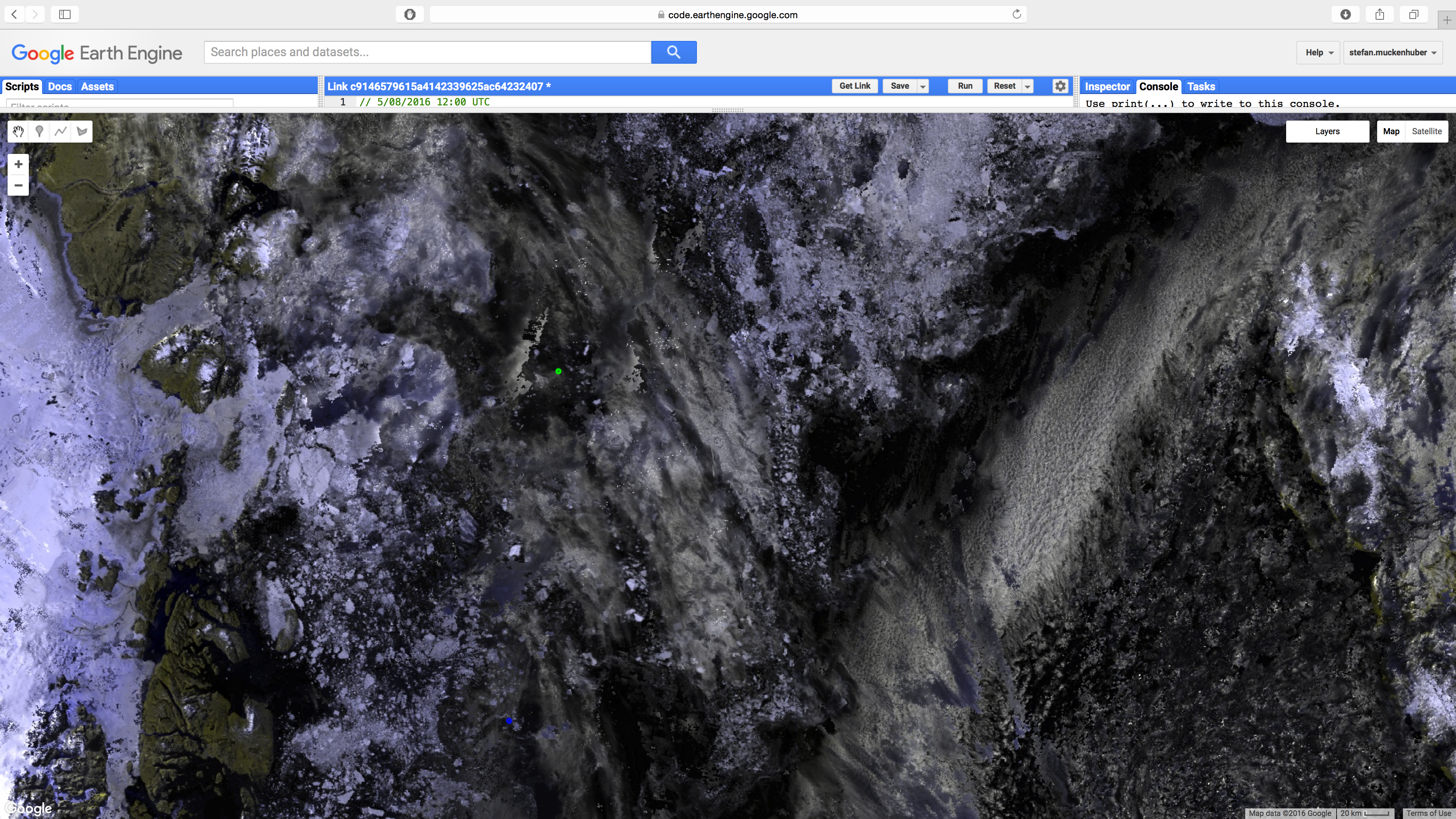

Supplement: Supplementary file 2 — Supplementary material [file mmc2.zip › GPS_tracker_data_python_plots_satellite/GPS_tracker_sat_data/MODIS_EE/MODIS_20160805.png]

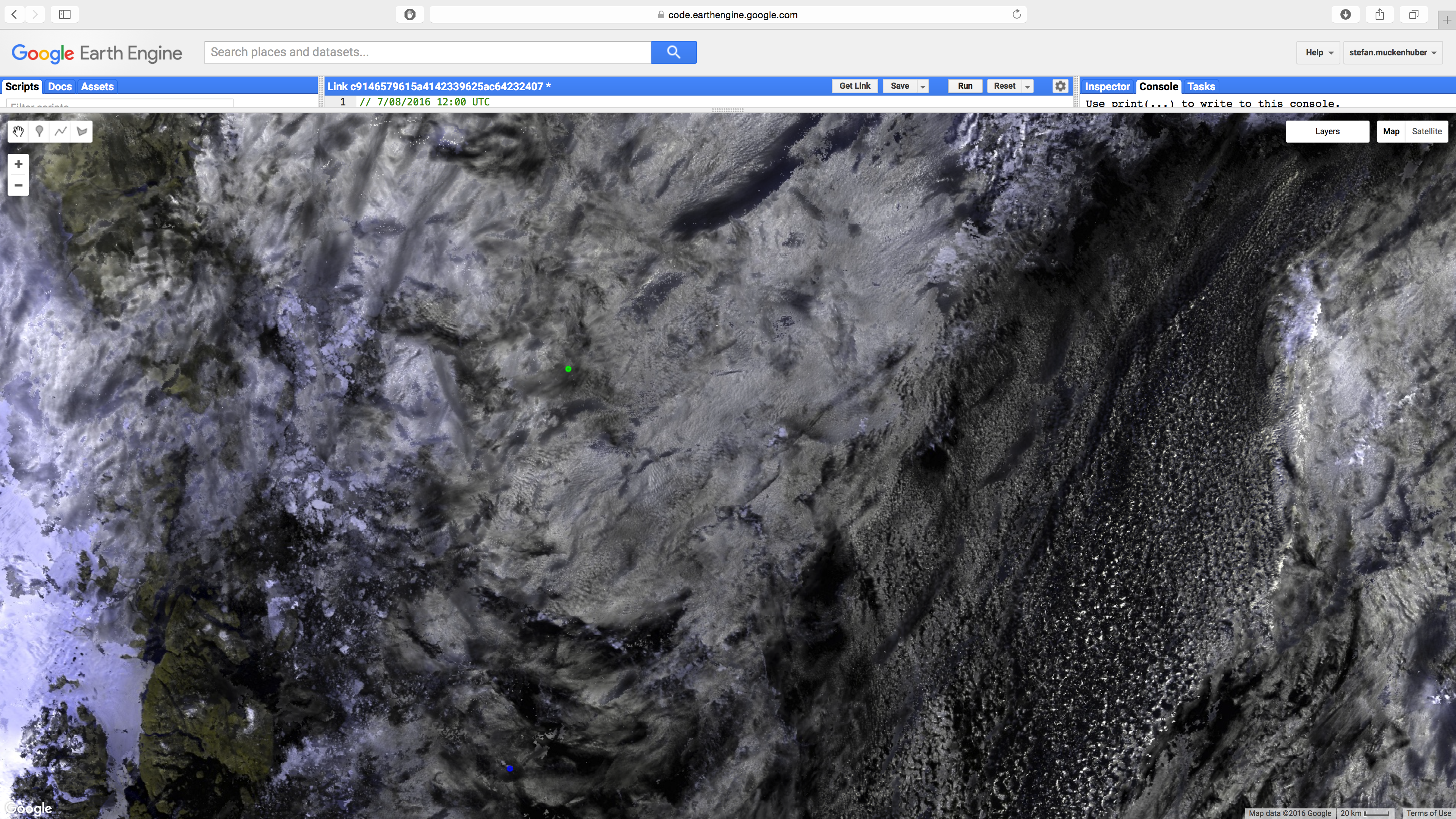

Supplement: Supplementary file 2 — Supplementary material [file mmc2.zip › GPS_tracker_data_python_plots_satellite/GPS_tracker_sat_data/MODIS_EE/MODIS_20160807.png]

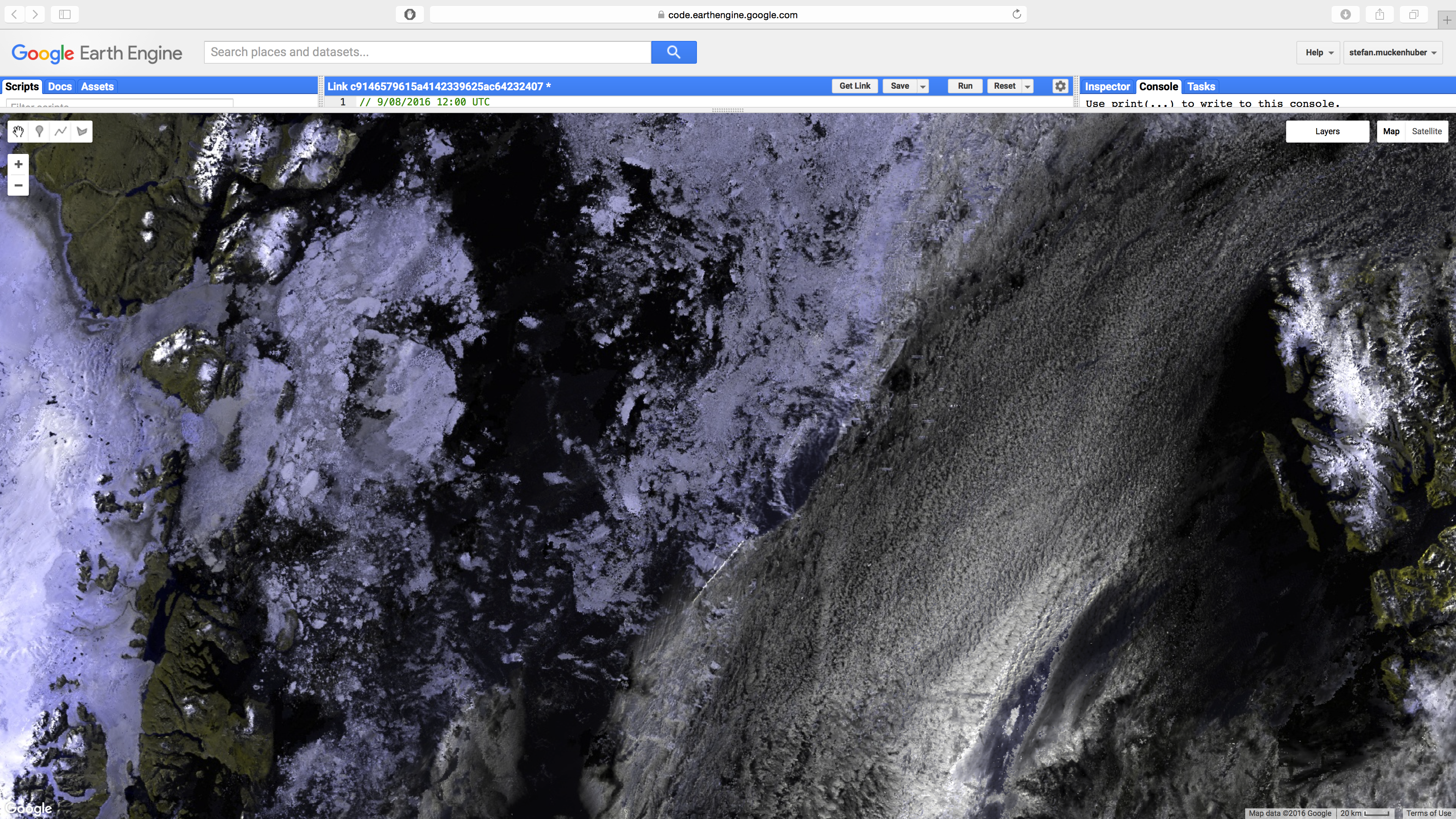

Supplement: Supplementary file 2 — Supplementary material [file mmc2.zip › GPS_tracker_data_python_plots_satellite/GPS_tracker_sat_data/MODIS_EE/MODIS_20160809.png]

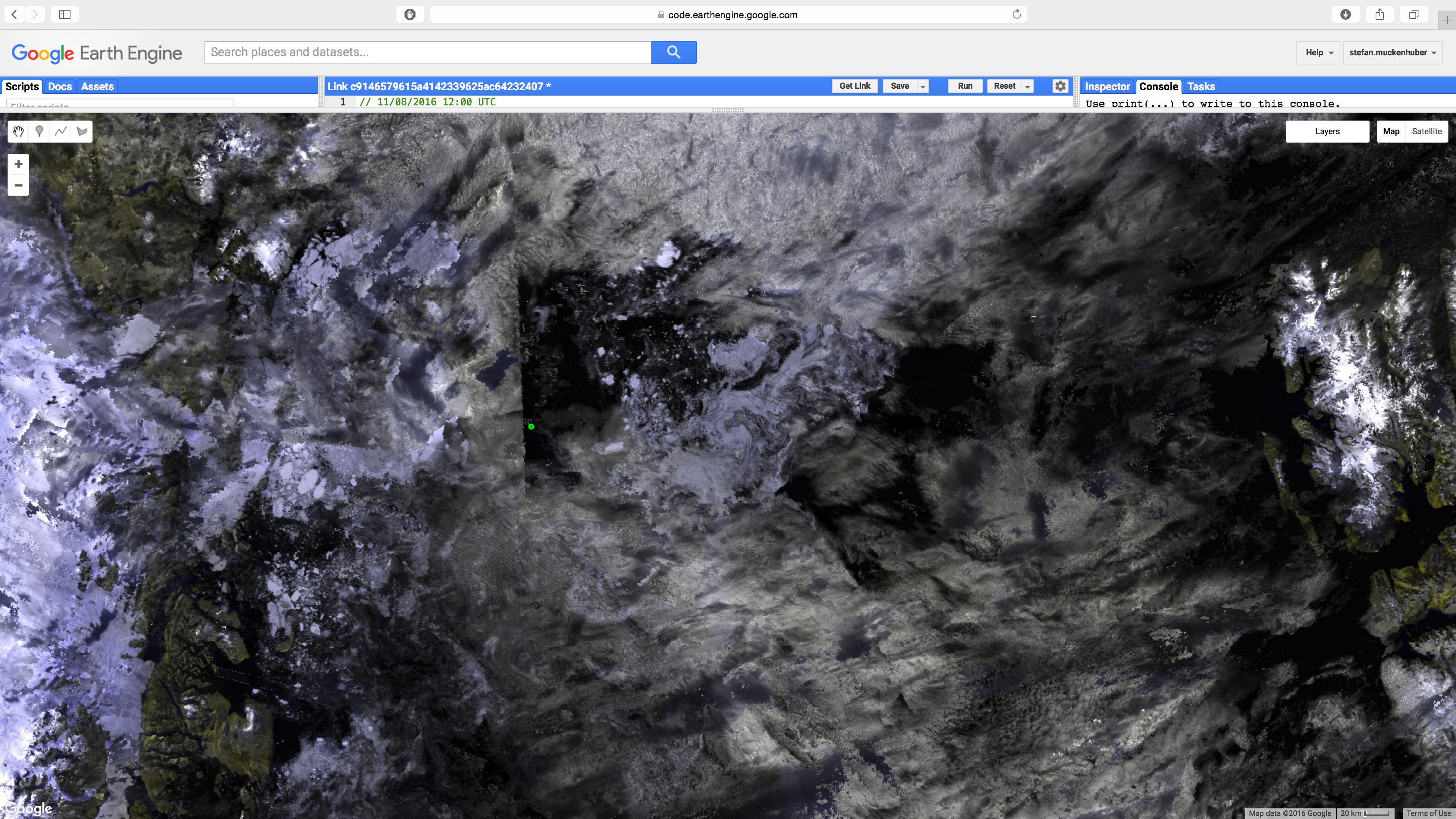

Supplement: Supplementary file 2 — Supplementary material [file mmc2.zip › GPS_tracker_data_python_plots_satellite/GPS_tracker_sat_data/MODIS_EE/MODIS_20160811.png]

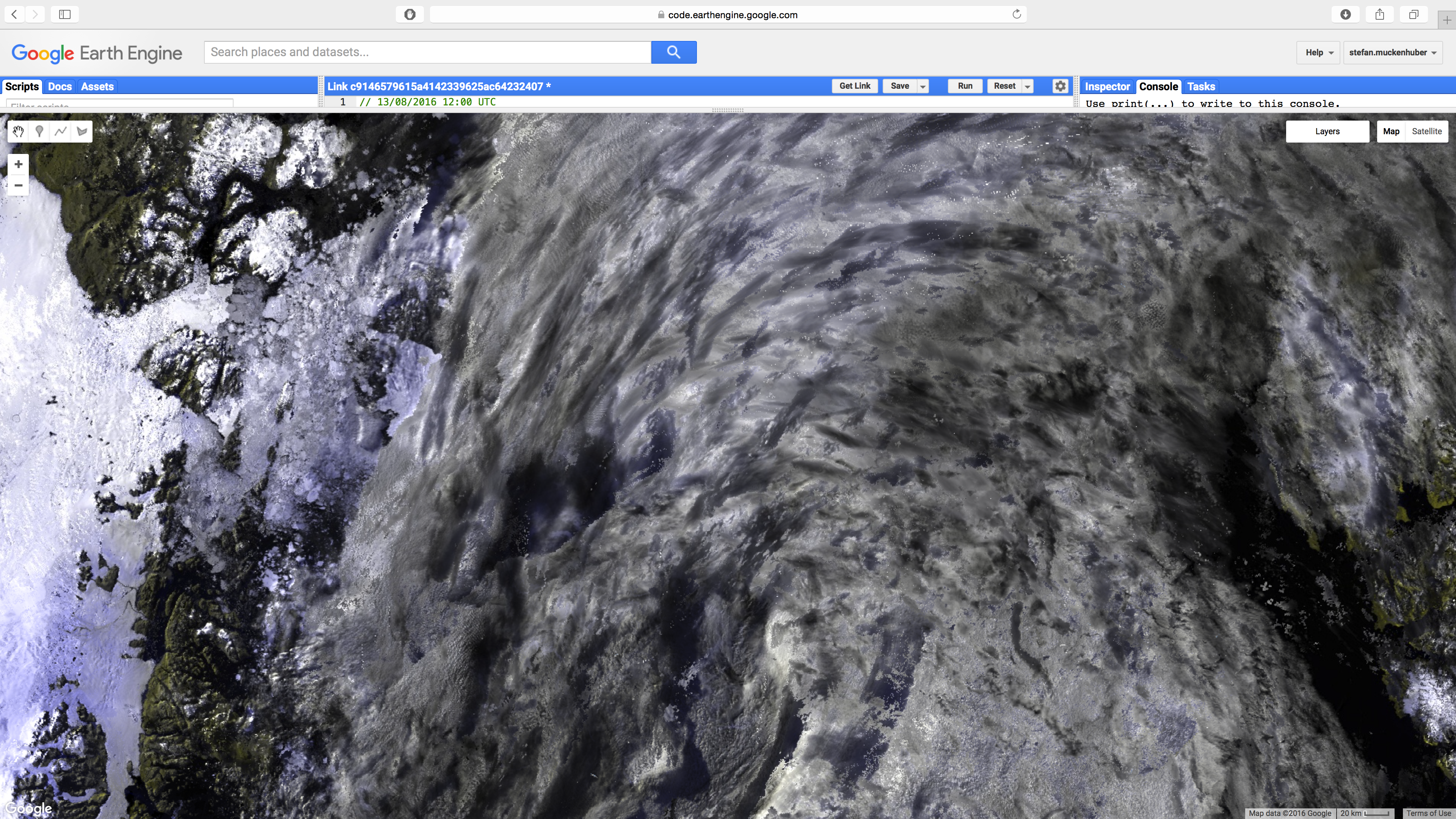

Supplement: Supplementary file 2 — Supplementary material [file mmc2.zip › GPS_tracker_data_python_plots_satellite/GPS_tracker_sat_data/MODIS_EE/MODIS_20160813.png]

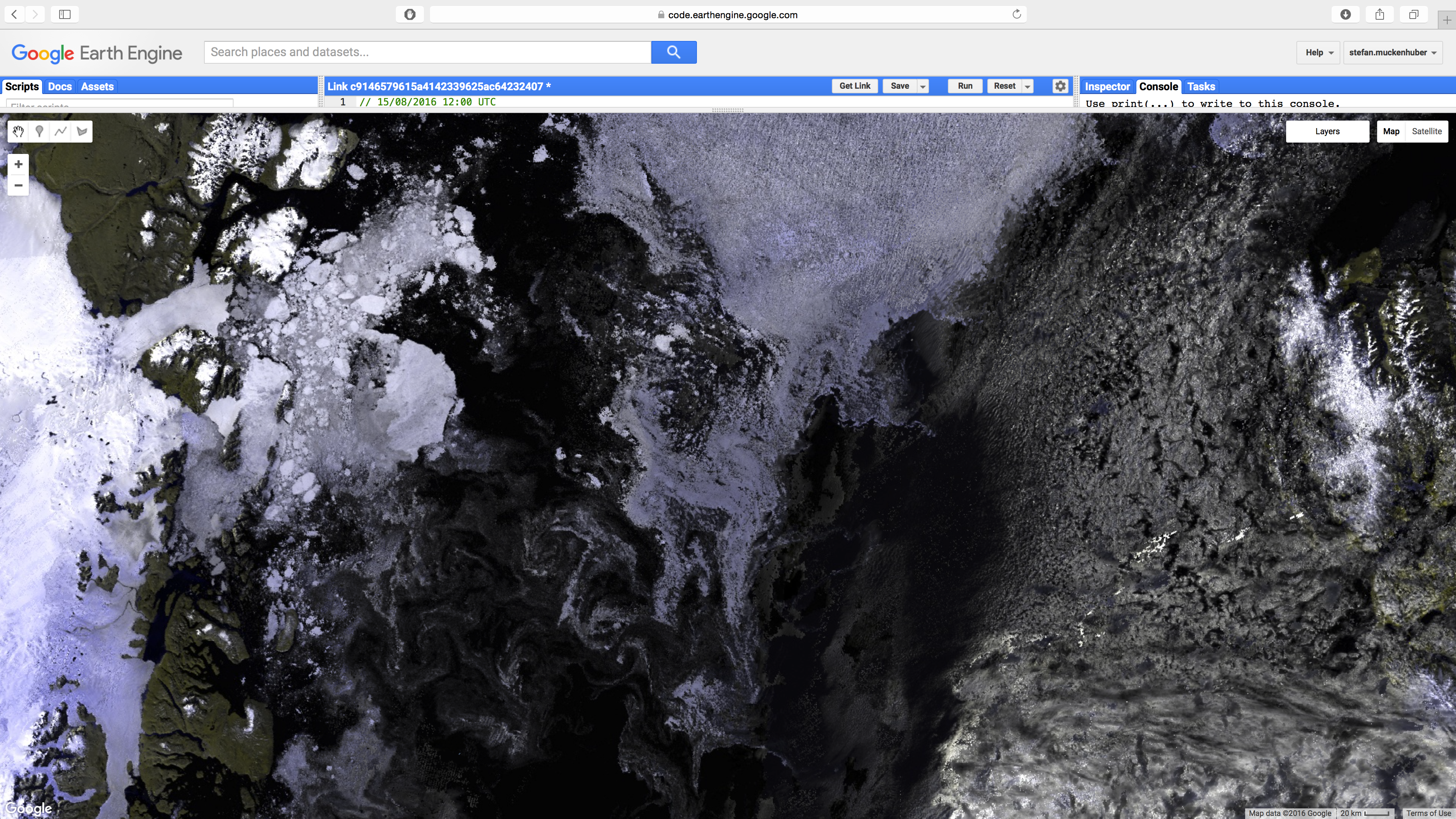

Supplement: Supplementary file 2 — Supplementary material [file mmc2.zip › GPS_tracker_data_python_plots_satellite/GPS_tracker_sat_data/MODIS_EE/MODIS_20160815.png]

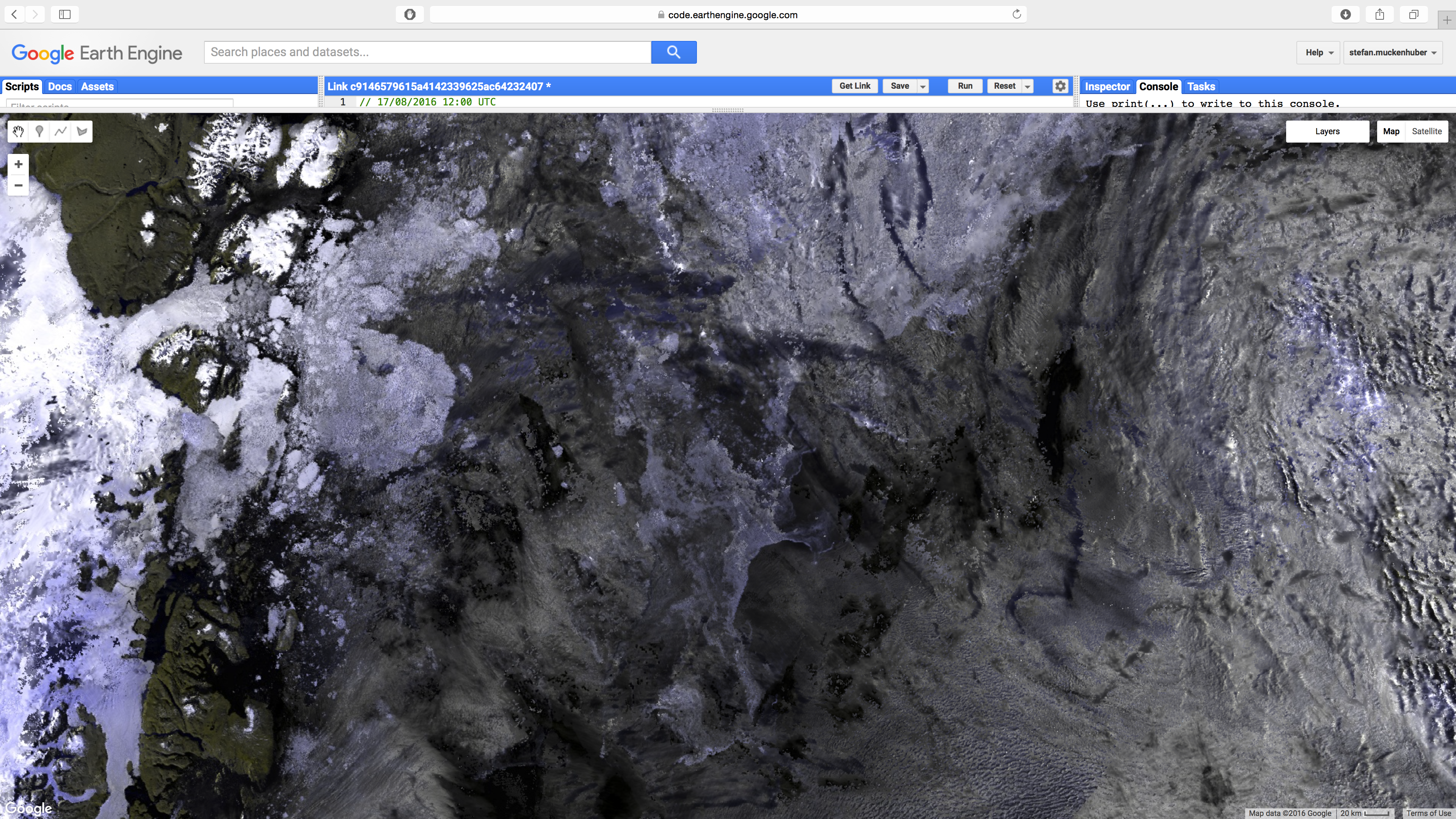

Supplement: Supplementary file 2 — Supplementary material [file mmc2.zip › GPS_tracker_data_python_plots_satellite/GPS_tracker_sat_data/MODIS_EE/MODIS_20160817.png]

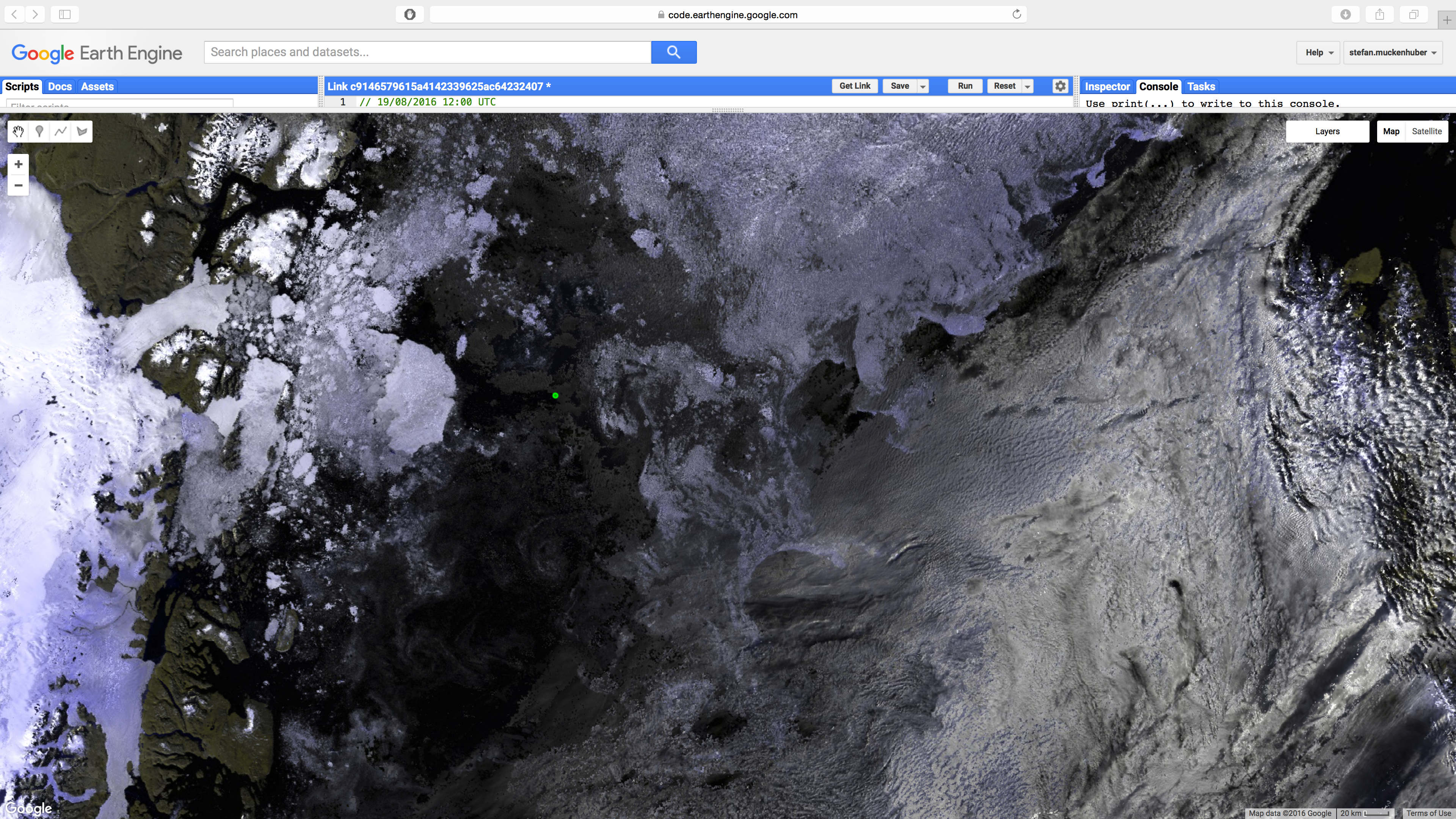

Supplement: Supplementary file 2 — Supplementary material [file mmc2.zip › GPS_tracker_data_python_plots_satellite/GPS_tracker_sat_data/MODIS_EE/MODIS_20160819.png]

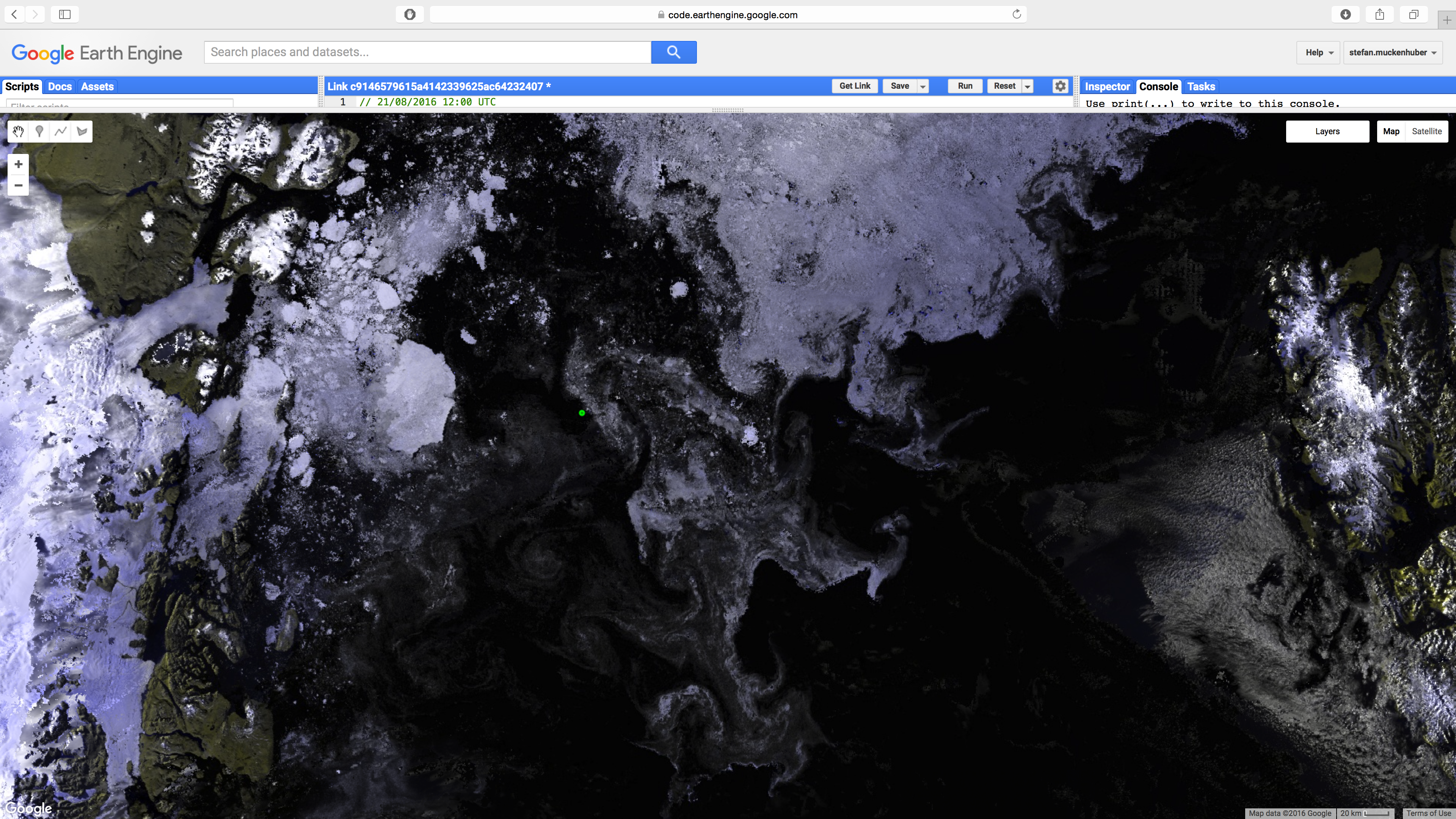

Supplement: Supplementary file 2 — Supplementary material [file mmc2.zip › GPS_tracker_data_python_plots_satellite/GPS_tracker_sat_data/MODIS_EE/MODIS_20160821.png]

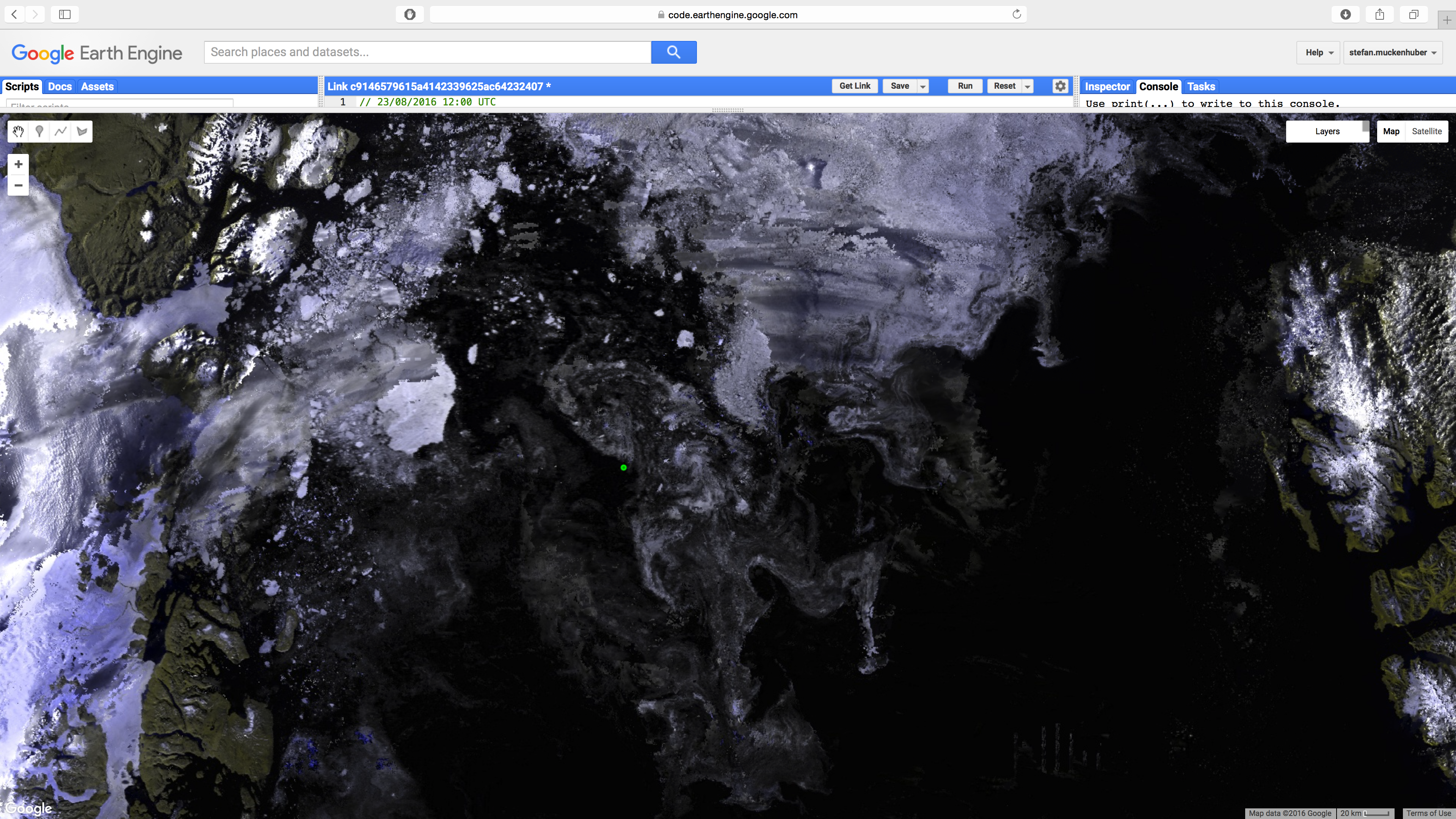

Supplement: Supplementary file 2 — Supplementary material [file mmc2.zip › GPS_tracker_data_python_plots_satellite/GPS_tracker_sat_data/MODIS_EE/MODIS_20160823.png]

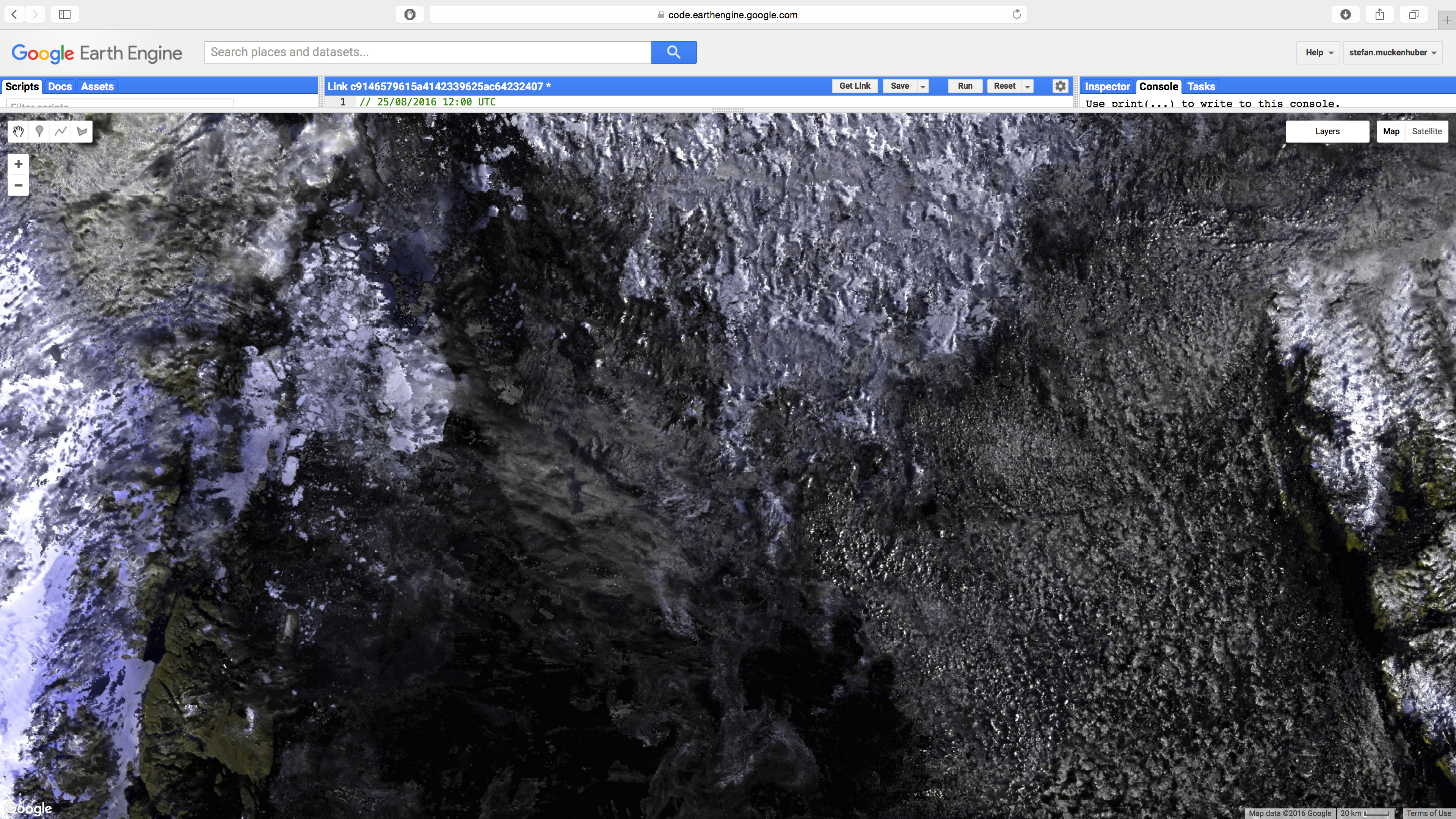

Supplement: Supplementary file 2 — Supplementary material [file mmc2.zip › GPS_tracker_data_python_plots_satellite/GPS_tracker_sat_data/MODIS_EE/MODIS_20160825.png]

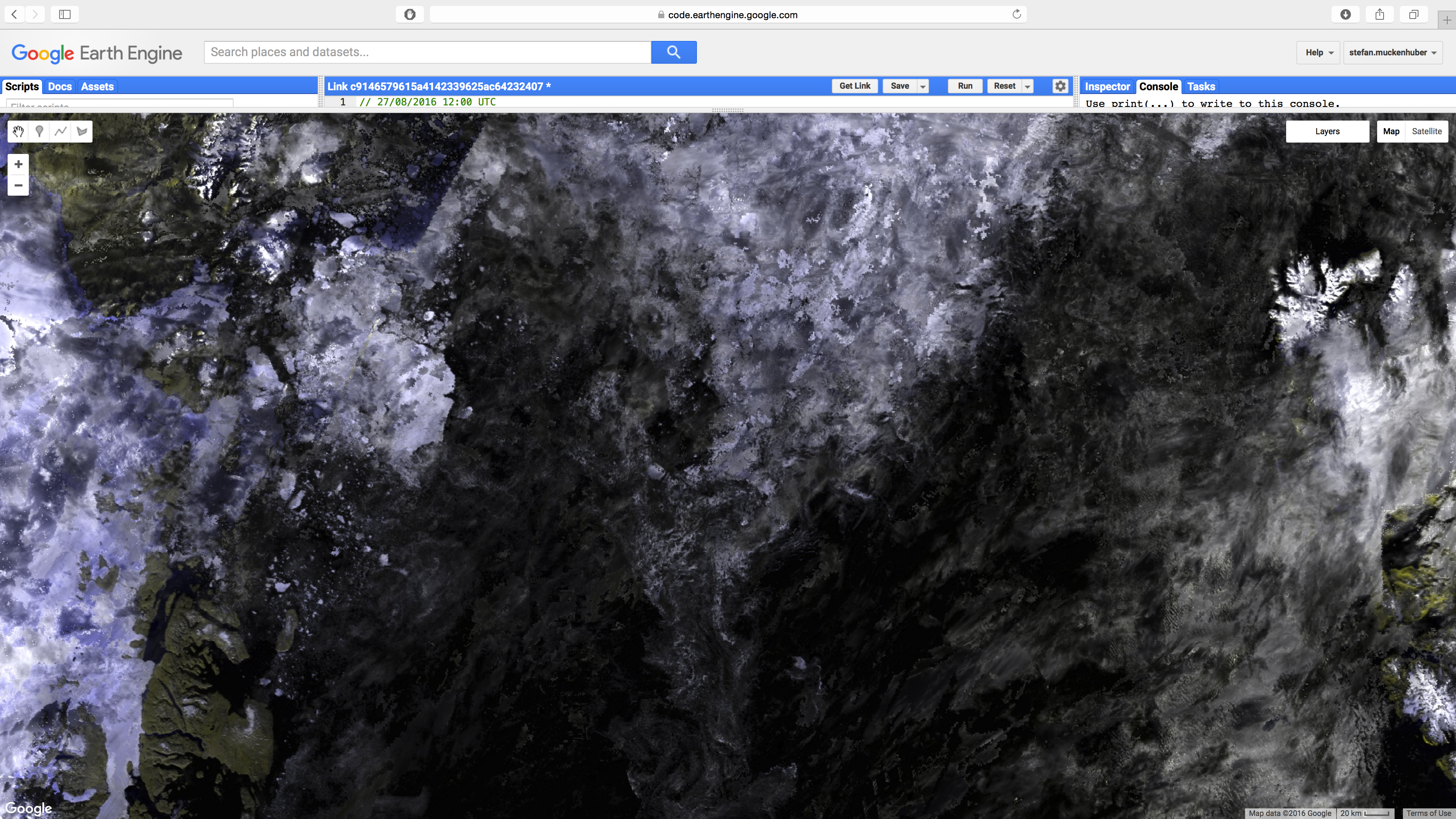

Supplement: Supplementary file 2 — Supplementary material [file mmc2.zip › GPS_tracker_data_python_plots_satellite/GPS_tracker_sat_data/MODIS_EE/MODIS_20160827.png]

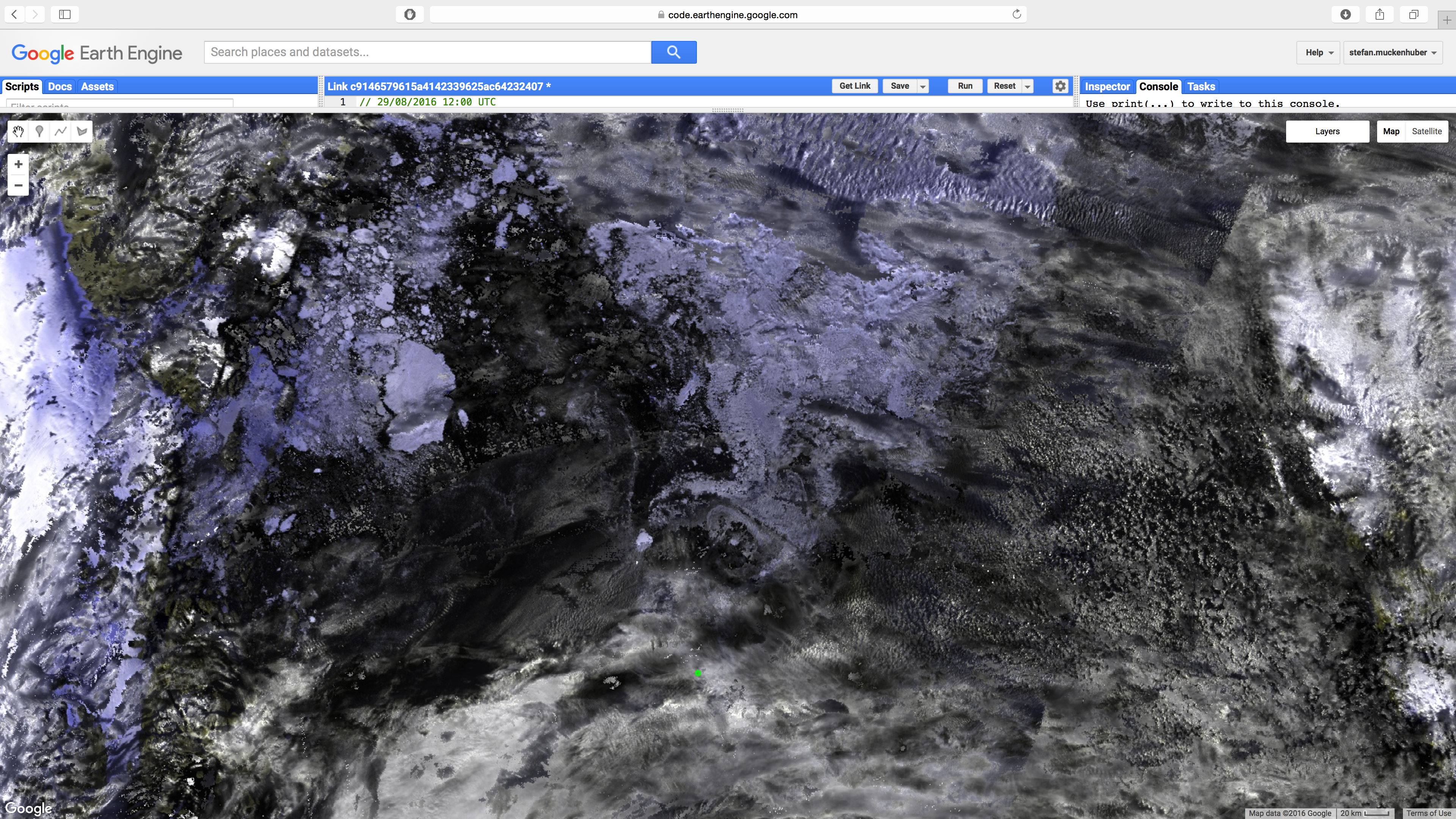

Supplement: Supplementary file 2 — Supplementary material [file mmc2.zip › GPS_tracker_data_python_plots_satellite/GPS_tracker_sat_data/MODIS_EE/MODIS_20160829.png]

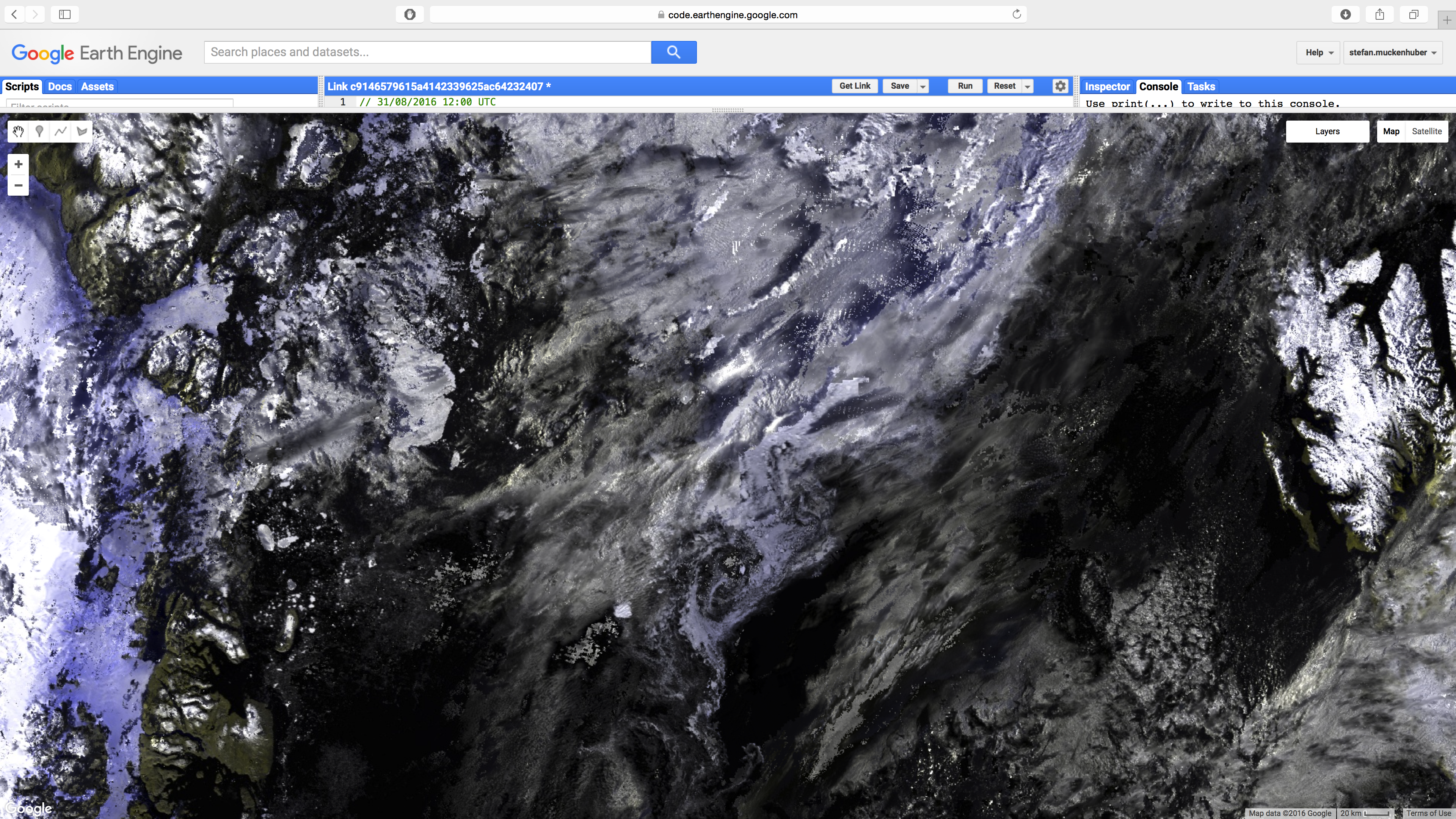

Supplement: Supplementary file 2 — Supplementary material [file mmc2.zip › GPS_tracker_data_python_plots_satellite/GPS_tracker_sat_data/MODIS_EE/MODIS_20160831.png]

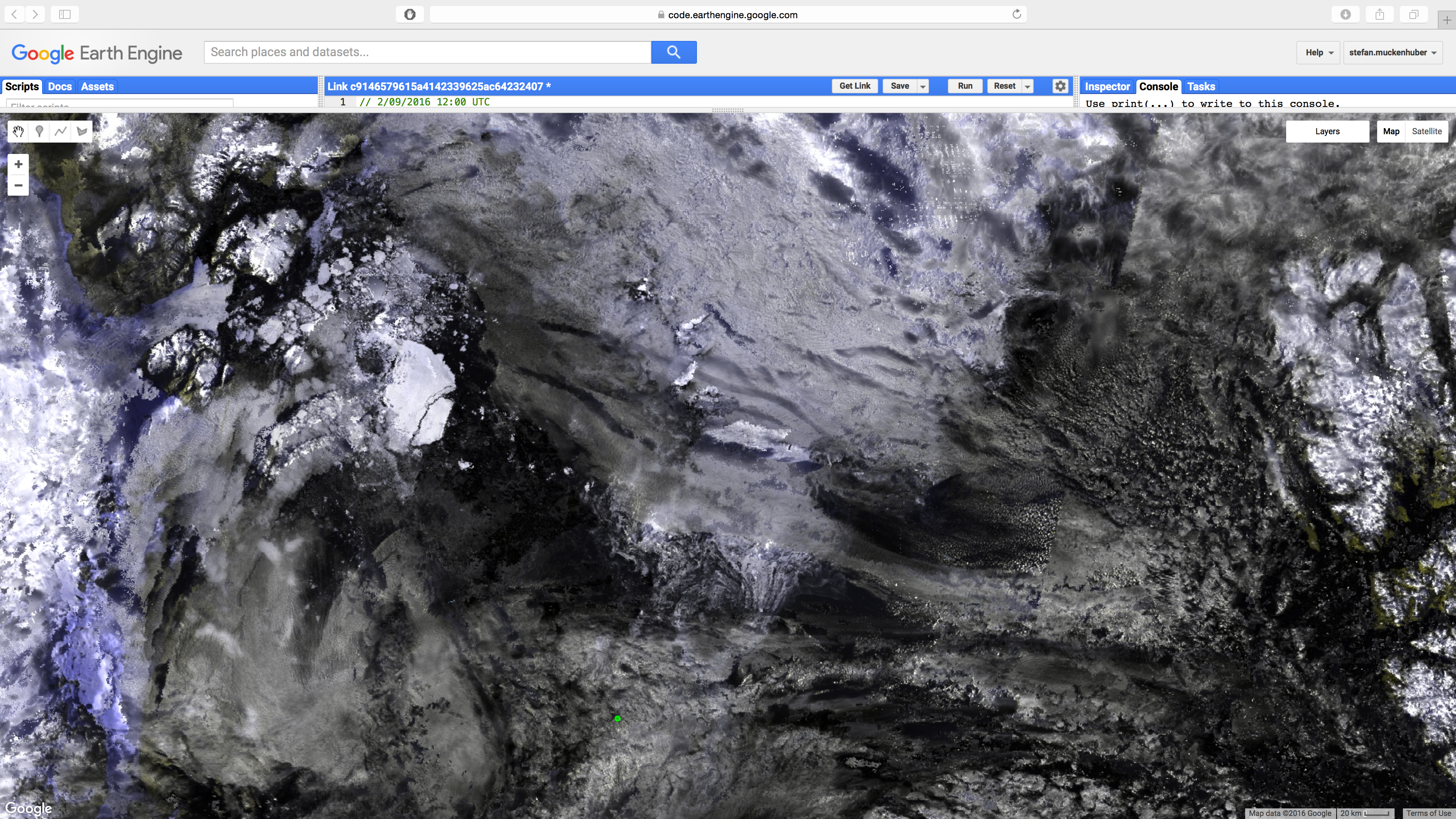

Supplement: Supplementary file 2 — Supplementary material [file mmc2.zip › GPS_tracker_data_python_plots_satellite/GPS_tracker_sat_data/MODIS_EE/MODIS_20160902.png]

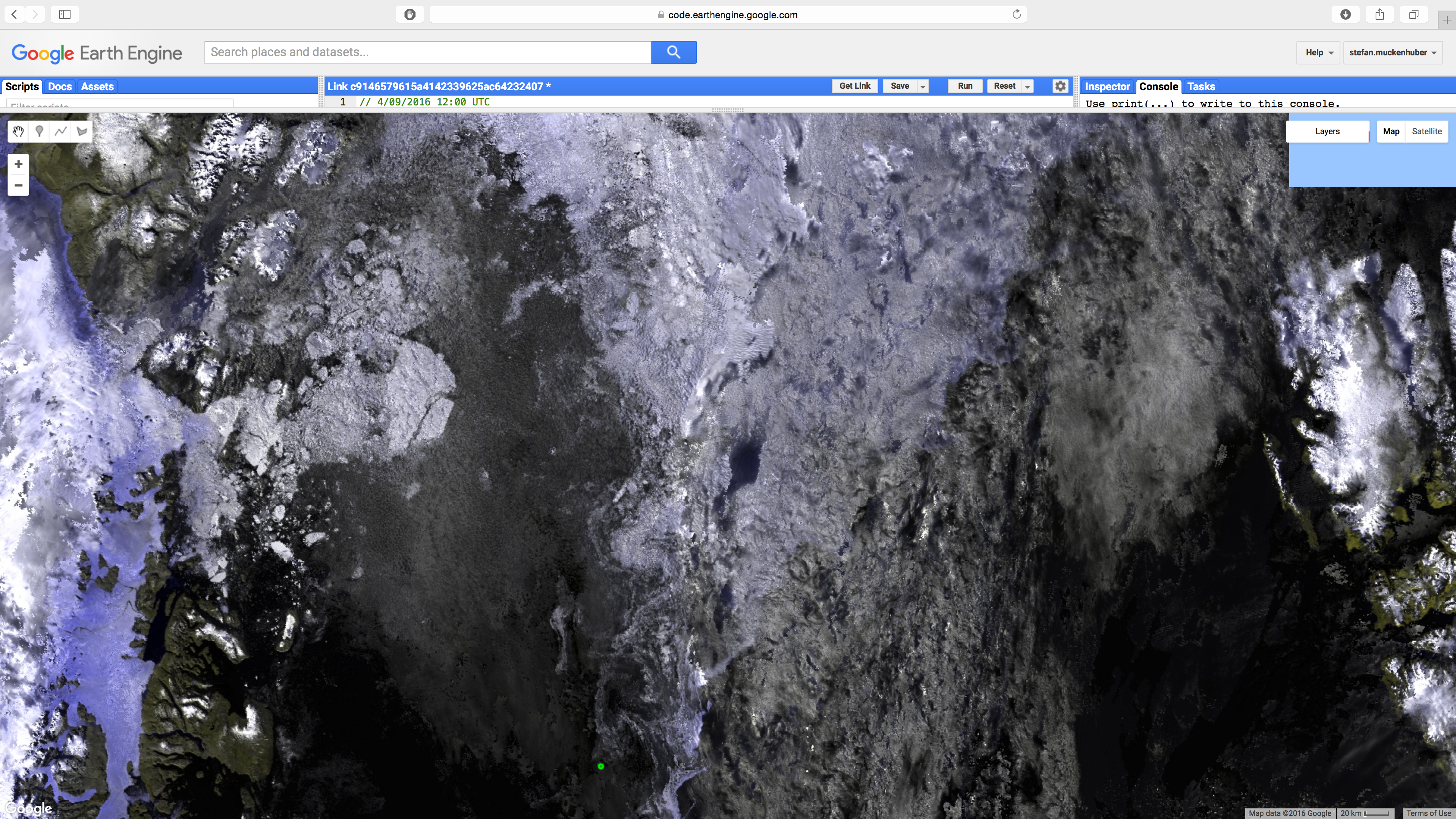

Supplement: Supplementary file 2 — Supplementary material [file mmc2.zip › GPS_tracker_data_python_plots_satellite/GPS_tracker_sat_data/MODIS_EE/MODIS_20160904.png]

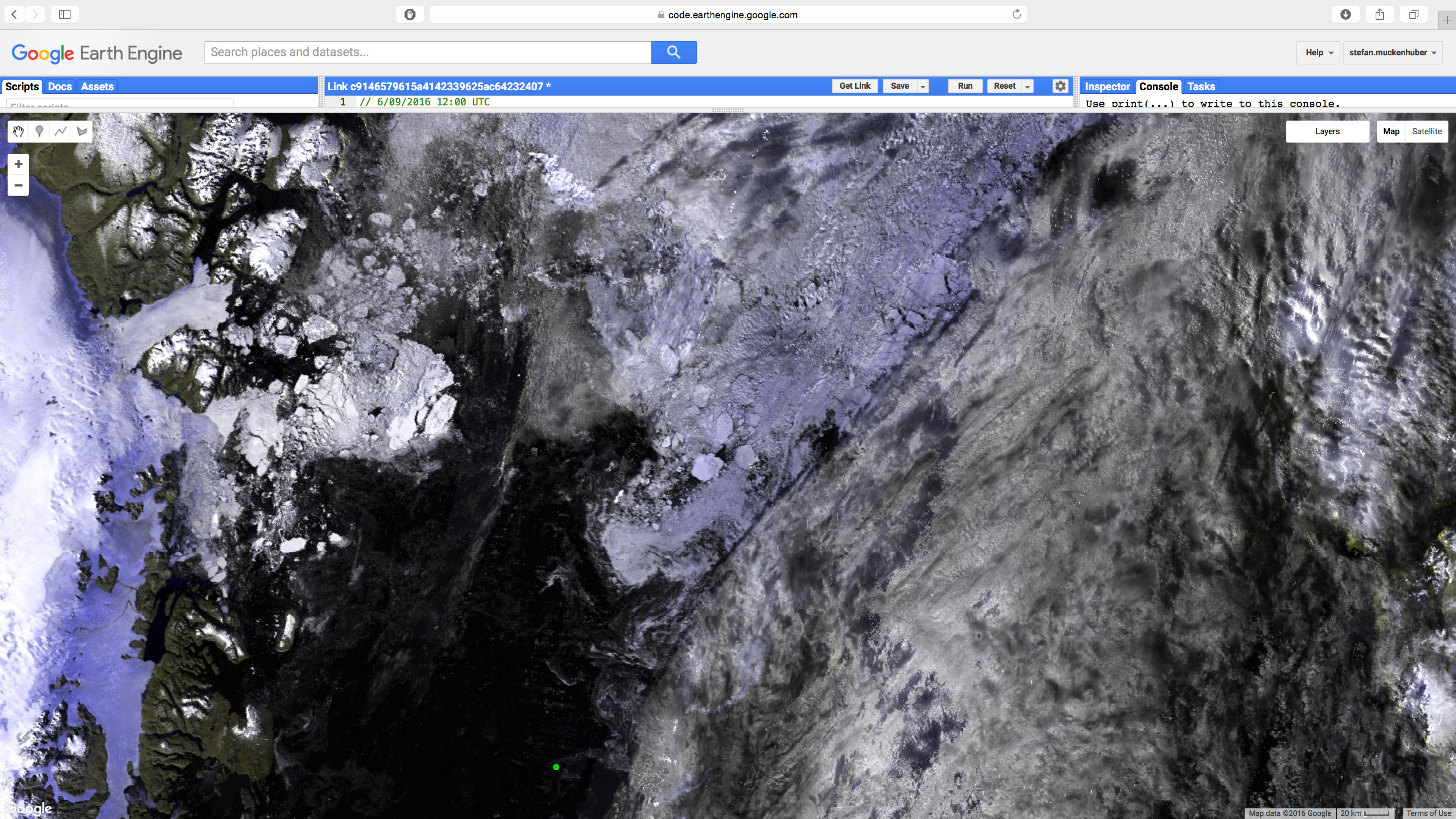

Supplement: Supplementary file 2 — Supplementary material [file mmc2.zip › GPS_tracker_data_python_plots_satellite/GPS_tracker_sat_data/MODIS_EE/MODIS_20160906.png]

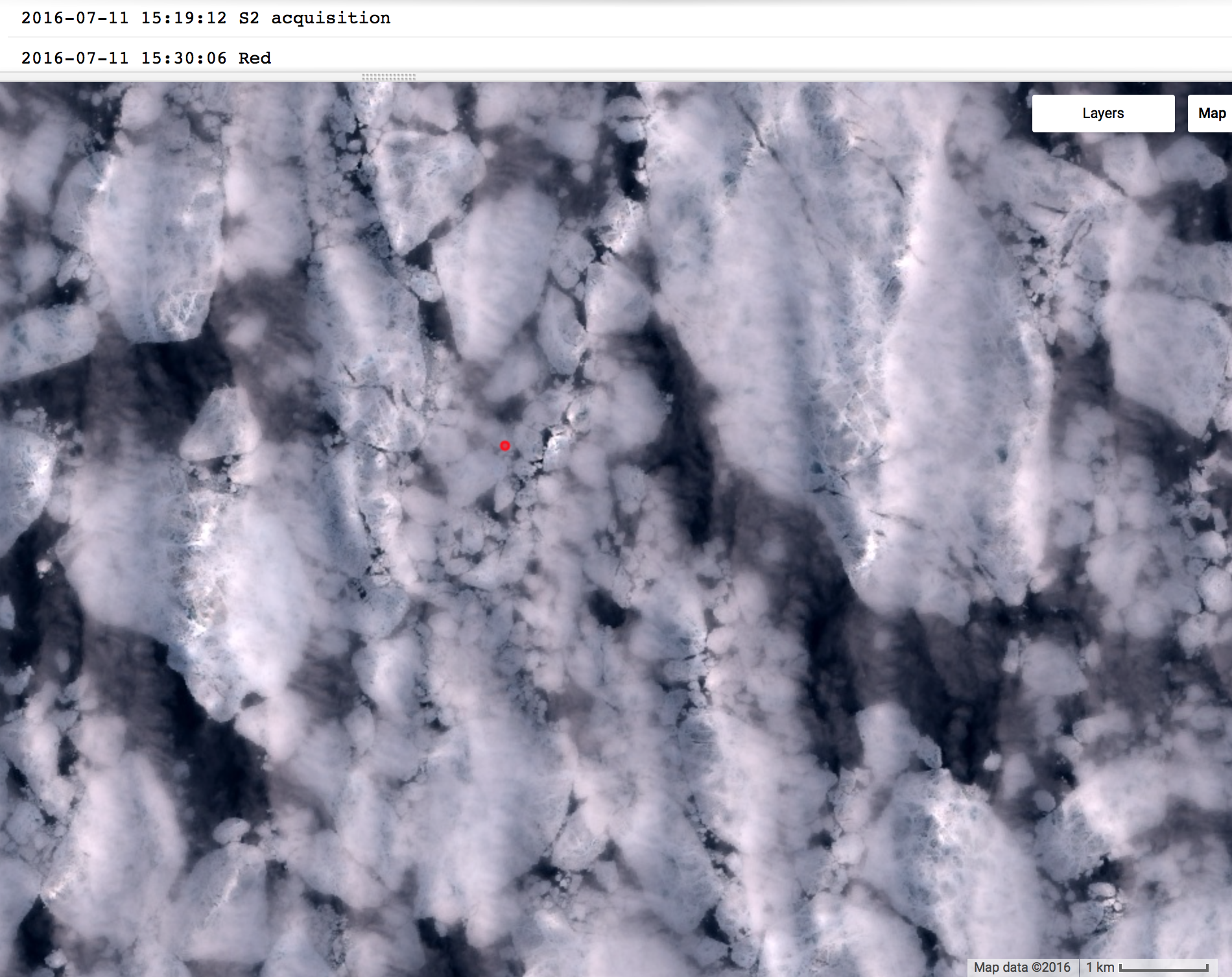

Supplement: Supplementary file 2 — Supplementary material [file mmc2.zip › GPS_tracker_data_python_plots_satellite/GPS_tracker_sat_data/S2_20160711_r.png]

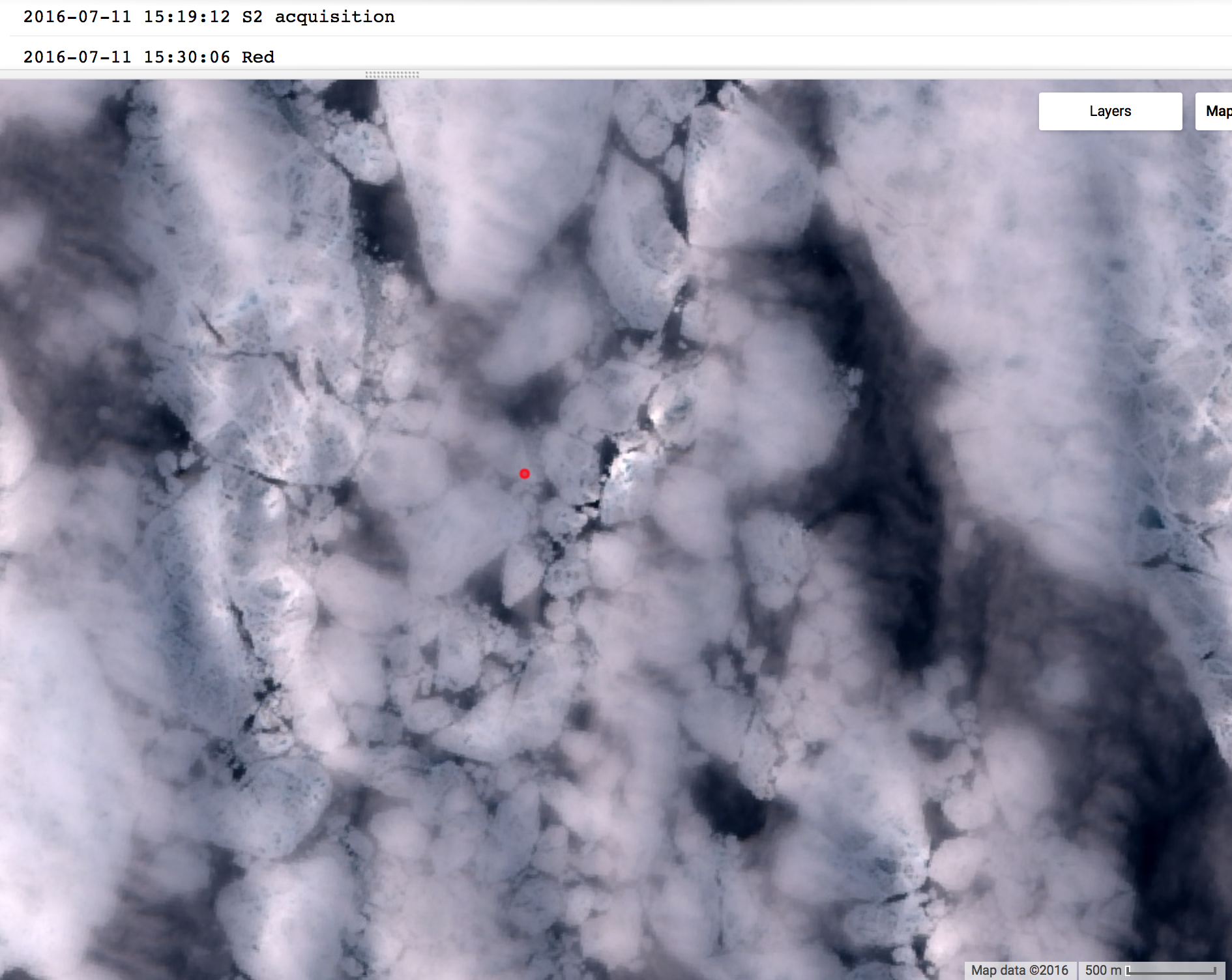

Supplement: Supplementary file 2 — Supplementary material [file mmc2.zip › GPS_tracker_data_python_plots_satellite/GPS_tracker_sat_data/S2_20160711_r_zoom.png]
